# Supplementary material for: Seleno-Analogs of Scaffolds Resembling Natural Products a Novel Warhead toward Dual Compounds
Source: Antioxidants (Basel). 2023 Jan 6;12(1):139. doi: 10.3390/antiox12010139 (PMC9854712; doi:10.3390/antiox12010139)
Supplement: Supplementary file 1 [file antioxidants-12-00139-s001.zip › antioxidants-2138591-supplementary.pdf]

Seleno-Analogs of Scaffolds Resembling Natural Products a

Novel Warhead toward Dual Compounds

Nora Astrain-Redin [1], Irene Talavera [1], Esther Moreno [1], María J. Ramírez [2,*], Nuria Martínez-Sáez [1], Ignacio Encío [3], Arun K. Sharma [4], Carmen Sanmartín [1], Daniel Plano [1,*]

[1] Departamento de Tecnología y Química Farmacéuticas, Facultad de Farmacia y Nutrición, Universidad de Navarra, Irunlarrea 1, E-31008 Pamplona, Spain
[2] Departamento de Farmacología y Toxicología, Facultad de Farmacia y Nutrición, Universidad de Navarra, Irunlarrea 1, E-31008 Pamplona, Spain
[3] Departamento de Ciencias de la Salud, Universidad Pública de Navarra, Avda. Barañain s/n, E-31008 Pamplona, Spain
[4] Department of Pharmacology, Penn State Cancer Institute, CH72, Penn State College of Medicine, 500 University Drive, Hershey, PA 17033, USA
* Correspondence: mariaja@unav.es (M.J.R.); dplano@unav.es (D.P.)

Table of Contents

**Biological evaluation**	
**Cytotoxicity screening**	**Table S1**
**DPPH radical scavenging assay**	**Table S2-S3**
**NCI-60 Analysis**	
**14a (One-Dose Assay)**	**Figure S1**
**17a (One-Dose Assay)**	**Figure S2**
**14a (Five-Dose Assay)**	**Figure S3**
**17a (Five-Dose Assay)**	**Figure S4**
**Spectroscopic characterization**	
**^{1}H, ^{13}C and ^{77}Se NMR spectra**	**Figures S5-S119**

Table S1. Cell growth % at 10 µM for the compounds **1a-19a** and **1b-19b** in HTB-54, DU-145, HT-29, and MDA-MB-231.

Comp. Series a	Cancer cell lines				Comp. Series b	Cancer cell lines			
	HTB-54	MDA-MB-231	DU-145	HT-29		HTB-54	MDA-MB-231	DU-145	HT-29
**1a**	72.1 ± 8.1	92.6 ± 12.7	> 100	91.0 ± 3.6	**1b**	86.0 ± 11.6	71.3 ± 8.0	62.7 ± 7.7	87.9 ± 12.1
**2a**	43.2 ± 9.0	37.9 ±3.6	65.6 ± 5.6	85.3 ± 19.4	**2b**	78.3 ± 11.4	79.3 ± 18.7	72.9 ± 13.6	78.3 ± 6.9
**3a**	37.5 ± 7.3	29.7 ± 6.4	39.8 ± 6.3	63.2 ± 16.9	**3b**	> 100	93.2 ± 10.2	41.6 ± 3.5	81.8 ± 15.2
**4a**	67.6 ± 15.1	97.3 ± 10.4	> 100	33.6 ± 12.6	**4b**	57.8 ± 8.1	62.6 ± 7.4	19.7 ± 2.2	50.6 ± 7.8
**5a**	46.1 ± 5.6	91.1 ± 16.8	83.3 ± 7.8	87.6 ± 7.9	**5b**	79.8 ± 7.7	64.8 ± 14.7	70.5 ± 11.5	80.8 ± 8.5
**6a**	36.3 ± 7.0	35.9 ± 2.6	26.9 ± 4.4	63.7 ± 12.3	**6b**	83.4 ± 7.7	78.9 ± 8.4	76.6 ± 12.0	77. 3 ± 11.6
**7a**	22.0 ± 5.1	35.3 ± 6.4	51.7 ± 9.8	64.3 ± 14.1	**7b**	91.4 ± 6.6	91.3 ± 8.3	54.6 ± 4.5	> 100
**8a**	25.8 ± 5.2	37.1 ± 4.7	49.5 ± 6.6	62.2 ± 16.1	**8b**	99.4 ± 16.2	92.9 ± 16.5	77.1 ± 13.9	87.7 ± 10.1
**9a**	64.8 ± 8.5	89.4 ± 4.9	48.6 ± 6.2	99.5 ± 8.3	**9b**	58.9 ± 7.8	70.2 ± 6.9	22.3 ± 2.7	76.0 ± 13.5
**10a**	27.1 ± 11.2	41.3 ± 3.5	33.4 ± 6.4	61.5 ± 17.6	**10b**	81.2 ± 11.6	62.5 ± 7.7	66.6 ± 8.7	80.2 ± 10.9
**11a**	30.7 ± 6.2	34.1 ± 10.6	35.3 ± 5.8	79.8 ± 16.3	**11b**	75.4 ± 15.5	99.0 ± 11.2	70.0 ± 4.5	> 100
**12a**	95.5 ± 5.9	96.8 ± 15.8	62.7 ± 4.7	70.7 ± 10.7	**12b**	97.6 ± 7.1	92.5 ± 15.1	95.1 ± 12.3	81.3 ± 11.1
**13a**	40.9 ± 4.9	77.1 ± 12.6	29.5 ± 4.3	88.8 ± 8.6	**13b**	82.1 ± 7.6	52.3 ± 7.9	26.8 ± 5.5	70.2 ± 13.2
**14a**	34.2 ± 6.2	38.8 ± 3.6	16.9 ± 8.6	53.9 ± 5.3	**14b**	78.9 ± 9.0	52.3 ± 7.1	25.5 ± 6.2	65.6 ± 7.3
**15a**	38.0 ± 7.1	38.0 ± 5.4	19.1 ± 7.0	49.0 ± 7.2	**15b**	95.6 ± 5.7	59.7 ± 15.7	34.7 ± 6.3	59.3 ± 4.0
**16a**	30.9 ± 6.7	33.8 ± 5.6	18.8 ± 6.3	49.9 ± 5.6	**16b**	> 100	29.8 ± 9.6	66.4 ± 4.4	65.7 ± 10.0
**17a**	21.9 ± 6.5	26.5 ± 5.6	16.3 ± 8.4	38.7 ± 6.0	**17b**	85.9 ± 5.1	59.1 ± 11.5	19.2 ± 4.0	66.3 ± 10.2
**18a**	40.9 ± 18.5	85.9 ± 9.5	74.7 ± 13.7	71.5 ± 11.7	**18b**	62.3 ± 8.3	99.0 ± 11.2	32.6 ± 4.6	66.5 ± 9.5
**19a**	16.3 ± 7.6	81.6 ± 9.7	17.0 ± 2.9	46.0 ± 8.7	**19b**	43.6 ± 18.4	92.8 ± 10.2	25.2 ± 2.2	61.6 ± 9.8

The cell growth % is presented as the mean ± SD of three independent experiments performed in triplicates

Table S2. DPPH radical scavenging activity (%) for the compounds **1a-19a** and **1b-19b** at 0.03 mg/mL in the time range of 0, 5, 15, 30, 60, and 90 minutes.

**0.03 mg/mL**	**DPPH scavenging activity (%)**					
**Compd.**	0 min	5 min	15 min	30 min	60 min	90 min
**Asc. Acid**	96.2 ± 0.4	96.0 ± 0.3	96.0 ± 0.2	96.1 ± 0.3	96.0 ± 0.1	96.0 ± 0.2
**Trolox**	95.3 ± 0.2	95.7 ± 0.1	95.6 ± 0.2	95.7 ± 0.2	95.9 ± 0.2	95.7 ± 0.2
**1a**	1.7 ± 1.3	1.6 ± 1.1	3.4 ± 8.8	6.0 ± 4.9	9.9 ± 1.1	9.6 ± 0.4
**2a**	3.7 ± 0.7	3.0 ± 0.6	9.5 ± 0.3	9.6 ± 0.1	8.8 ± 0.9	9.0 ± 1.2
**3a**	3.6 ± 1.6	3.1 ± 1.2	7.9 ± 0.8	8.1 ± 0.8	8.0 ± 1.3	7.8 ± 1.7
**4a**	0.0 ± 2.4	0.0 ± 2.3	0.0 ± 2.8	0.0 ± 2.4	0.0 ± 2.4	0.0 ± 2.3
**5a**	2.6 ± 1.5	2.3 ± 1.1	3.1 ± 3.9	2.7 ± 3.6	3.5 ± 5.2	3.6 ± 5.3
**6a**	2.3 ± 1.1	1.6 ± 1.0	2.7 ± 4.2	2.1 ± 4.4	2.4 ± 5.6	2.4 ± 5.6
**7a**	3.4 ± 1.5	2.9 ± 1.1	5.8 ± 1.7	5.7 ± 1.9	6.1 ± 2.9	6.3 ± 2.9
**8a**	1.8 ± 0.2	2.0 ± 0.1	3.7 ± 4.8	3.5 ± 4.7	3.8 ± 6.2	3.7 ± 6.6
**9a**	3.3 ± 0.9	2.7 ± 0.7	7.8 ± 9.6	7.4 ± 9.3	7.6 ± 10.0	7.8 ± 10.4
**10a**	3.5 ± 0.2	2.4 ± 0.2	6.8 ± 8.5	7.2 ± 8.4	8.0 ± 6.2	8.6 ± 6.8
**11a**	3.1 ± 1.1	2.6 ± 0.8	4.9 ± 5.9	5.4 ± 5.7	5.1 ± 6.6.	4.6 ± 7.5
**12a**	1.6 ± 0.9	1.3 ± 0.6	7.3 ± 4.7	6.7 ± 6.0	7.2 ± 4.5	6.1 ± 6.7
**13a**	0.0 ± 0.4	0.0 ± 0.4	4.7 ± 1.5	8.5 ± 0.7	16.2 ± 0.7	22.4 ± 0.8
**14a**	0.0 ± 1.4	0.0 ± 2.2	0.0 ± 2.3	0.0 ± 1.9	0.0 ± 1.5	0.0 ± 1.4
**15a**	0.0 ± 3.5	0.0 ± 4.2	0.0 ± 2.9	0.0 ± 3.8	0.0 ± 3.5	0.0 ± 3.5
**16a**	0.0 ± 3.9	0.0 ± 5.0	0.0 ± 3.6	0.0 ± 4.7	0.0 ± 4.2	0.0 ± 3.9
**17a**	0.0 ± 3.5	0.0 ± 4.4	0.0 ± 3.3	0.0 ± 3.2	0.0 ± 3.8	0.0 ± 3.1
**18a**	0.0 ± 3.0	0.0 ± 3.8	0.0 ± 4.0	0.0 ± 3.7	0.0 ± 3.5	0.0 ± 3.5
**19a**	0.0 ± 3.7	0.0 ± 3.8	1.6 ± 4.6	0.0 ± 4.5	0.0 ± 4.4	0.0 ± 4.2
**1b**	0.0 ± 0.1	5.7 ± 5.2	5.5 ± 4.9	0.8 ± 4.7	1.6 ± 4.3	2.9 ± 2.2
**2b**	0.2 ± 0.2	5.4 ± 6.2	5.1 ± 6.5	2.7 ± 1.0	4.1 ± 0.9	3.5 ± 0.9
**3b**	0.0 ± 0.6	3.6 ± 6.7	4.0 ± 6.5	0.7 ± 1.7	1.5 ± 1.5	0.7 ± 1.6
**4b**	1.8 ± 0.8	3.3 ± 1.6	4.1 ± 1.5	4.7 ± 9.8	4.4 ± 8.1	4.2 ± 8.0
**5b**	0.0 ± 0.5	7.6 ± 2.1	8.0 ± 2.4	1.0 ± 2.1	2.3 ± 2.1	2.4 ± 2.0
**6b**	0.0 ± 2.0	8.0 ± 1.1	8.3 ± 1.1	4.1 ± 3.6	4.9 ± 3.9	4.0 ± 3.3
**7b**	0.0 ± 3.4	7.1 ± 2.2	7.6 ± 2.2	4.4 ± 0.8	6.4 ± 1.0	7.4 ± 0.7
**8b**	0.0 ± 0.3	7.8 ± 2.7	8.4 ± 2.9	6.3 ± 1.5	7.5 ± 1.6	7.3 ± 1.7
**9b**	0.0 ± 2.1	0.0 ± 2.1	0.0 ± 2.2	0.0 ± 2.2	0.0 ± 2.3	0.0 ± 2.1
**10b**	0.0 ± 3.4	0.0 ± 3.5	0.0 ± 3.6	0.0 ± 3.5	0.0 ± 3.3	0.0 ± 3.2
**11b**	1.3 ± 0.3	4.2 ± 3.1	4.2 ± 3.3	3.8 ± 0.6	5.9 ± 3.0	5.4 ± 2.3
**12b**	0.0 ± 1.1	0.0 ± 0.7	0.0 ± 1.6	0.0 ± 1.3	1.0 ± 1.6	1.9 ± 2.0
**13b**	0.0 ± 0.9	0.0 ± 1.2	0.4 ± 1.2	1.0 ± 1.3	3.8 ± 1.2	4.6 ± 1.1
**14b**	0.0 ± 0.6	0.0 ± 0.6	0.0 ± 1.2	0.0 ± 0.6	0.0 ± 0.6	0.0 ± 0.5
**15b**	0.0 ± 0.4	0.0 ± 0.7	0.0 ± 1.5	0.0 ± 0.4	0.0 ± 0.5	0.0 ± 1.0
**16b**	0.0 ± 0.9	0.0 ± 0.4	0.0 ± 0.6	0.0 ± 0.5	0.0 ± 0.4	0.0 ± 0.6
**17b**	0.0 ± 0.9	0.0 ± 0.8	0.0 ± 1.3	0.0 ± 1.1	0.0 ± 1.1	0.0 ± 1.5
**18b**	0.0 ± 1.4	0.0 ± 1.5	0.0 ± 1.7	0.0 ± 1.1	0.0 ± 1.3	0.0 ± 1.6
**19b**	0.0 ± 1.0	0.0 ± 0.9	0.0 ± 0.8	0.0 ± 1.0	0.0 ± 1.0	0.0 ± 0.9

The percentage of inhibition of DPPH is presented as the mean ± SD of three independent experiments performed in triplicates.

Table S3. DPPH radical scavenging activity (%) for compound **13a** at five different concentrations (0.3 – 0.0003 mg/mL) in the time range of 0, 5, 15, 30, 60, and 90 minutes.

		DPPH scavenging activity (%)								
**Comp.**	**Concentration**	0 min	5 min	15 min	30 min	60 min	90 min	120 min	150 min	180 min
**Asc. Acid**	0.0003 mg/mL	3.9 ± 0.9	3.3 ± 0.9	3.5 ± 0.8	3.4 ± 0.8	3.3 ± 1.1	3.1 ± 1.3	2.9 ± 1.5	2.2 ± 1.5	2.0 ± 1.9
	0.003 mg/mL	44.8 ± 0.8	43.3 ± 2.4	43.5 ± 2.3	43.4 ± 2.2	43.2 ± 2.3	43.0 ± 2.3	42.8 ± 2.3	42.8 ± 2.3	42.1 ± 2.6
	0.03 mg/mL	96.4 ± 0.1	93.4 ± 5.1	93.6 ± 5.2	93.6 ± 5.4	93.5 ± 5.4	93.5 ± 5.4	93.3 ± 5.3	93.3 ± 5.3	93.2 ± 5.5
	0.09 mg/mL	96.8 ± 0.4	93.7 ± 5.4	94.0 ± 5.6	94.0 ± 5.8	93.7 ± 5.5	93.7 ± 5.5	93.6 ± 5.5	93.6 ± 5.5	93.5 ± 5.6
	0.3 mg/mL	96.9 ± 0.3	93.7 ± 5.4	94.2 ± 5.3	94.0 ± 5.4	93.8 ± 5.4	93.7 ± 5.4	93.6 ± 5.4	93.5 ± 5.4	93.5 ± 5.5
**Trolox**	0.0003 mg/mL	2.9 ± 0.6	4.2 ± 3.3	3.6 ± 1.3	3.5 ± 1.1	3.3 ± 1.5	3.0 ± 1.7	2.8 ± 1.8	2.1 ± 2.0	1.8 ± 2.2
	0.003 mg/mL	31.6 ± 5.2	32.2 ± 6.1	31.3 ± 6.0	30.8 ± 6.0	31.5 ± 5.6	31.2 ± 5.3	31.0 ± 5.2	30.4 ± 5.4	30.2 ± 5.2
	0.03 mg/mL	91.6 ± 1.2	93.4 ± 5.7	93.5 ± 5.6	93.5 ± 5.5	93.4 ± 5.6	93.4 ± 5.6	93.3 ± 5.6	93.3 ± 5.7	93.2 ± 5.7
	0.09 mg/mL	96.6 ± 0.1	93.6 ± 5.4	93.6 ± 5.3	93.5 ± 5.3	93.5 ± 5.4	93.4 ± 5.4	93.5 ± 5.5	93.4 ± 5.6	93.4 ± 5.6
	0.3 mg/mL	96.6 ± 0.1	93.5 ± 5.3	93.5 ± 5.4	93.4 ± 5.4	93.4 ± 5.5	93.4 ± 5.4	93.4 ± 5.5	93.4 ± 5.7	93.4 ± 5.7
**13a**	0.0003 mg/mL	0.0 ± 1.2	0.0 ± 1.3	0.0 ± 1.4	0.3 ± 1.5	0.7 ± 1.7	1.0 ± 1.9	1.3 ± 2.1	1.3 ± 2.1	1.1 ± 2.3
	0.003 mg/mL	0.2 ± 0.7	0.7 ± 1.0	2.2 ± 0.8	3.9 ± 0.8	6.5 ± 1.0	8.3 ± 1.2	9.8 ± 1.3	10.7 ± 1.6	11.8 ± 1.8
	0.03 mg/mL	0.4 ± 1.9	4.5 ± 1.6	9.8 ± 1.7	15.5 ± 1.9	23.3 ± 2.5	28.7 ± 2.9	33.0 ± 3.2	36.3 ± 3.5	39.4 ± 3.8
	0.09 mg/mL	1.6 ± 1.4	9.5 ± 1.3	18.0 ± 1.4	26.5 ± 1.8	37.5 ± 2.5	44.7 ± 2.9	50.3 ± 3.4	54.4 ± 3.8	58.0 ± 3.9
	0.3 mg/mL	5.7 ± 0.7	19.8 ± 1.9	32.7 ± 2.1	44.2 ± 2.7	57.4 ± 3.4	65.0 ± 3.7	70.1 ± 3.9	73.7 ± 4.3	76.4 ± 4.3

The percentage of inhibition of DPPH is presented as the mean ± SD of three independent experiments performed in triplicates.

Figure S1. NCI-60 results at one dose (10 µM) of compound **14a** after 48 h of treatment.

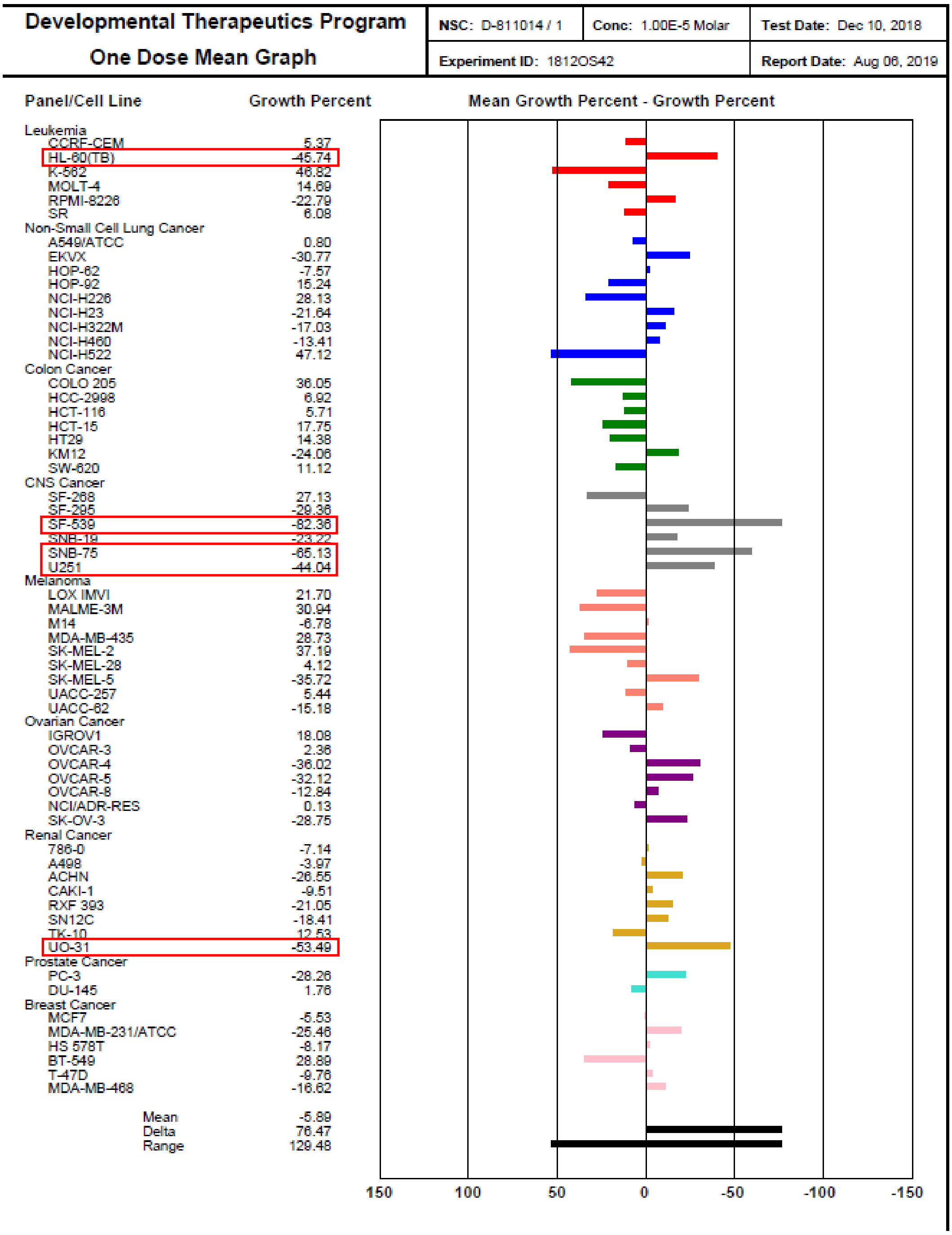

Figure S2. NCI-60 results at one dose (10 µM) of compound **17a** after 48 h of treatment.

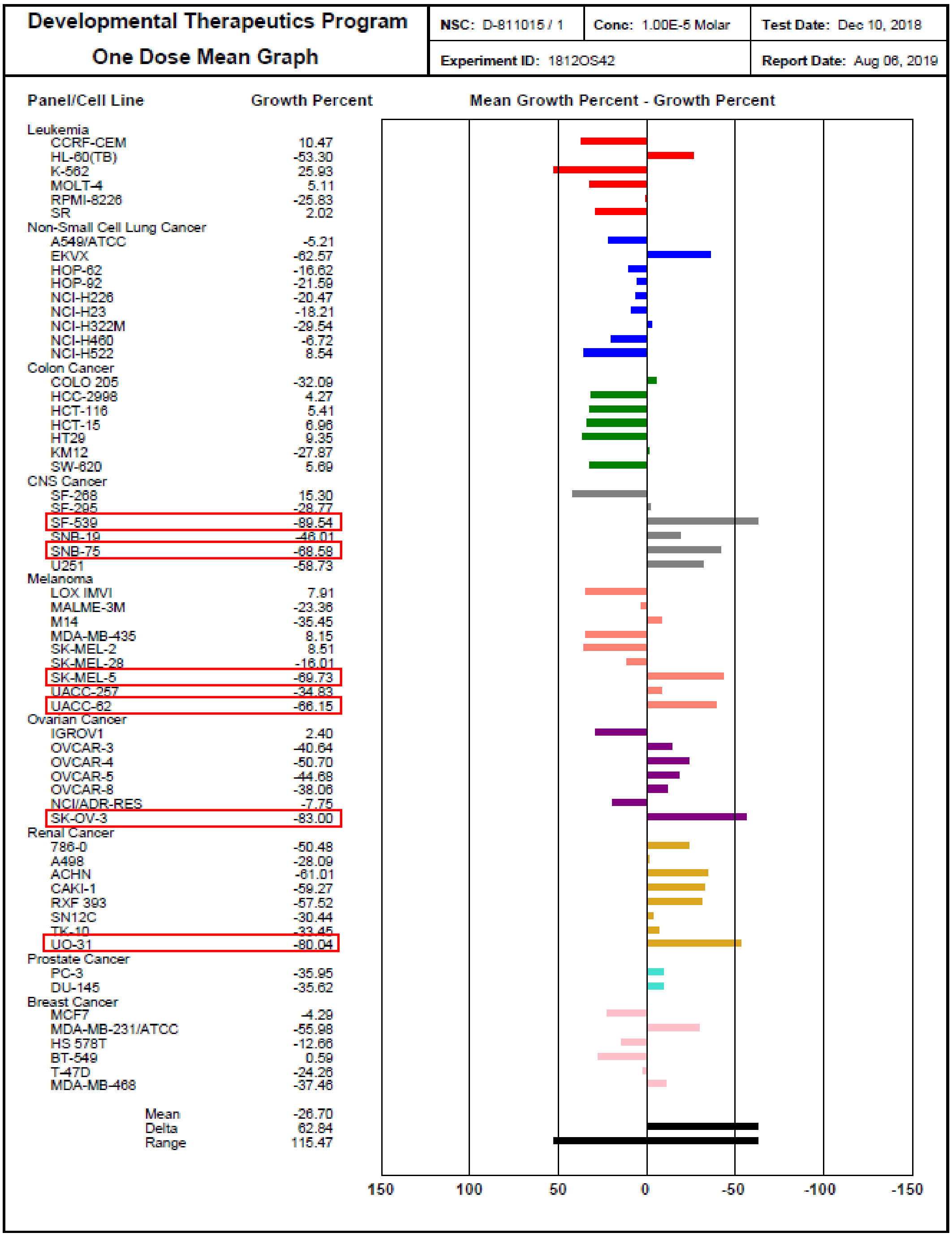

Figure S3. NCI-60 results of GI_{50}, TGI, and LD_{50} values for compound **14a**.

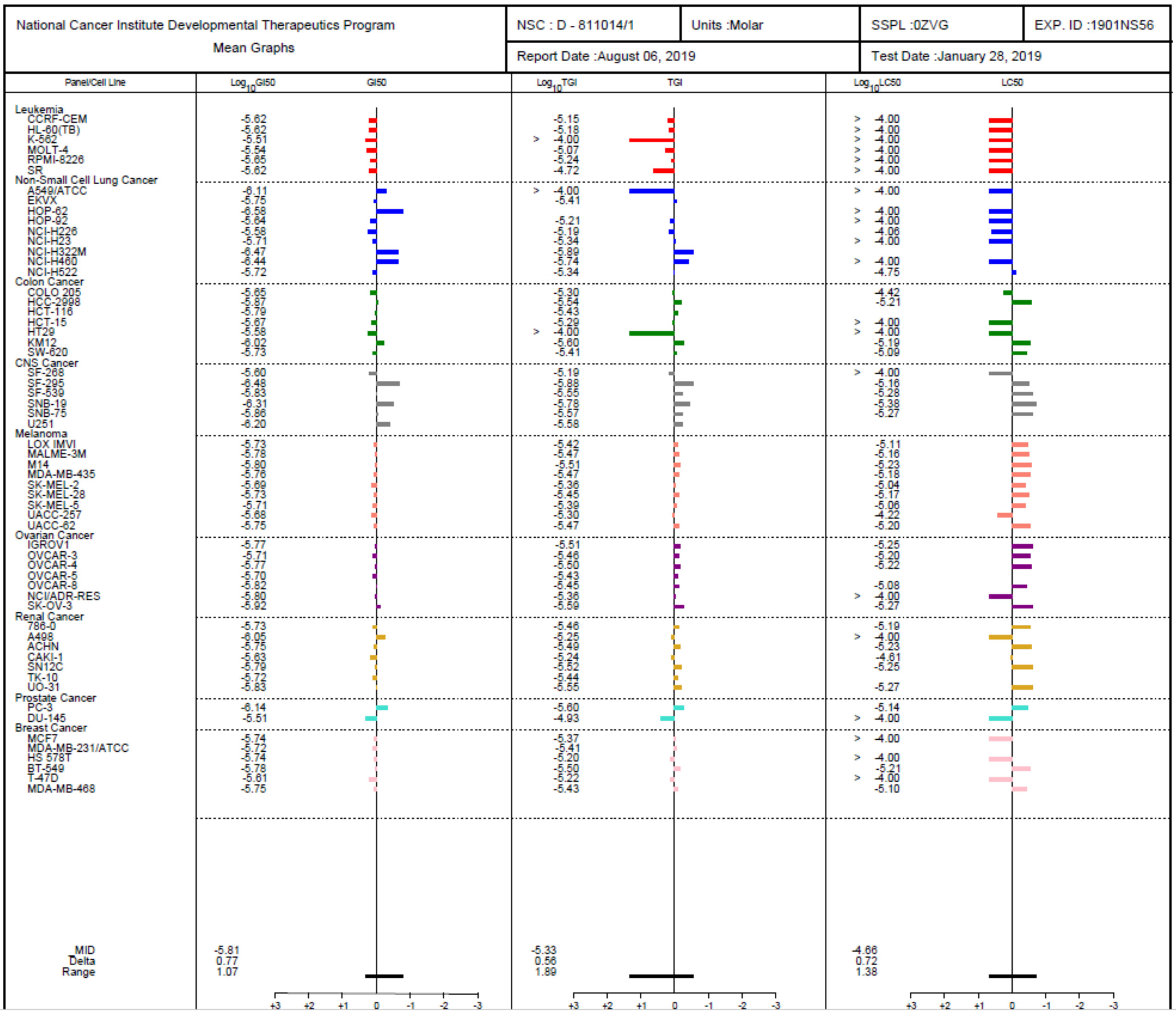

Figure S4. NCI-60 results of GI_{50}, TGI, and LD_{50} values for compound **17a**.

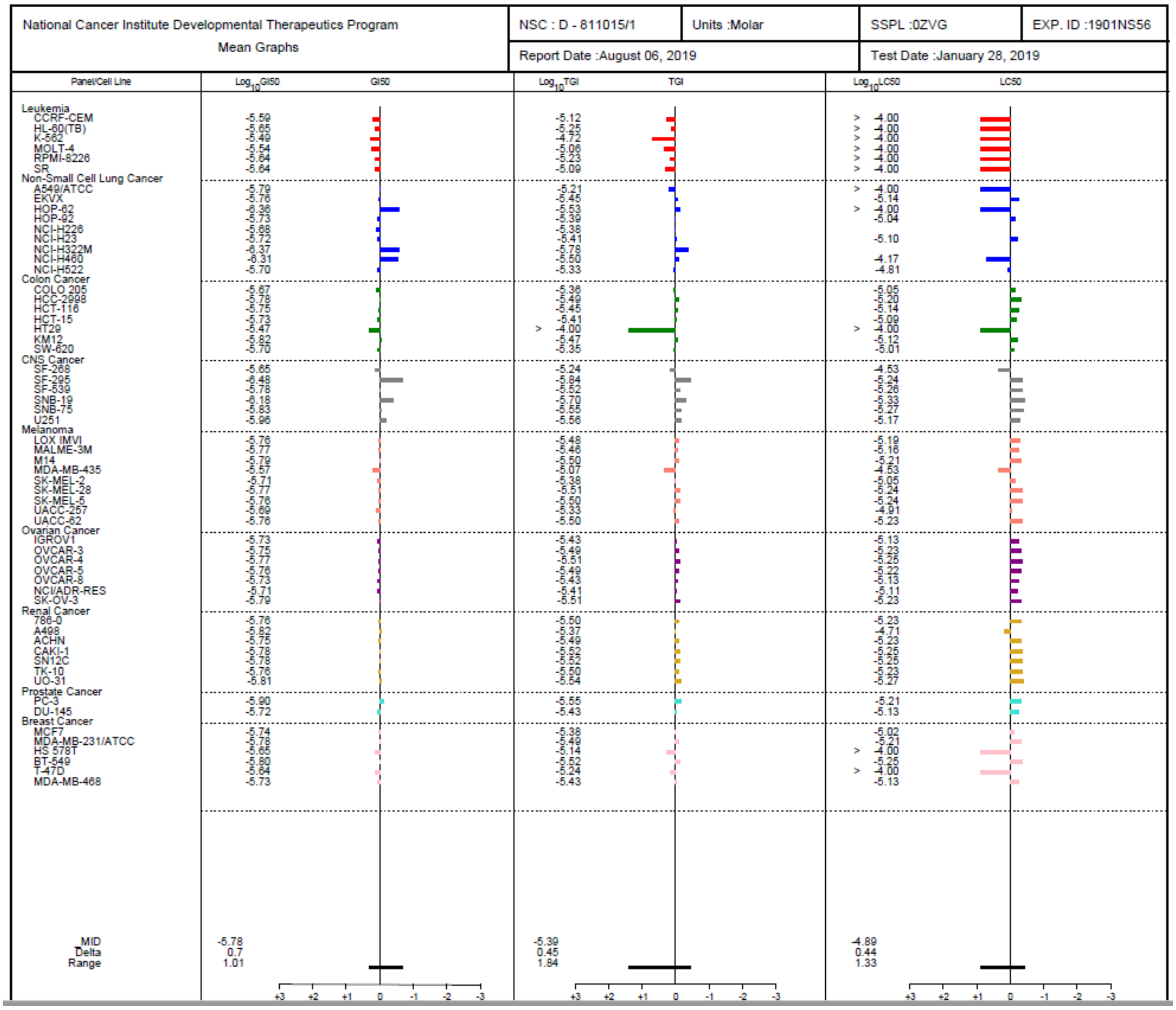

Spectroscopic characterization-NMR spectra

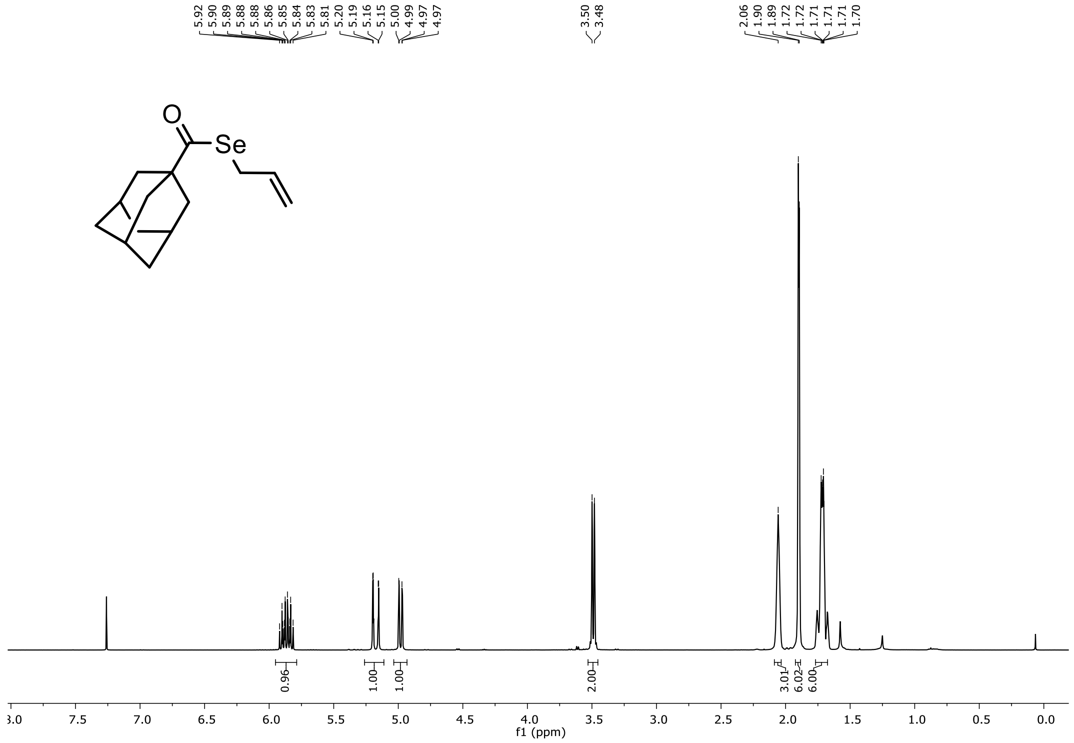

Figure S5. ^{1}H-NMR spectrum of compound **1a**.

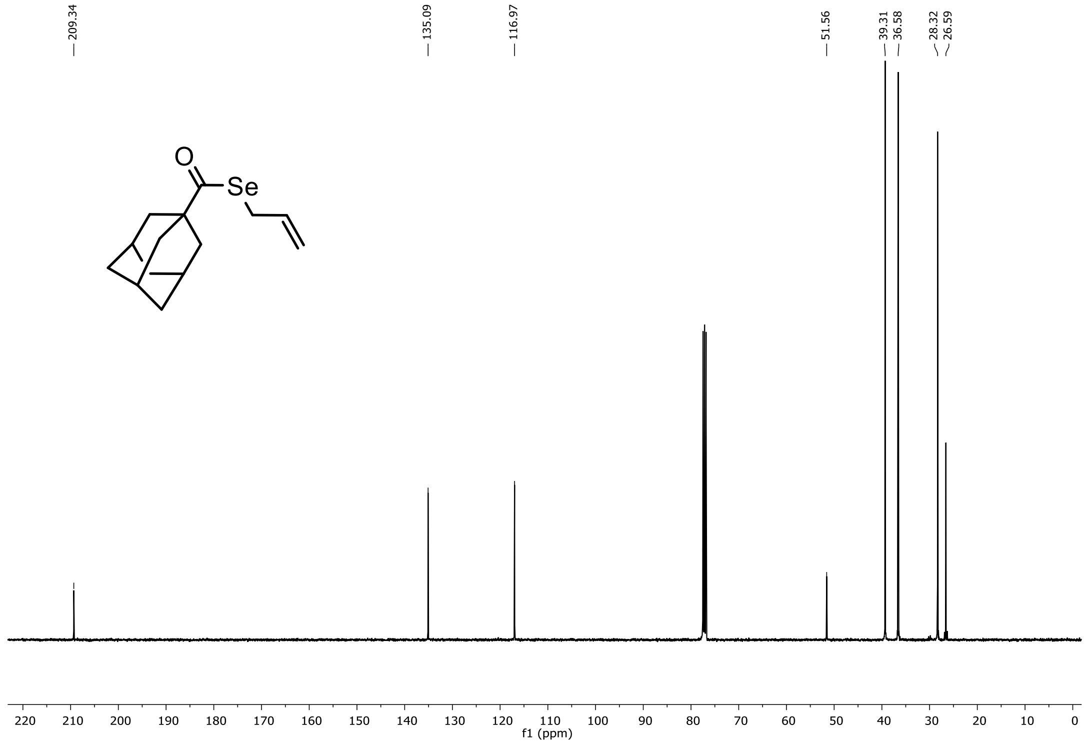

Figure S6. ^{13}C-NMR spectrum of compound **1a**.

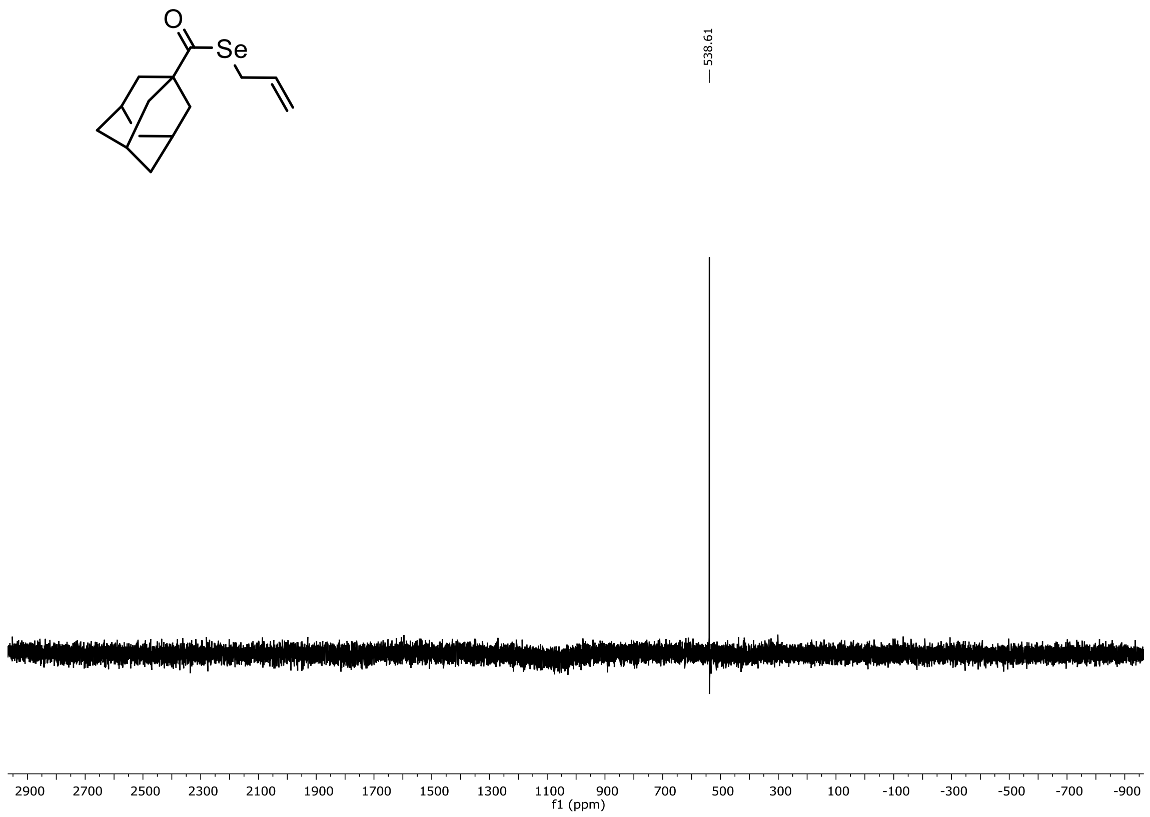

Figure S7. ^{77}Se-NMR spectrum of compound **1a**.

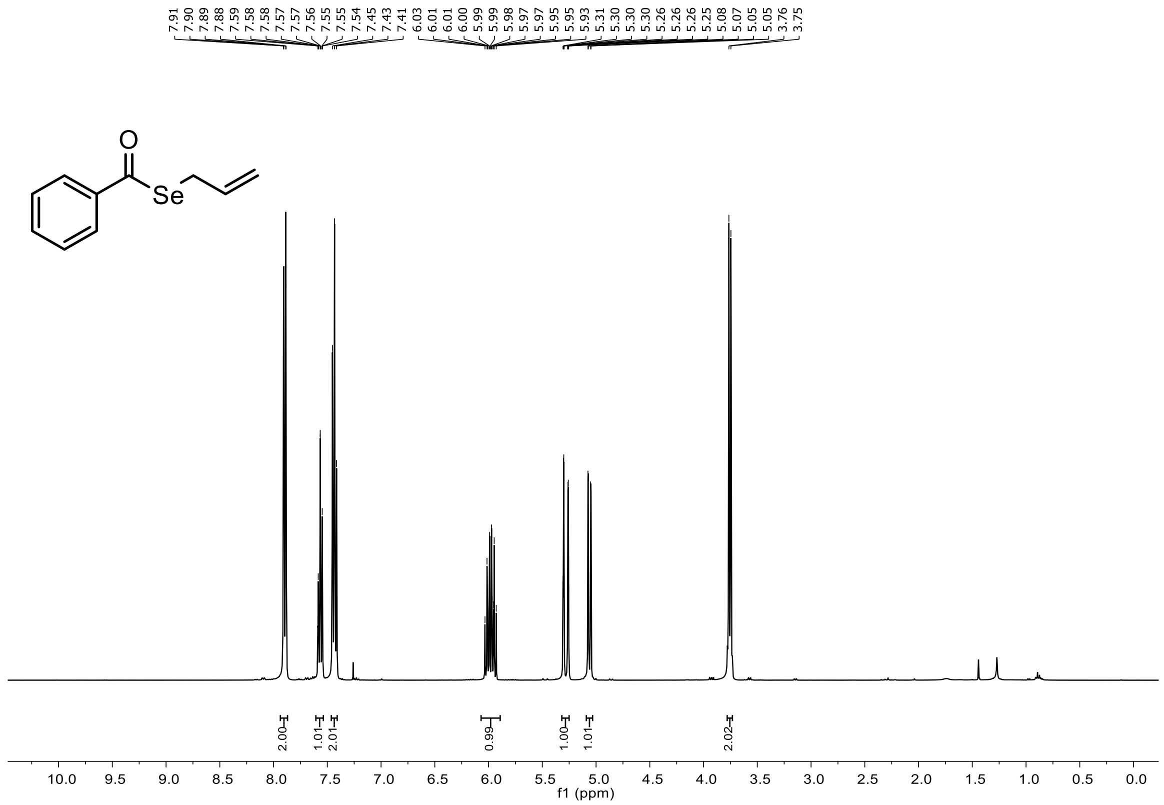

Figure S8. ^{1}H-NMR spectrum of compound **2a**.

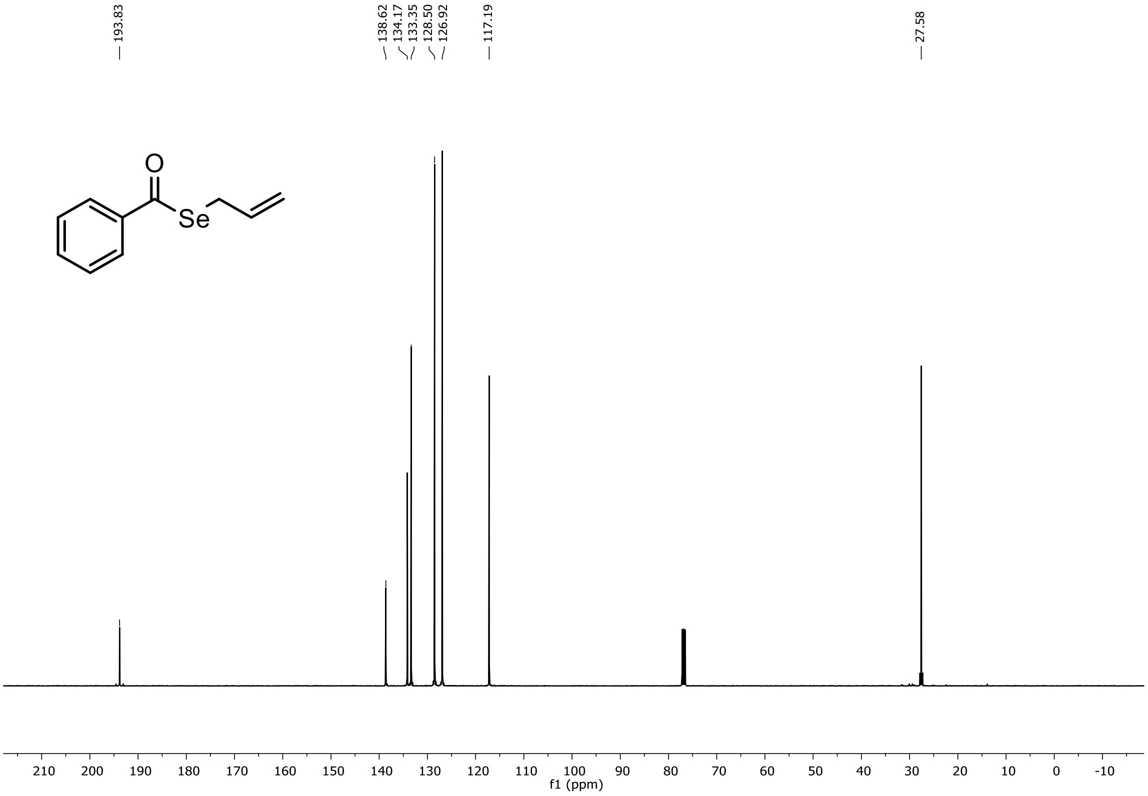

Figure S9. ^{13}C-NMR spectrum of compound **2a.**

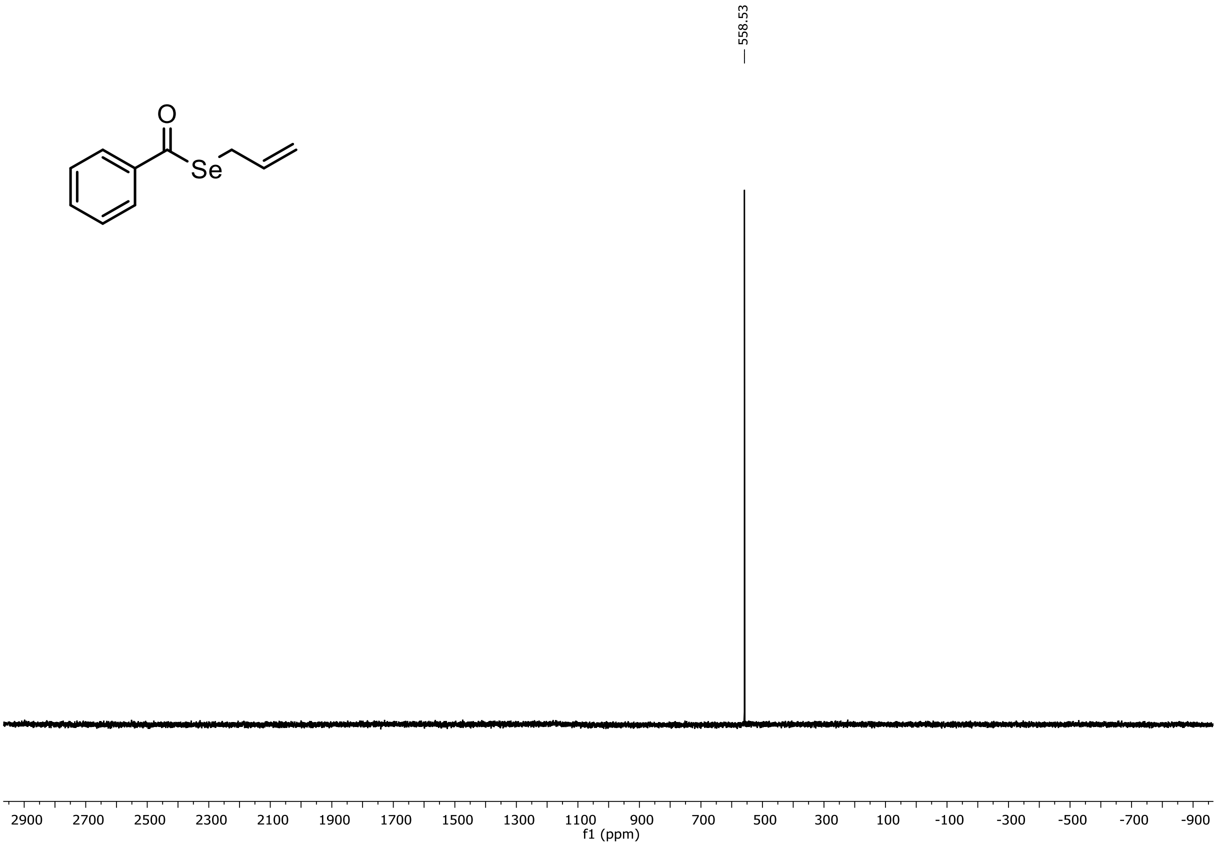

Figure S10. ^{77}Se-NMR spectrum of compound **2a**.

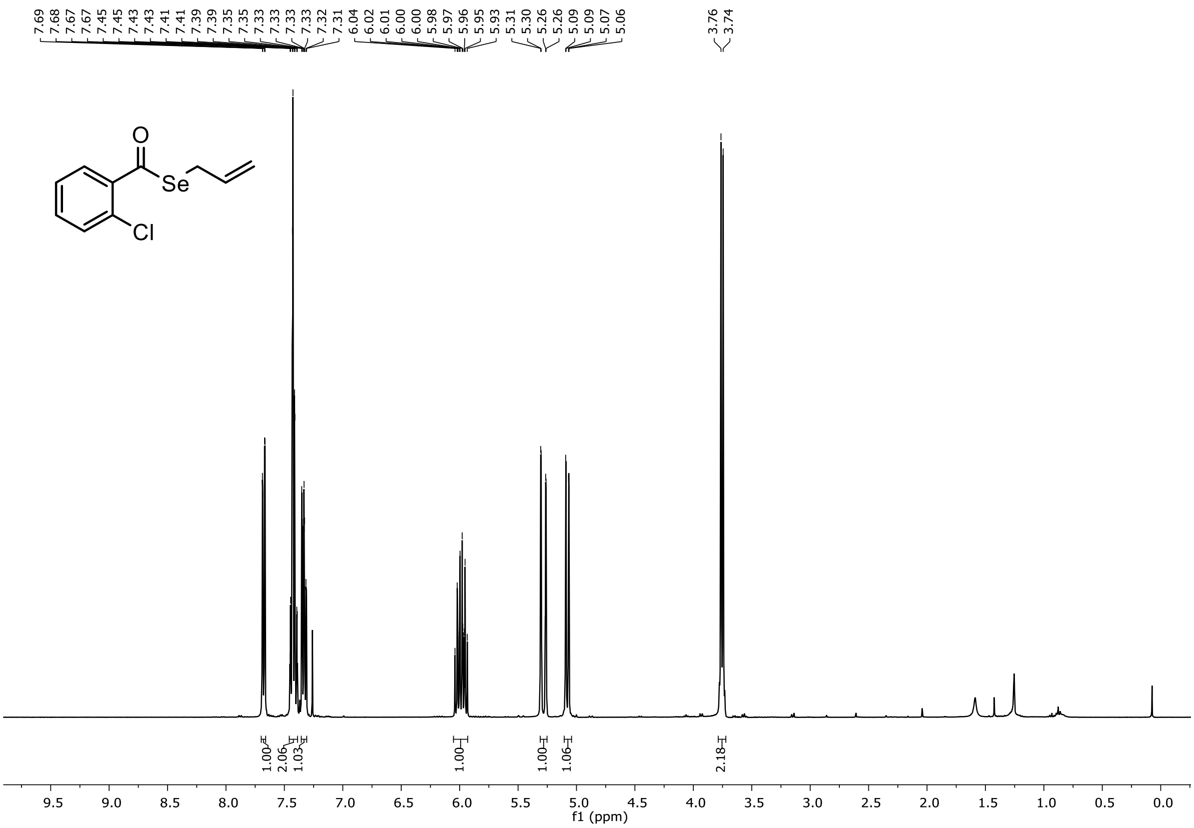

Figure S11. ^{1}H-NMR spectrum of compound **3a**.

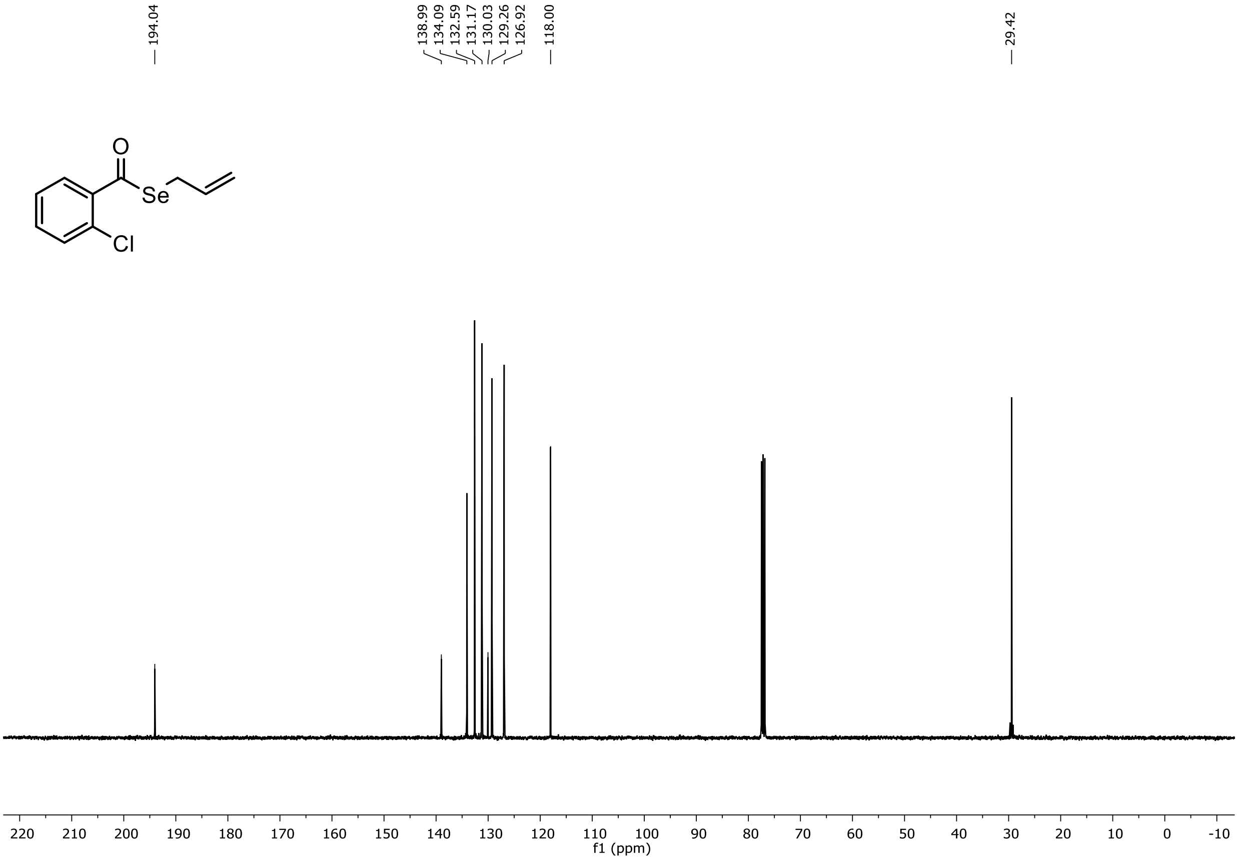

Figure S12. ^{13}C-NMR spectrum of compound **3a.**

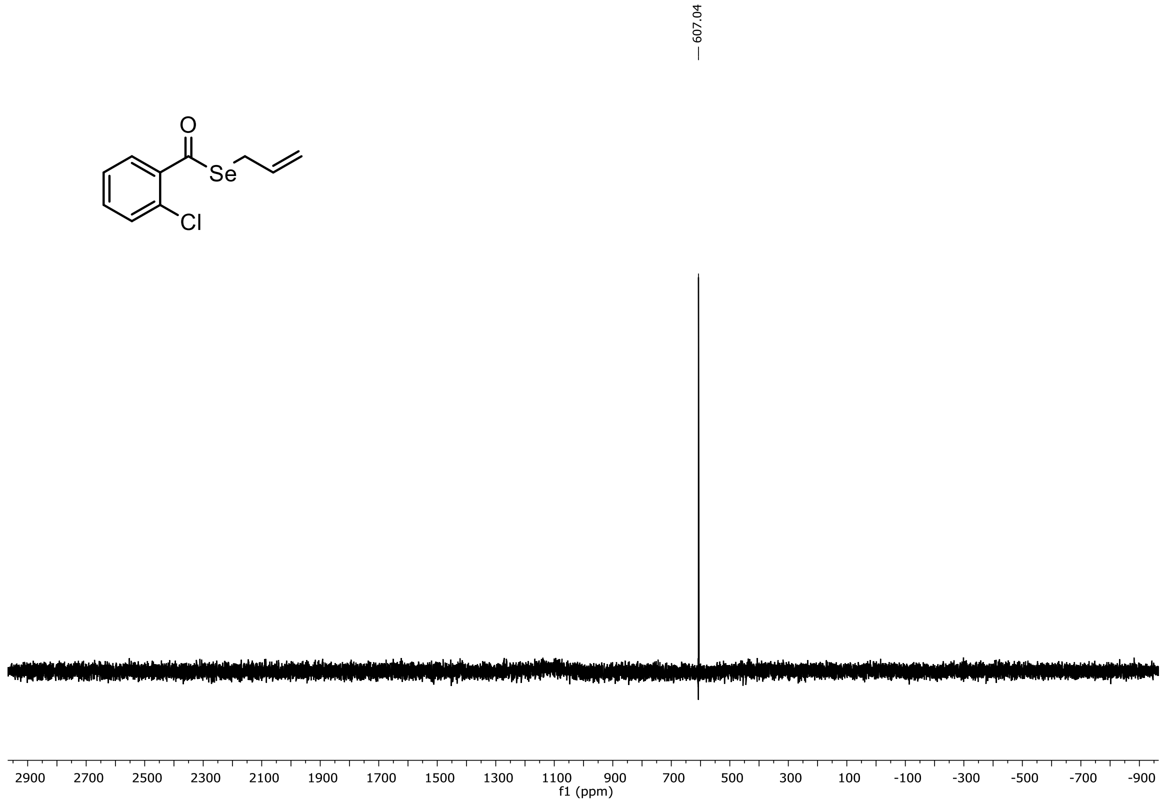

Figure S13. ^{77}Se-NMR spectrum of compound **3a**.

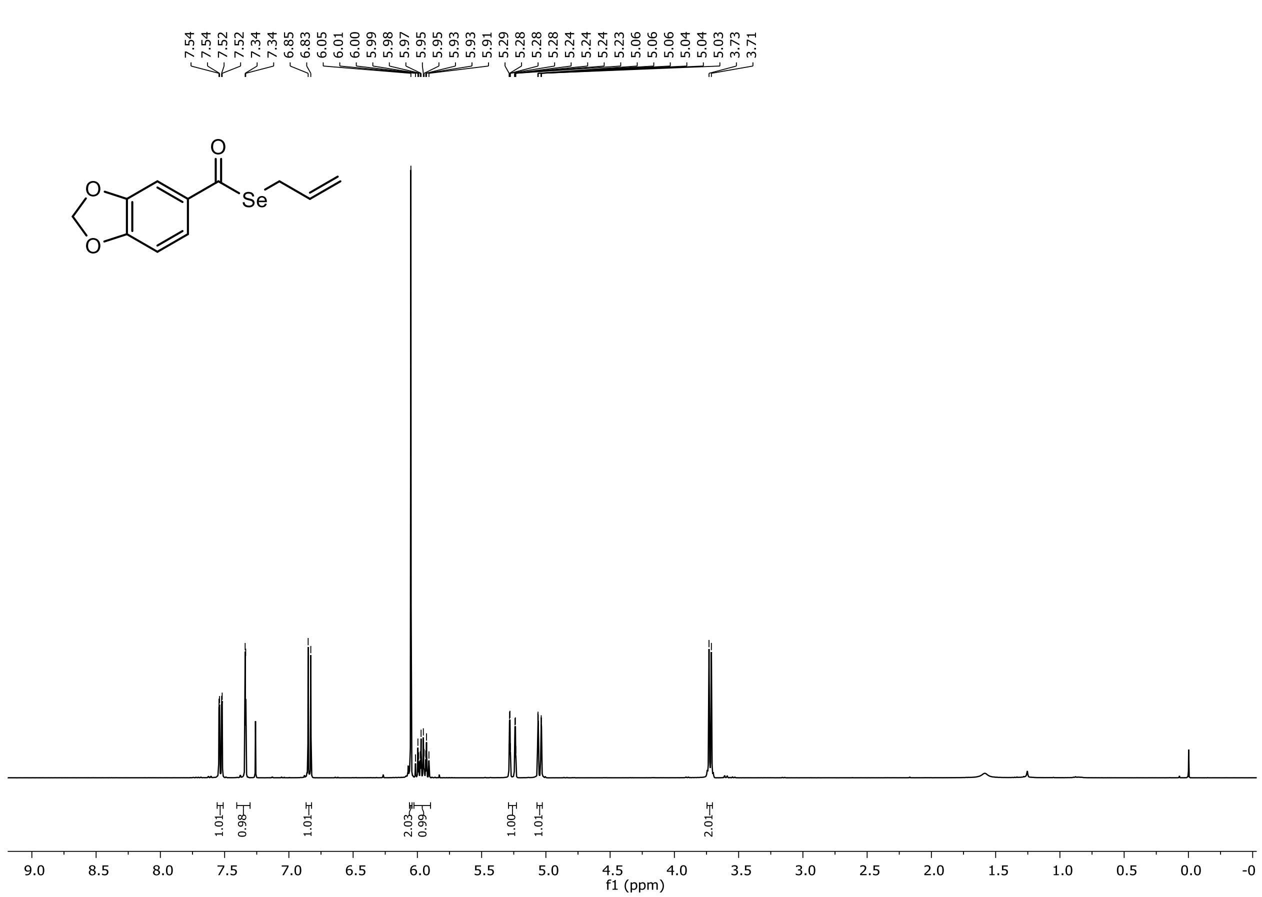

Figure S14. ^{1}H-NMR spectrum of compound **4a**.

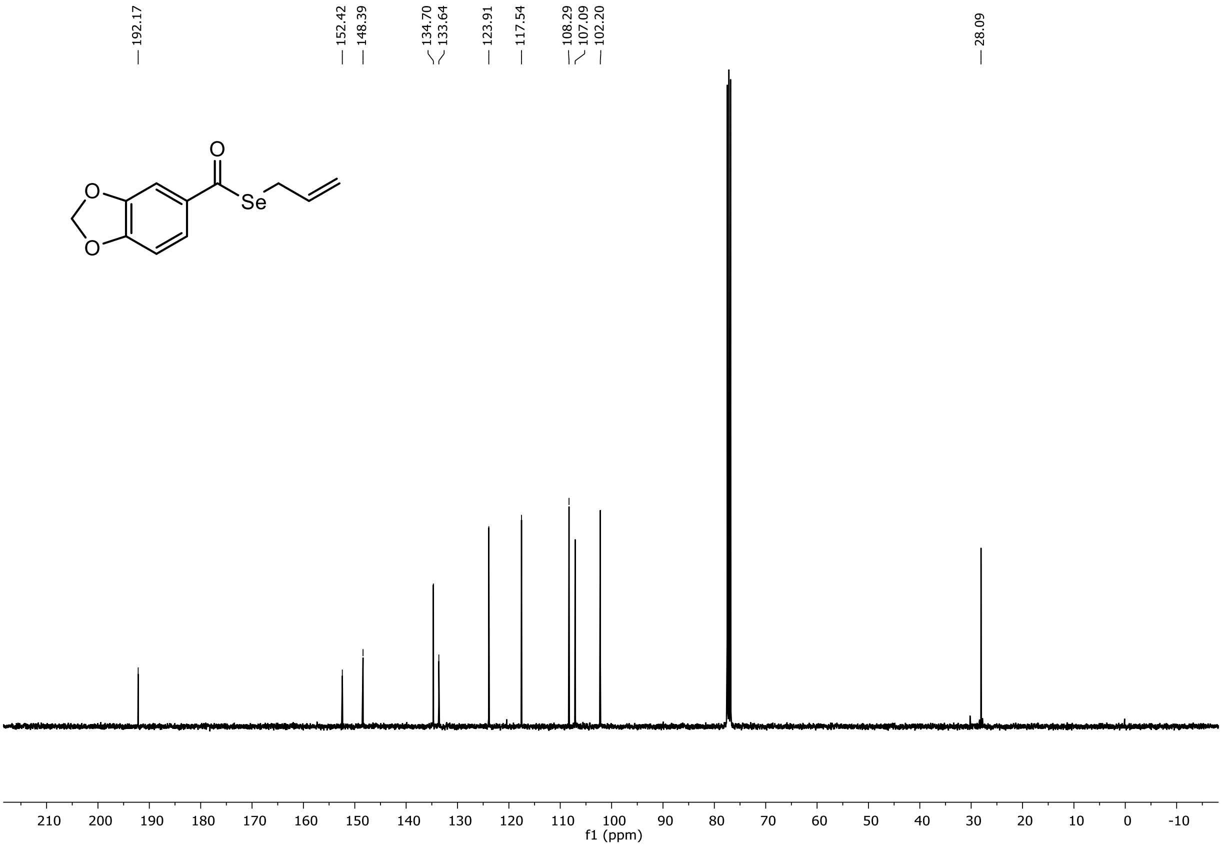

Figure S15. ^{13}C-NMR spectrum of compound **4a.**

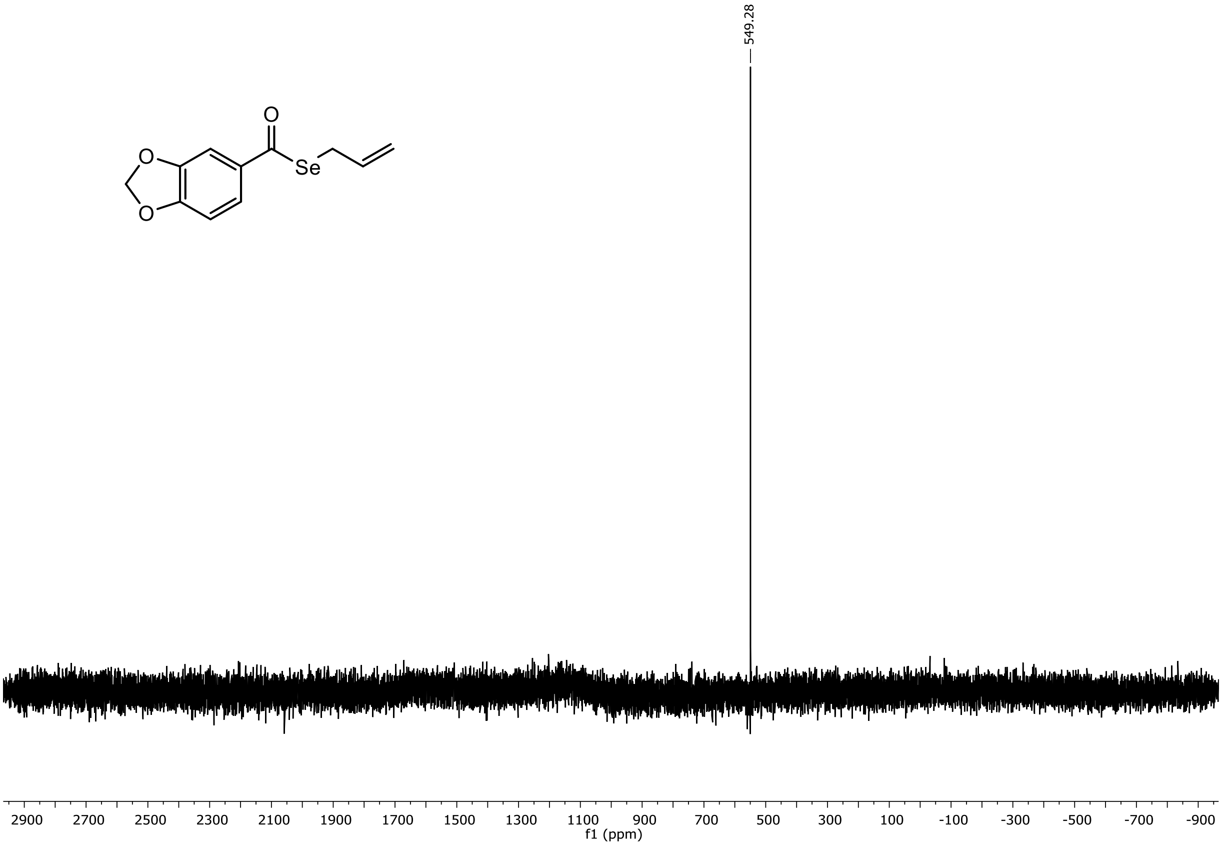

Figure S16. ^{77}Se-NMR spectrum of compound **4a.**

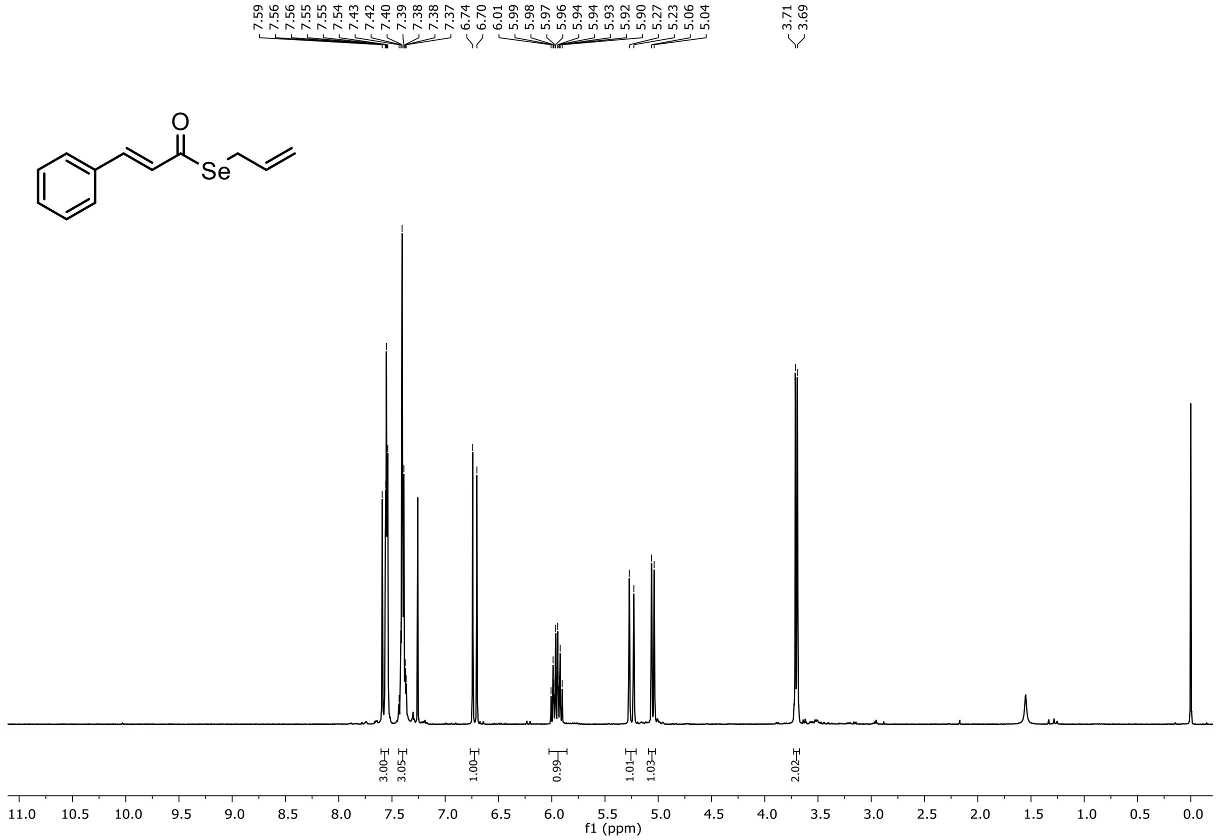

Figure S17. ¹H-NMR spectrum of compound **5a**.

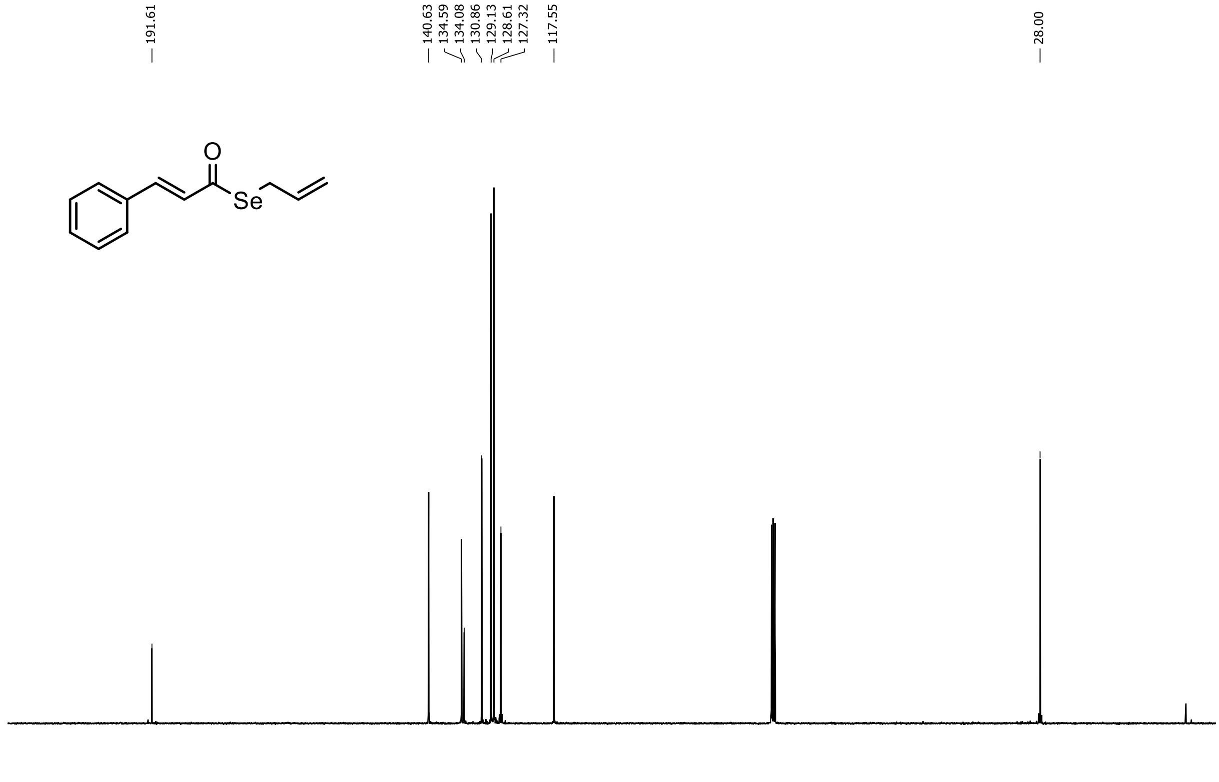

Figure S18. ¹³C-NMR spectrum of compound **5a.**

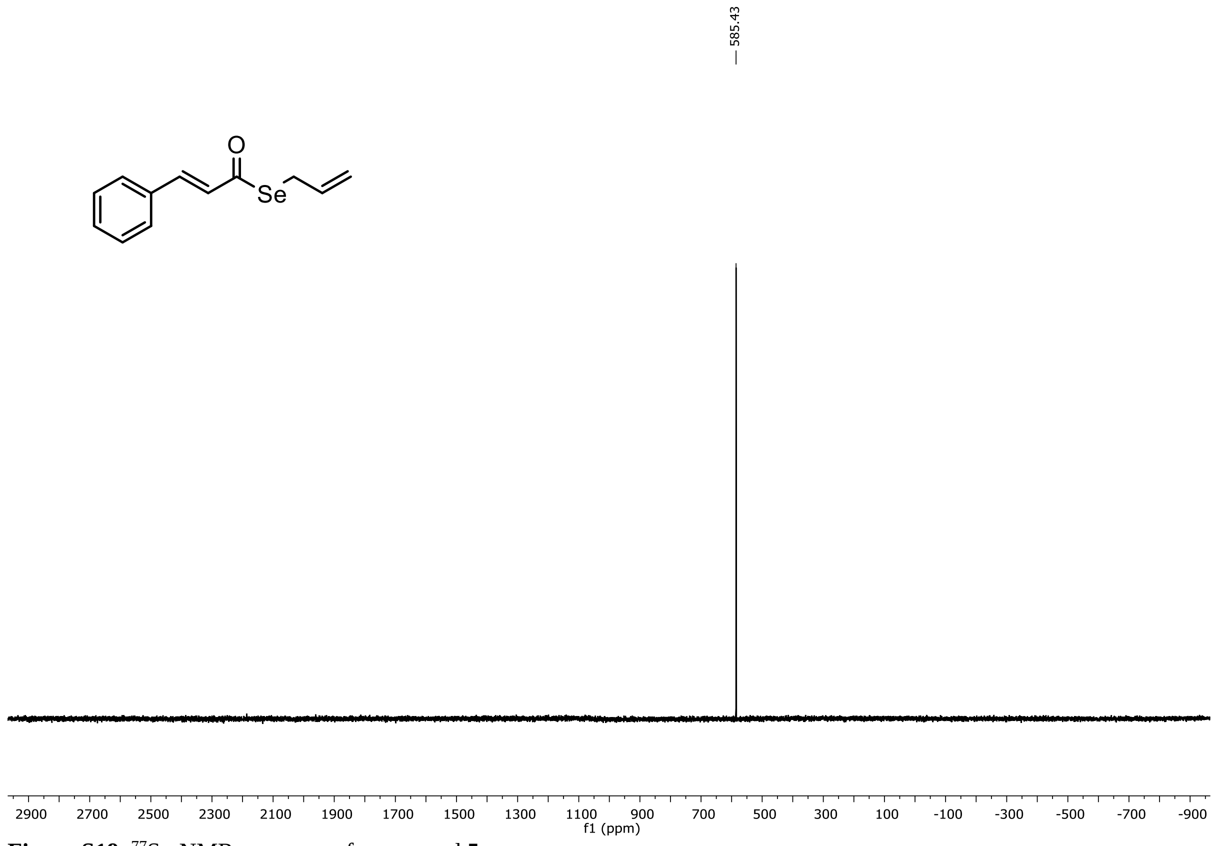

Figure S19. ^{77}Se-NMR spectrum of compound **5a.**

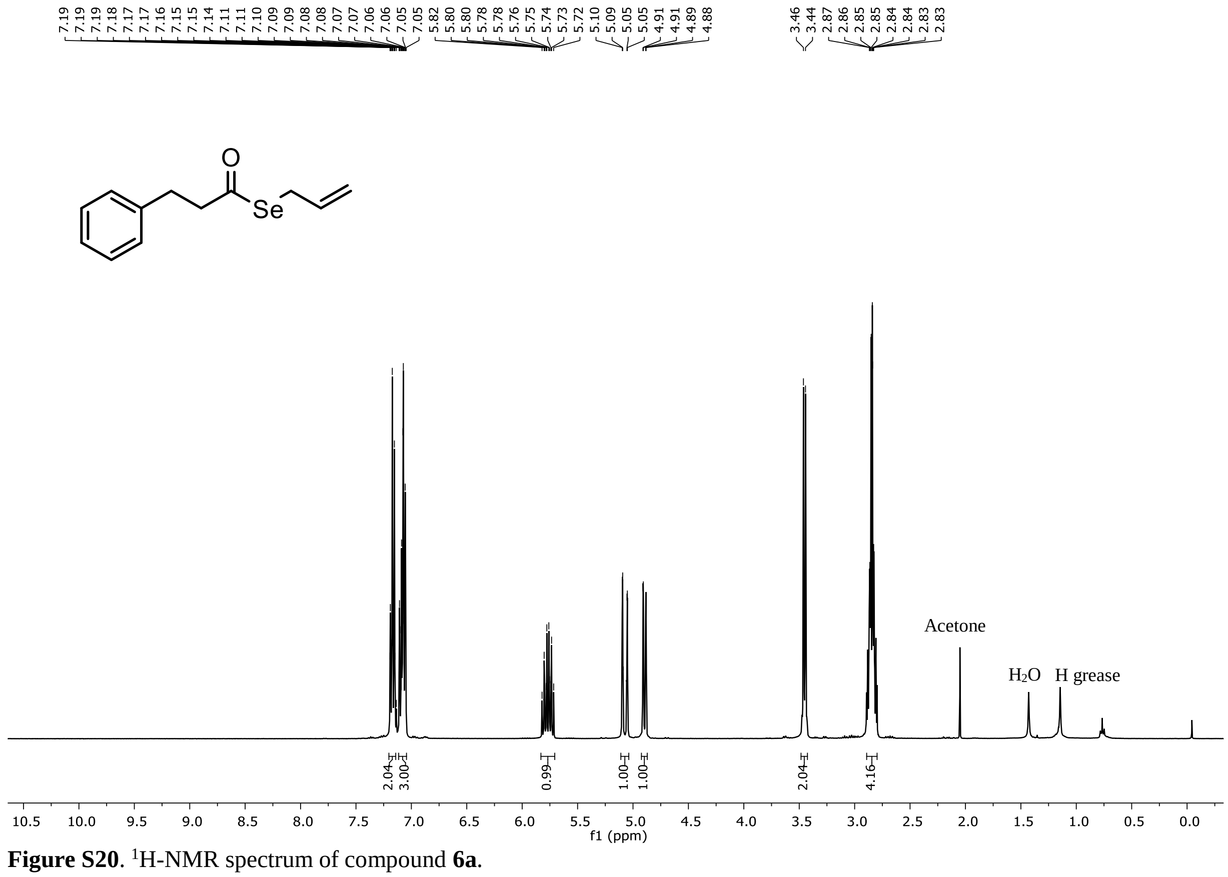

Figure S20. ^{1}H-NMR spectrum of compound **6a**.

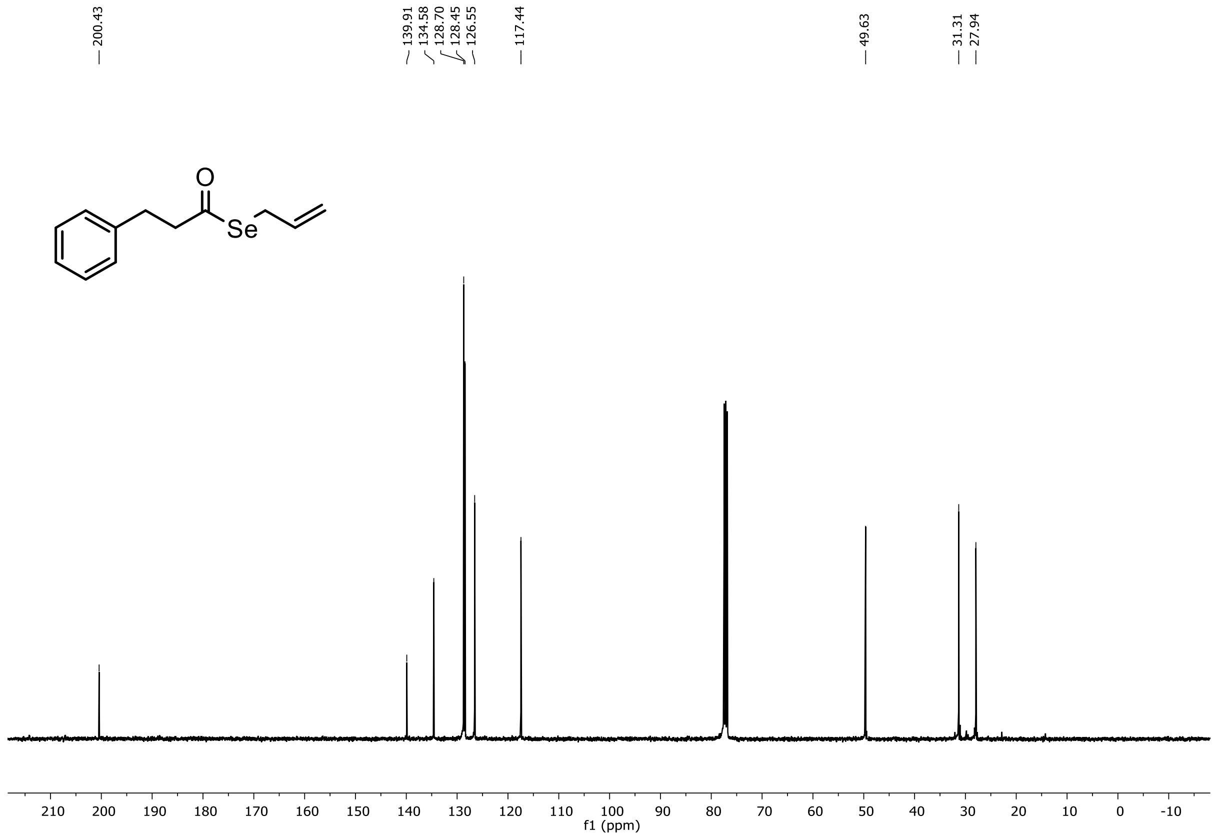

Figure S21. ^{13}C-NMR spectrum of compound **6a.**

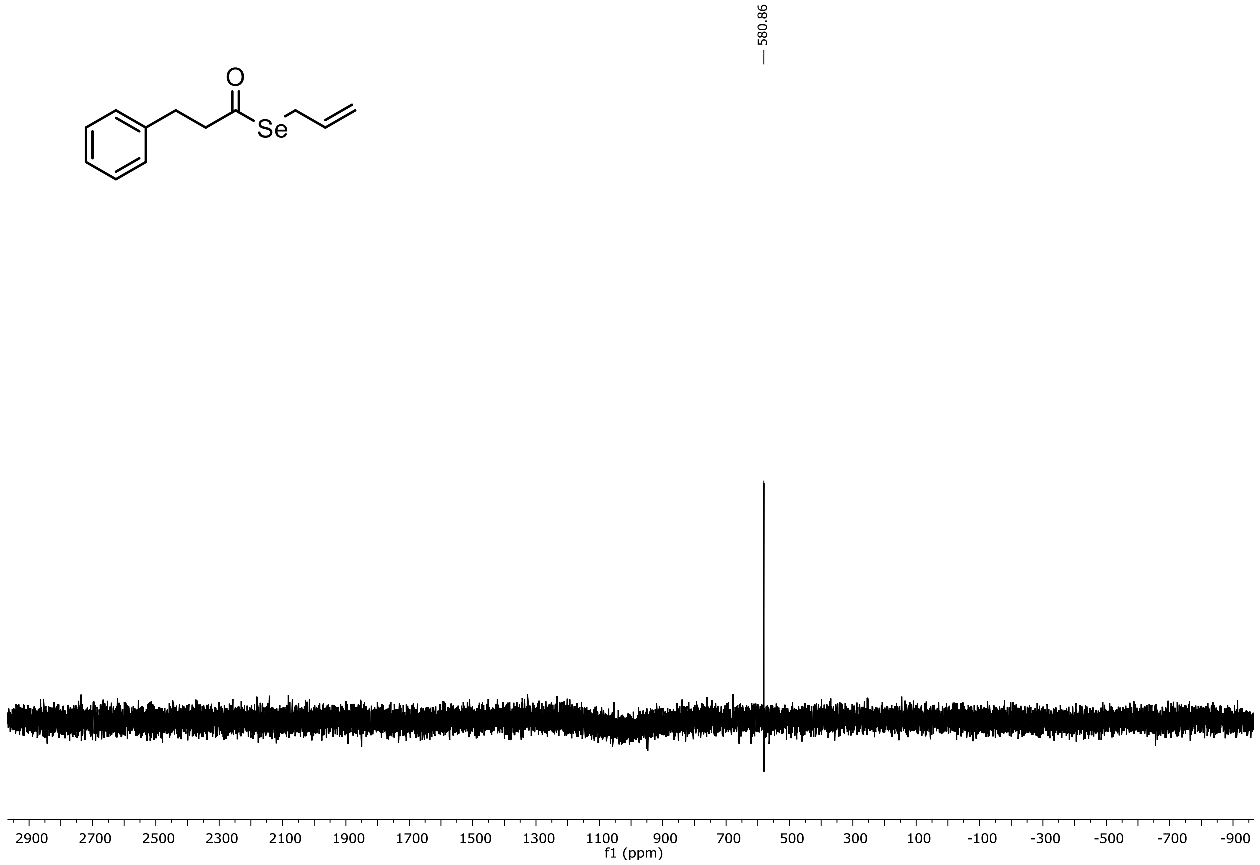

Figure S22. ^{77}Se-NMR spectrum of compound **6a.**

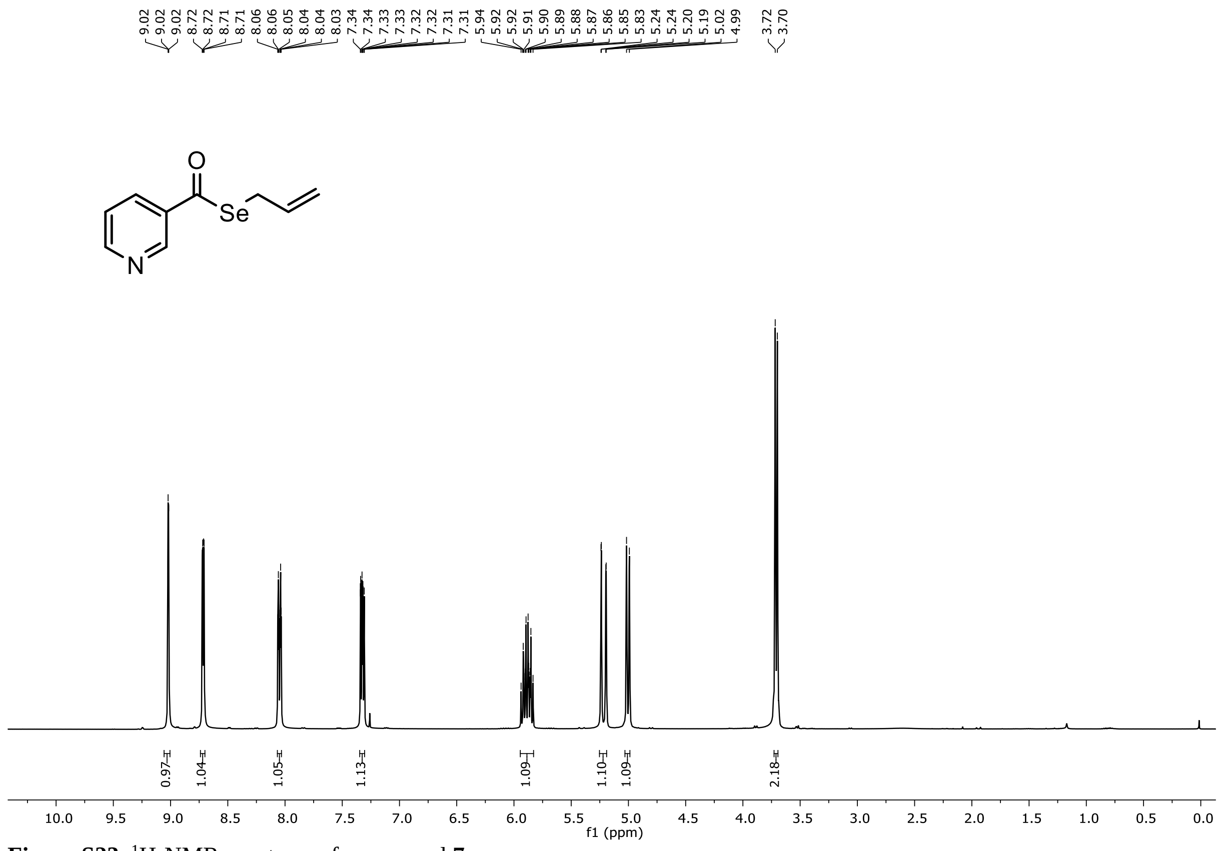

Figure S23. ^{1}H-NMR spectrum of compound **7a**.

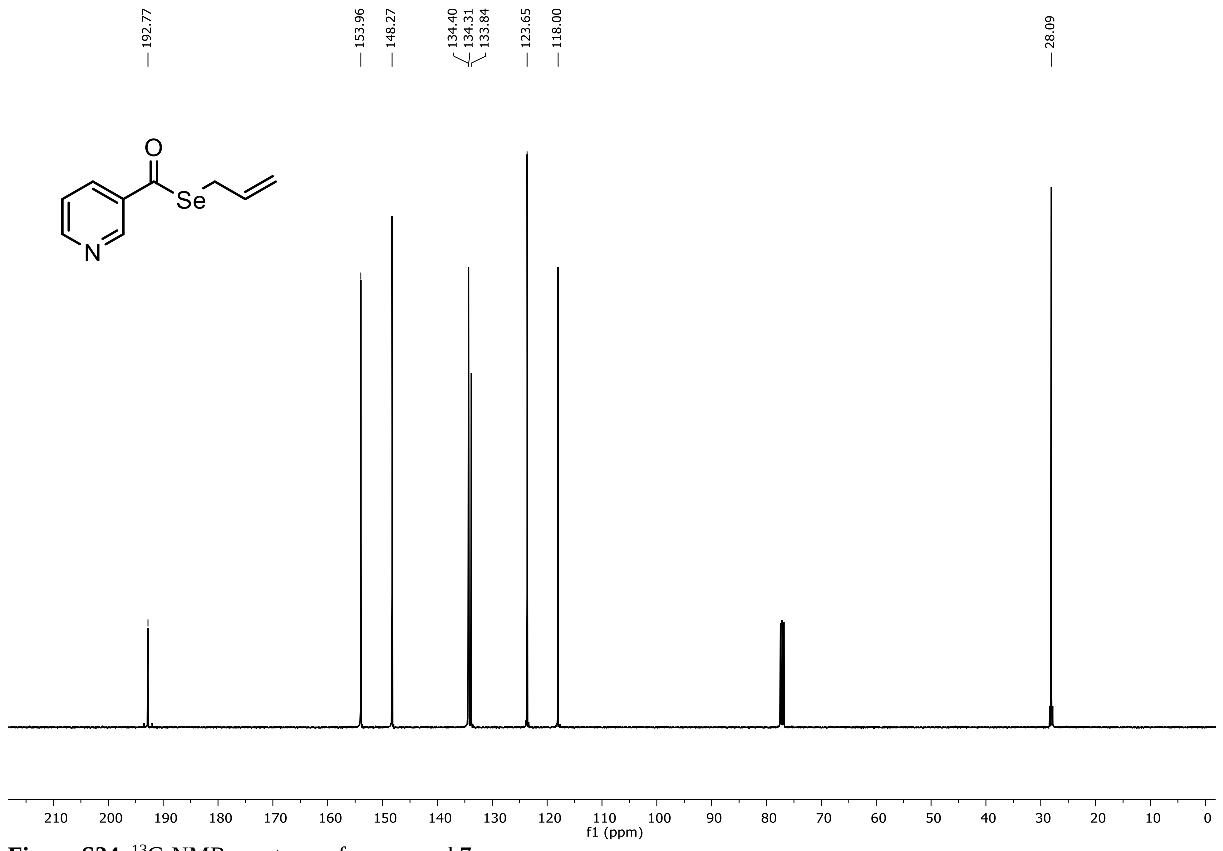

Figure S24. ^{13}C-NMR spectrum of compound **7a.**

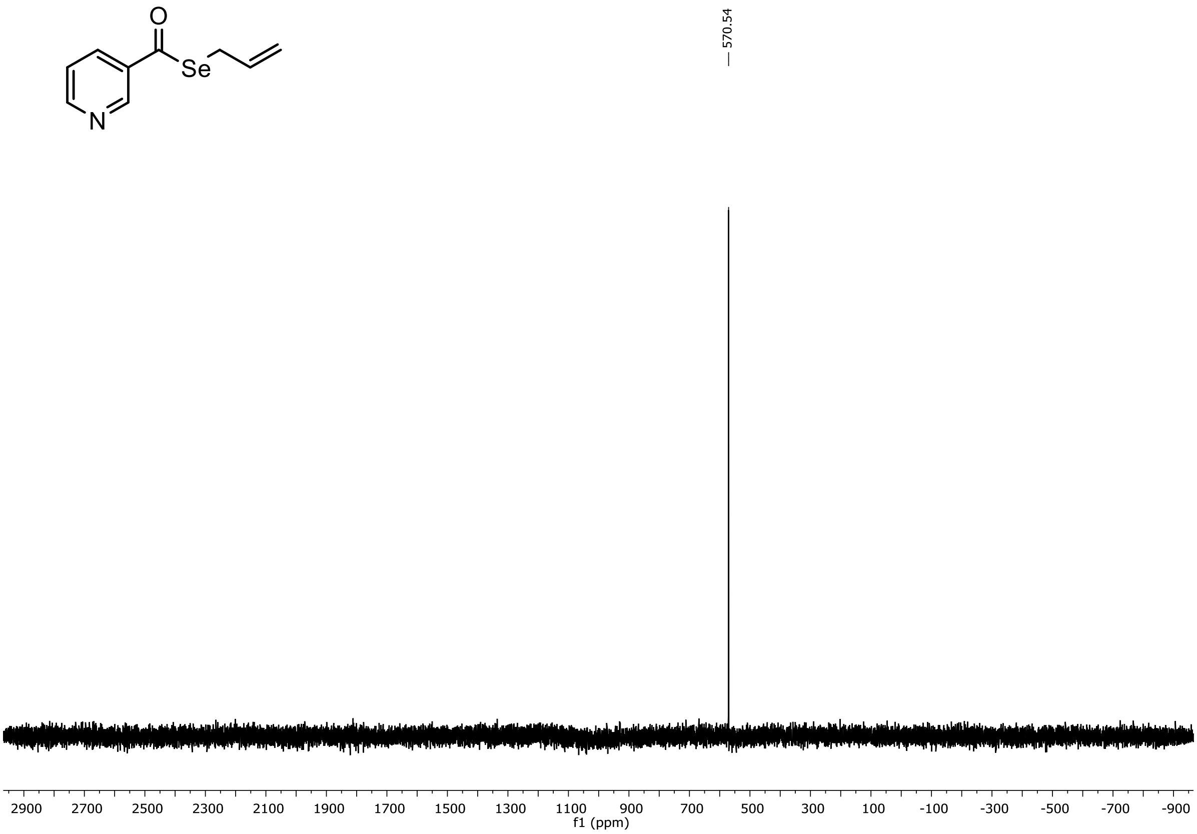

Figure S25. ^{77}Se-NMR spectrum of compound **7a.**

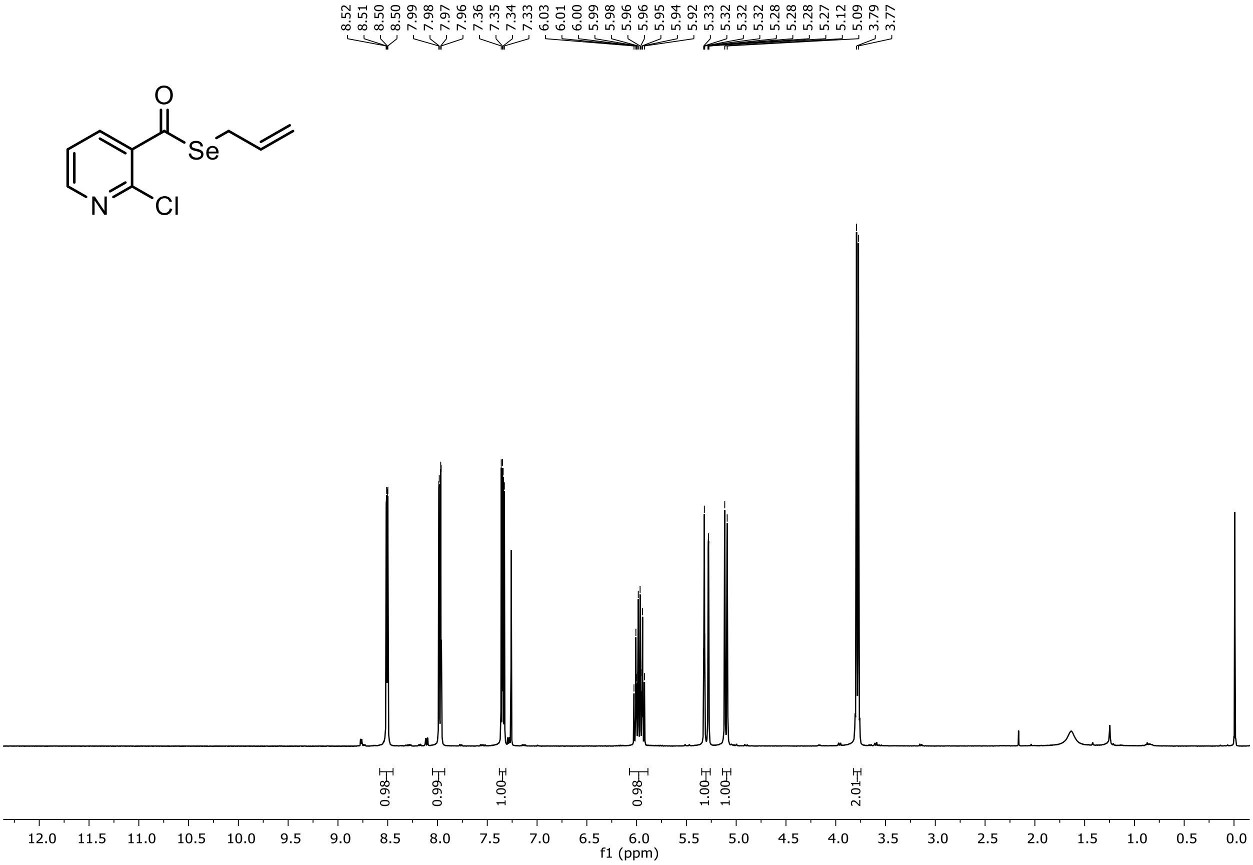

Figure S26. ^{1}H-NMR spectrum of compound **8a**.

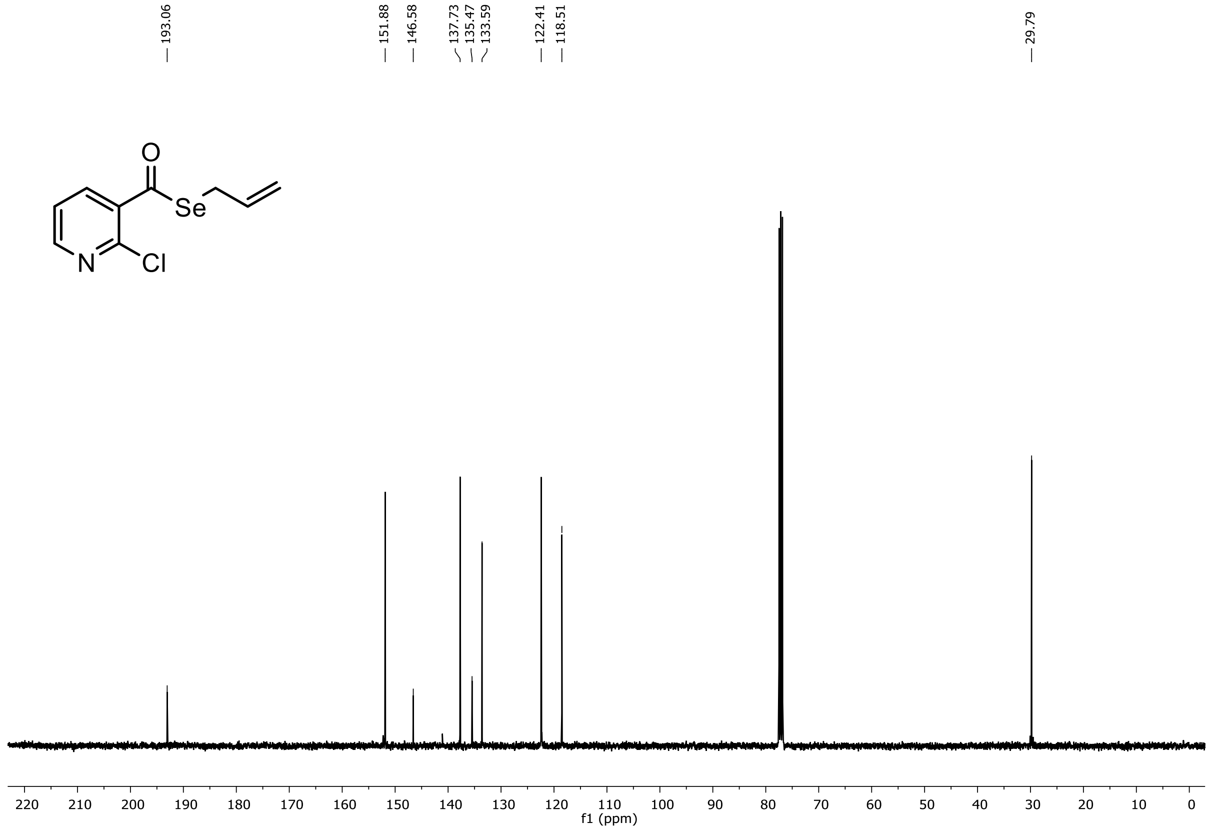

Figure S27. ^{13}C-NMR spectrum of compound **8a.**

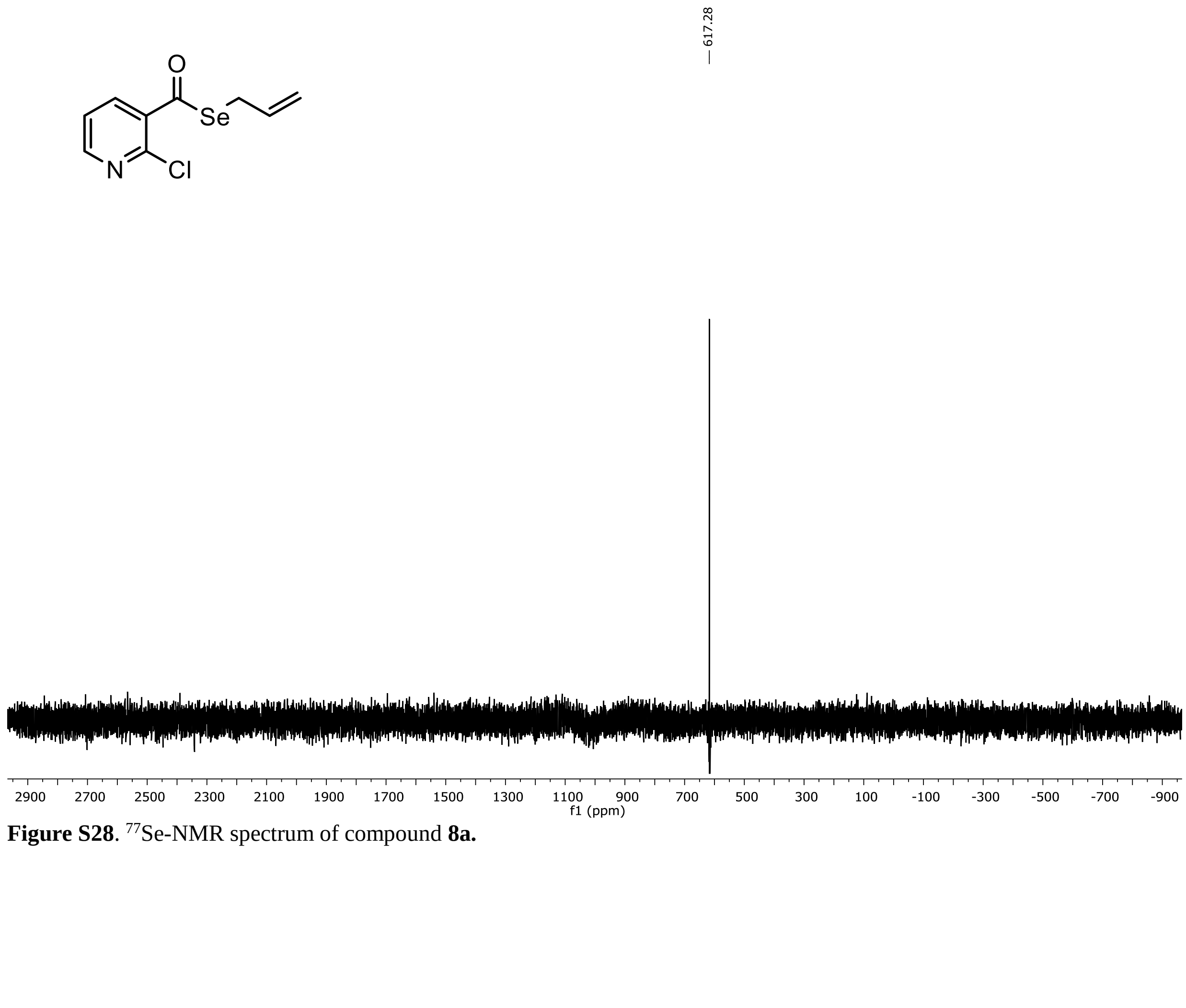

Figure S28. ^{77}Se-NMR spectrum of compound **8a.**

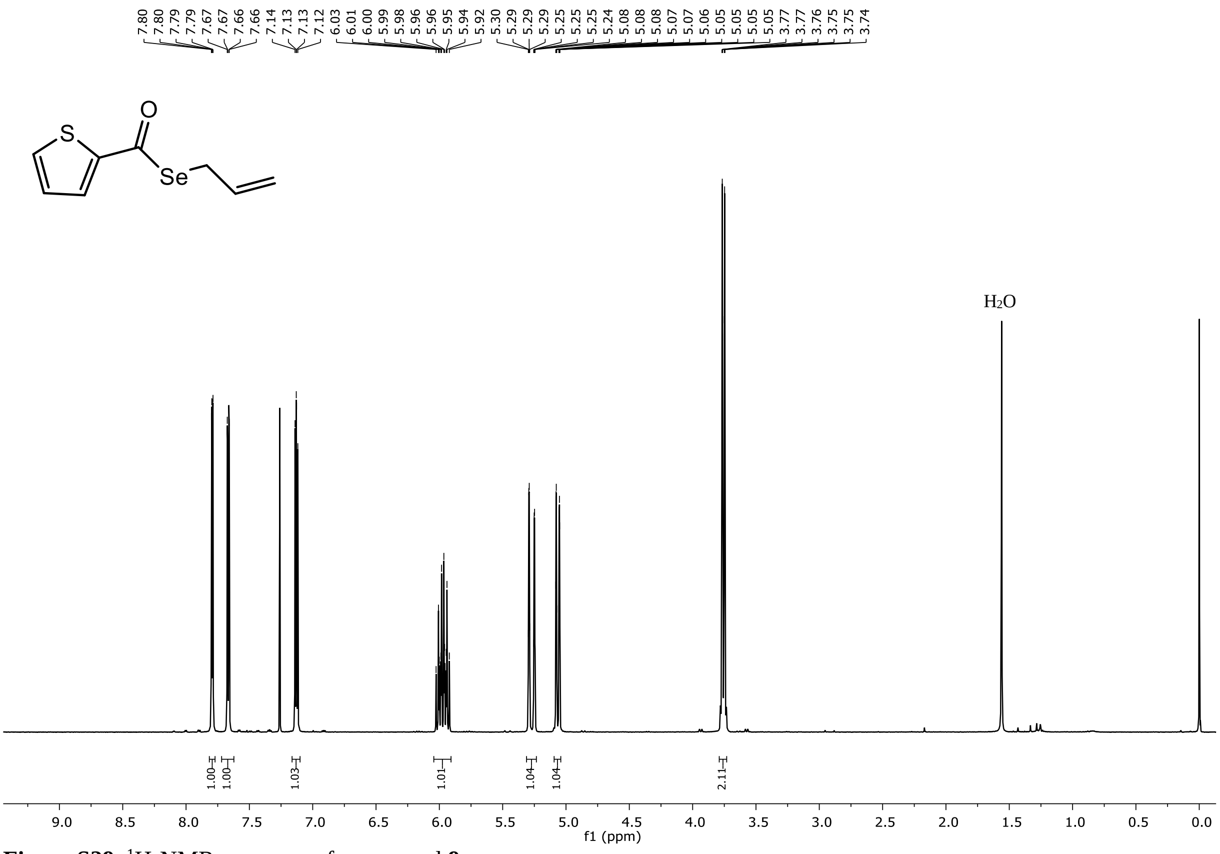

Figure S29. ^{1}H-NMR spectrum of compound **9a**.

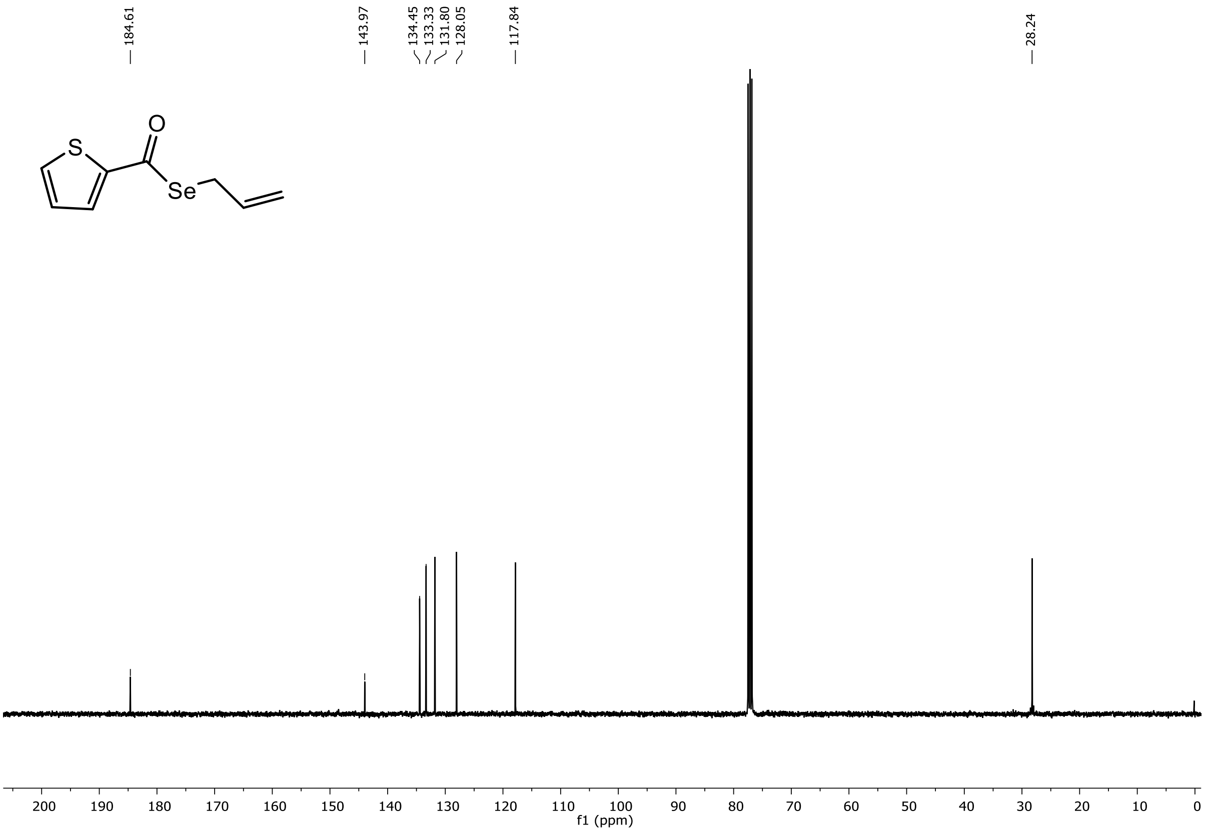

Figure S30. ^{13}C-NMR spectrum of compound **9a.**

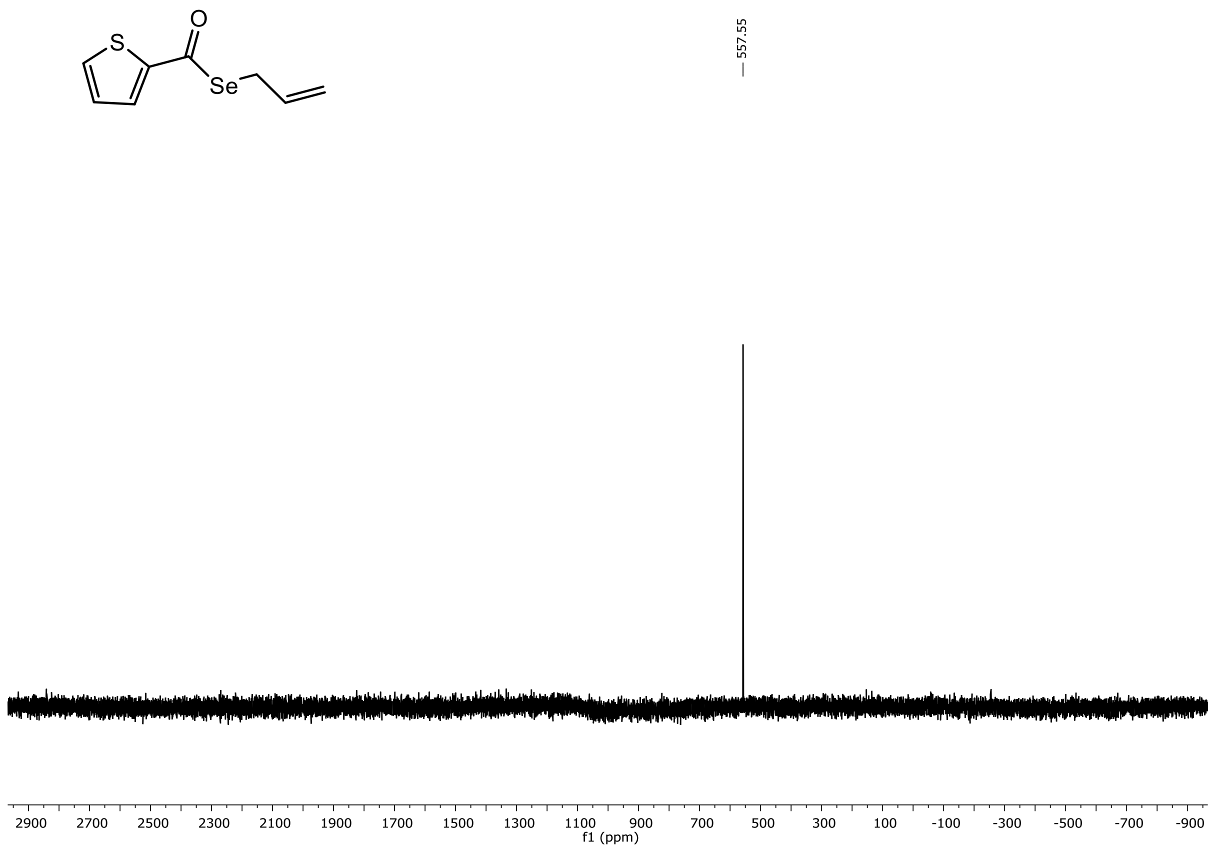

Figure S31. ^{77}Se-NMR spectrum of compound **9a.**

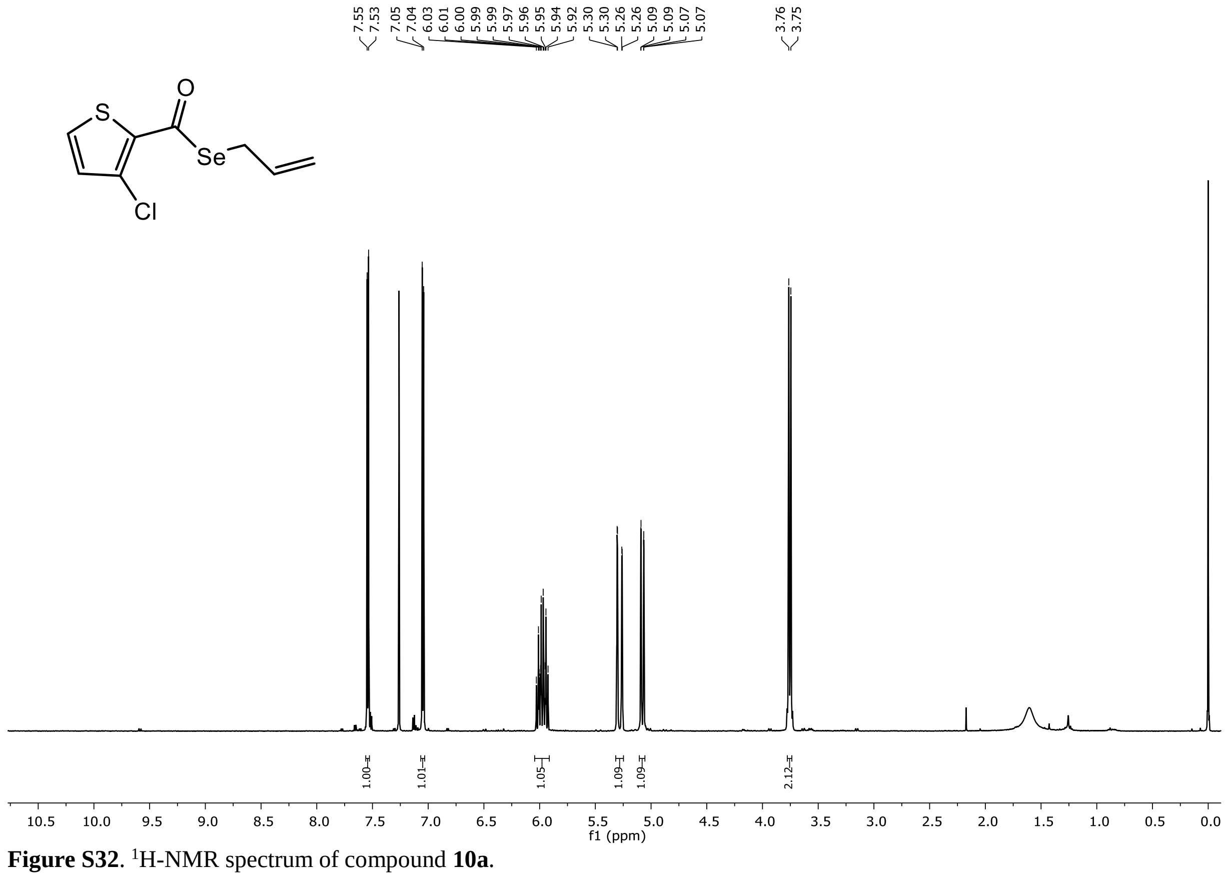

Figure S32. ^{1}H-NMR spectrum of compound **10a**.

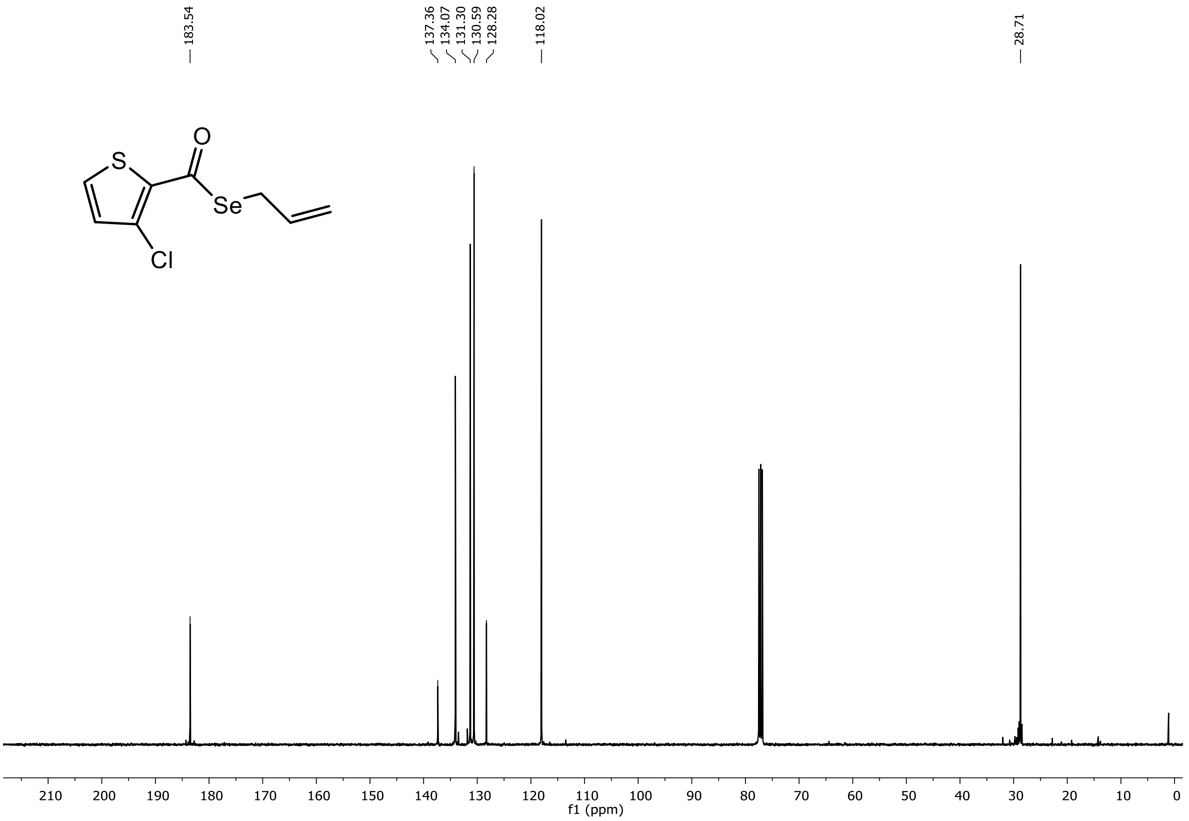

Figure S33. ^{13}C-NMR spectrum of compound **10a.**

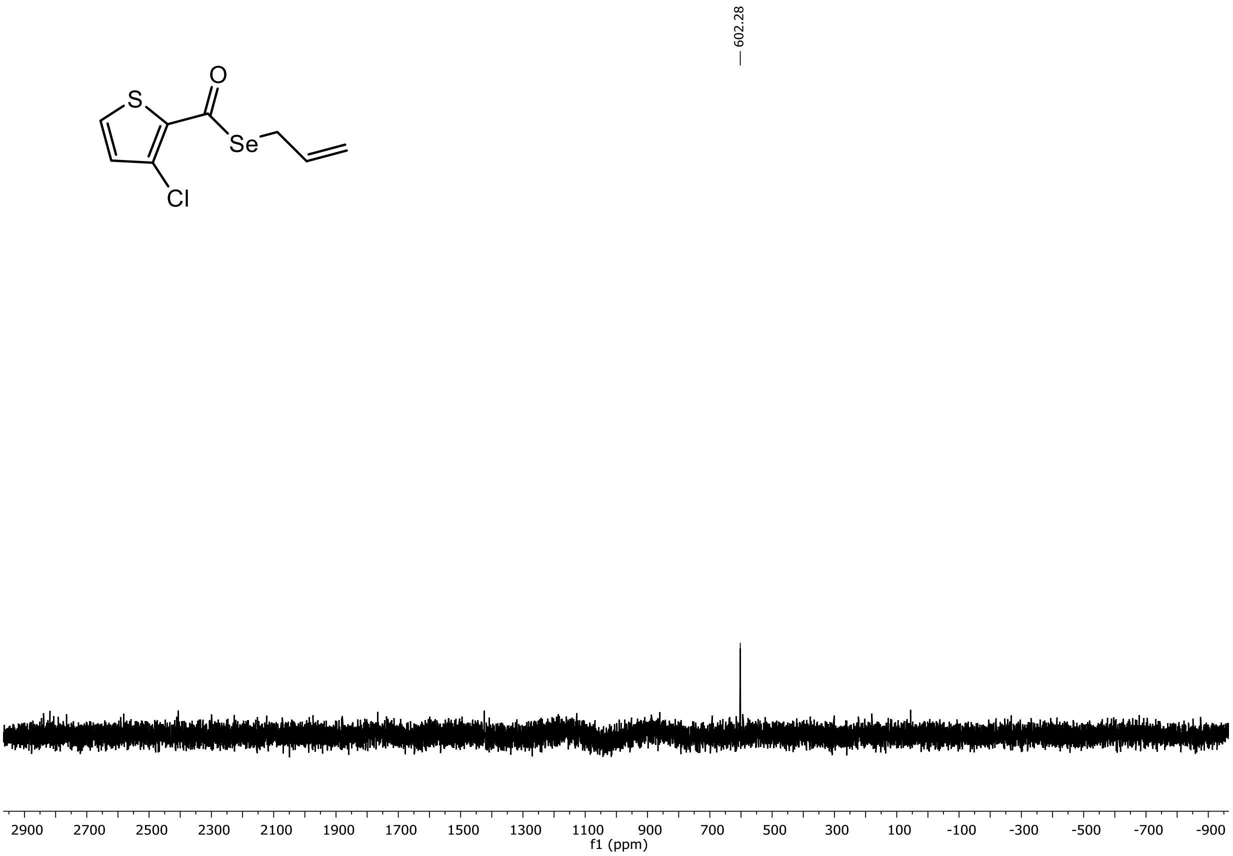

Figure S34. ^{77}Se-NMR spectrum of compound **10a.**

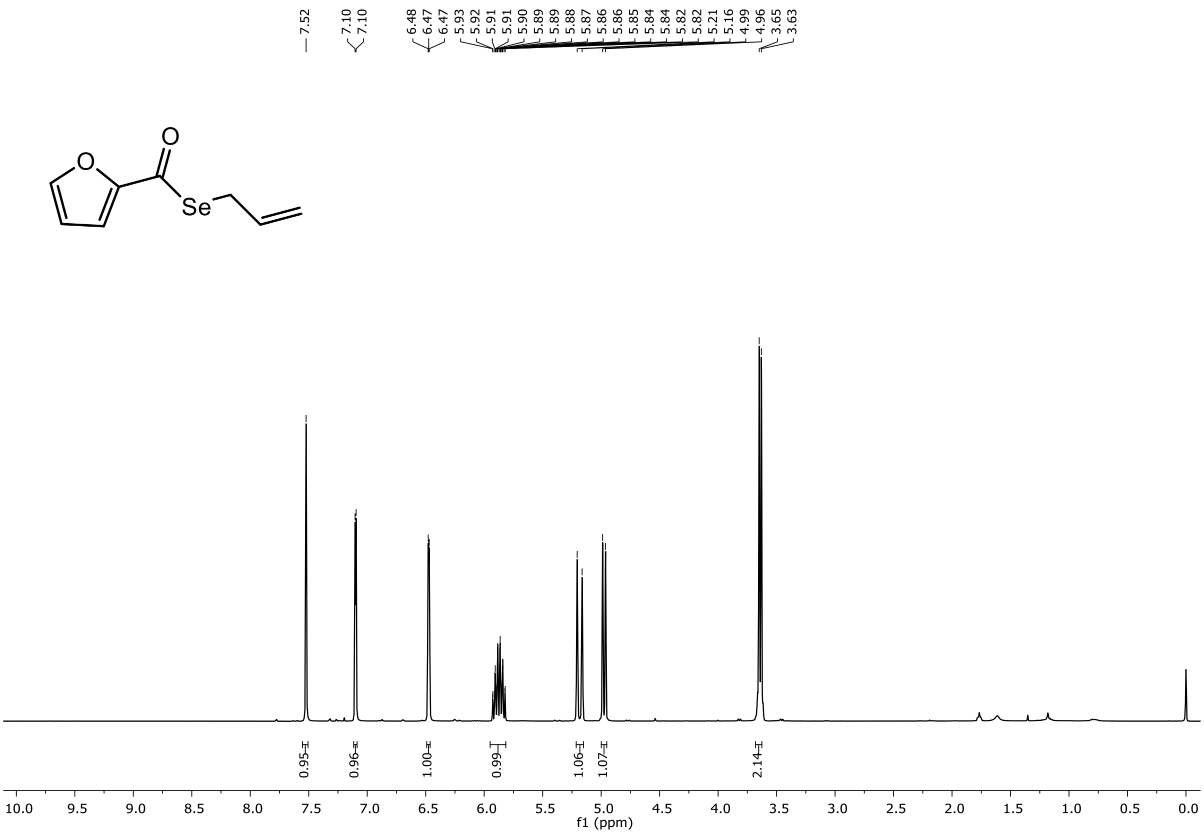

Figure S35. ^{1}H-NMR spectrum of compound **11a**.

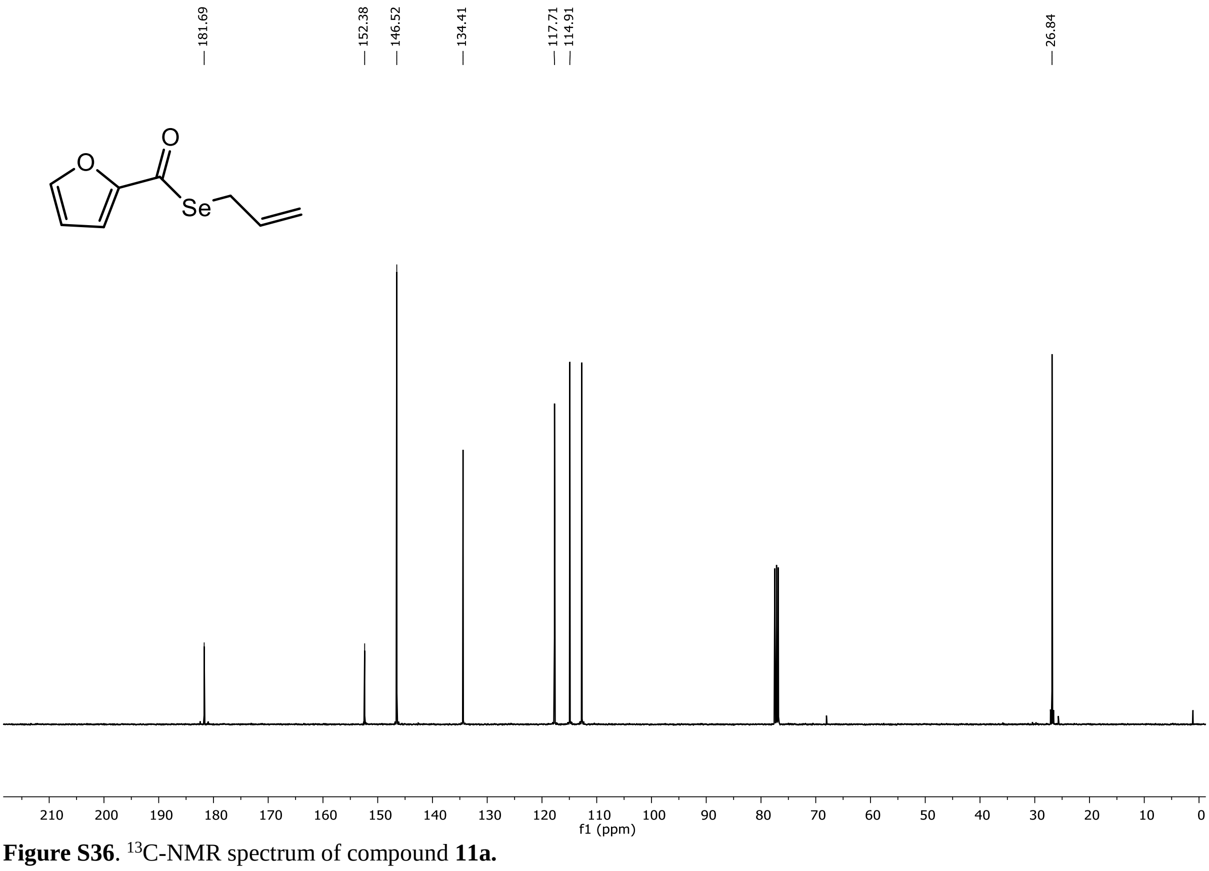

Figure S36. ^{13}C-NMR spectrum of compound **11a.**

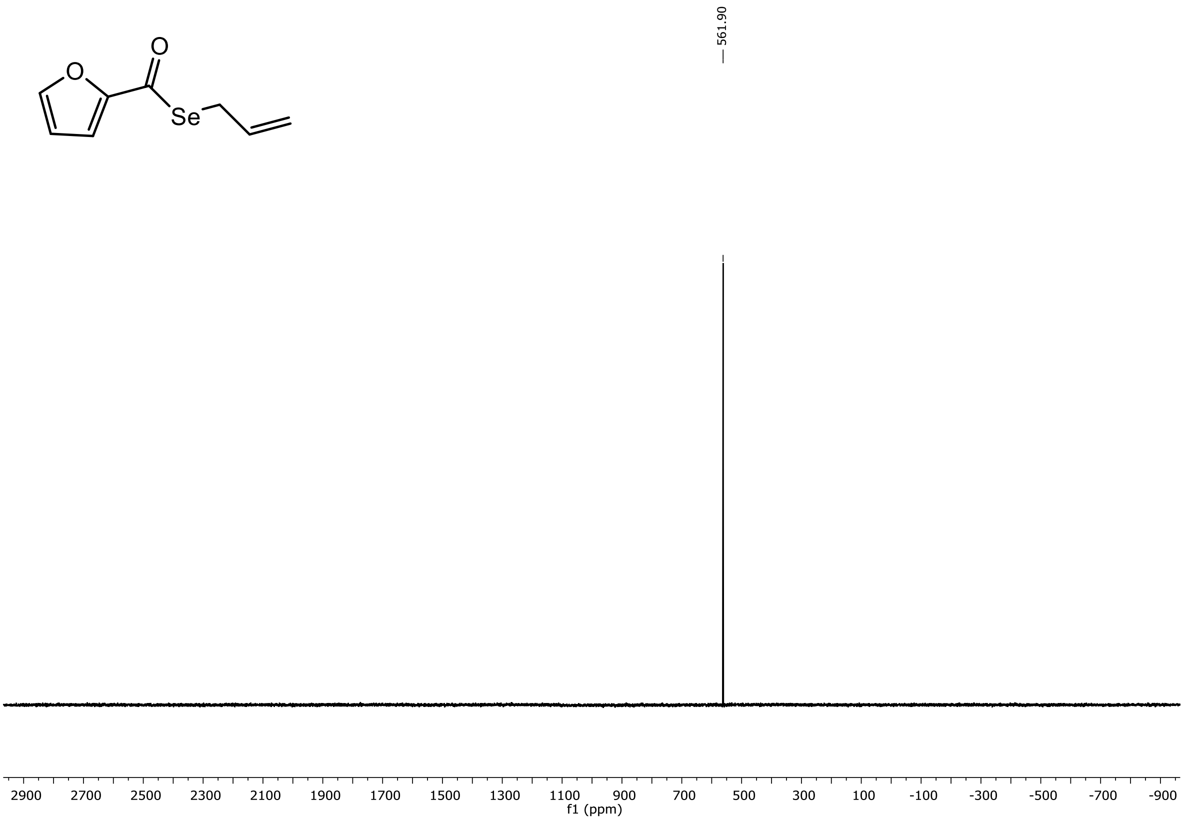

Figure S37. ^{77}Se-NMR spectrum of compound **11a.**

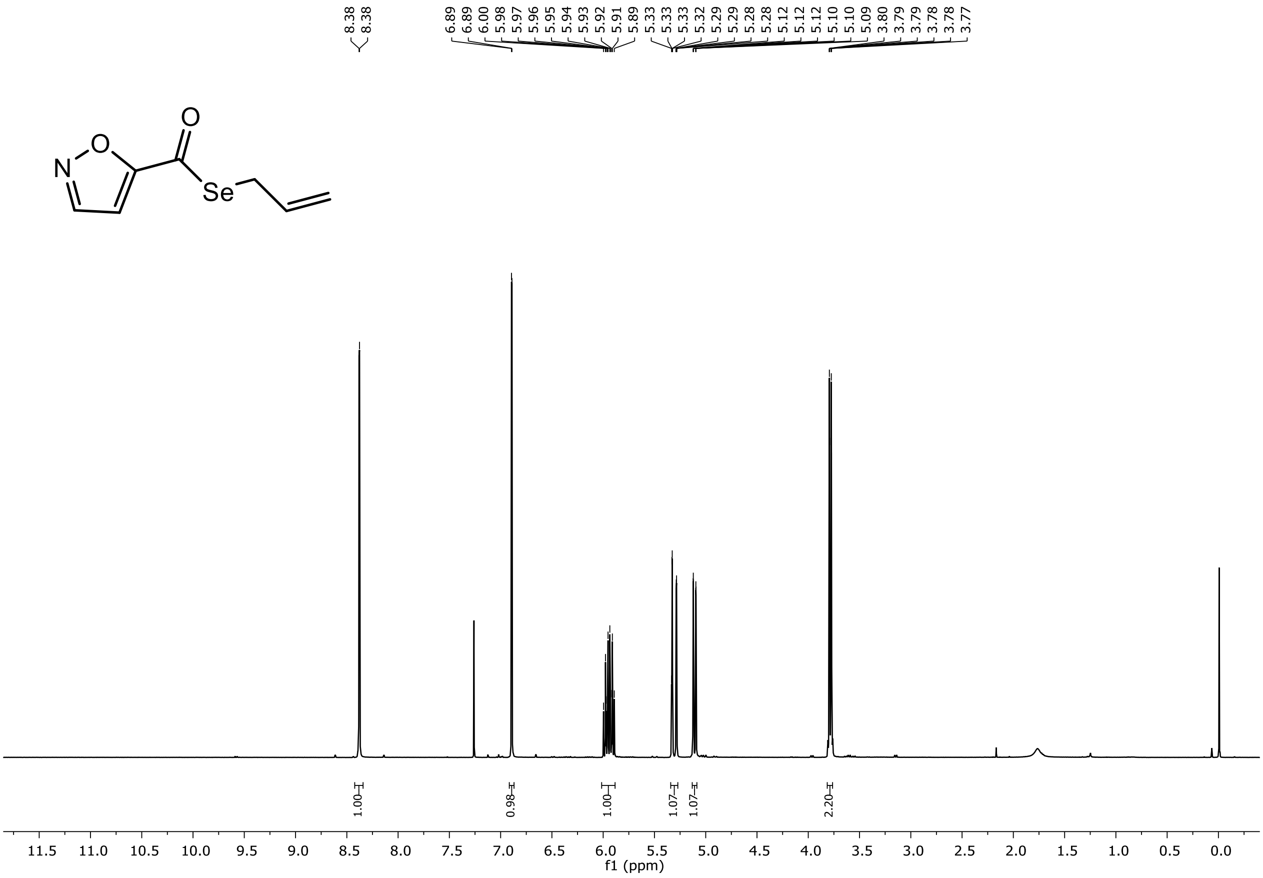

Figure S38. ^{1}H-NMR spectrum of compound **12a**.

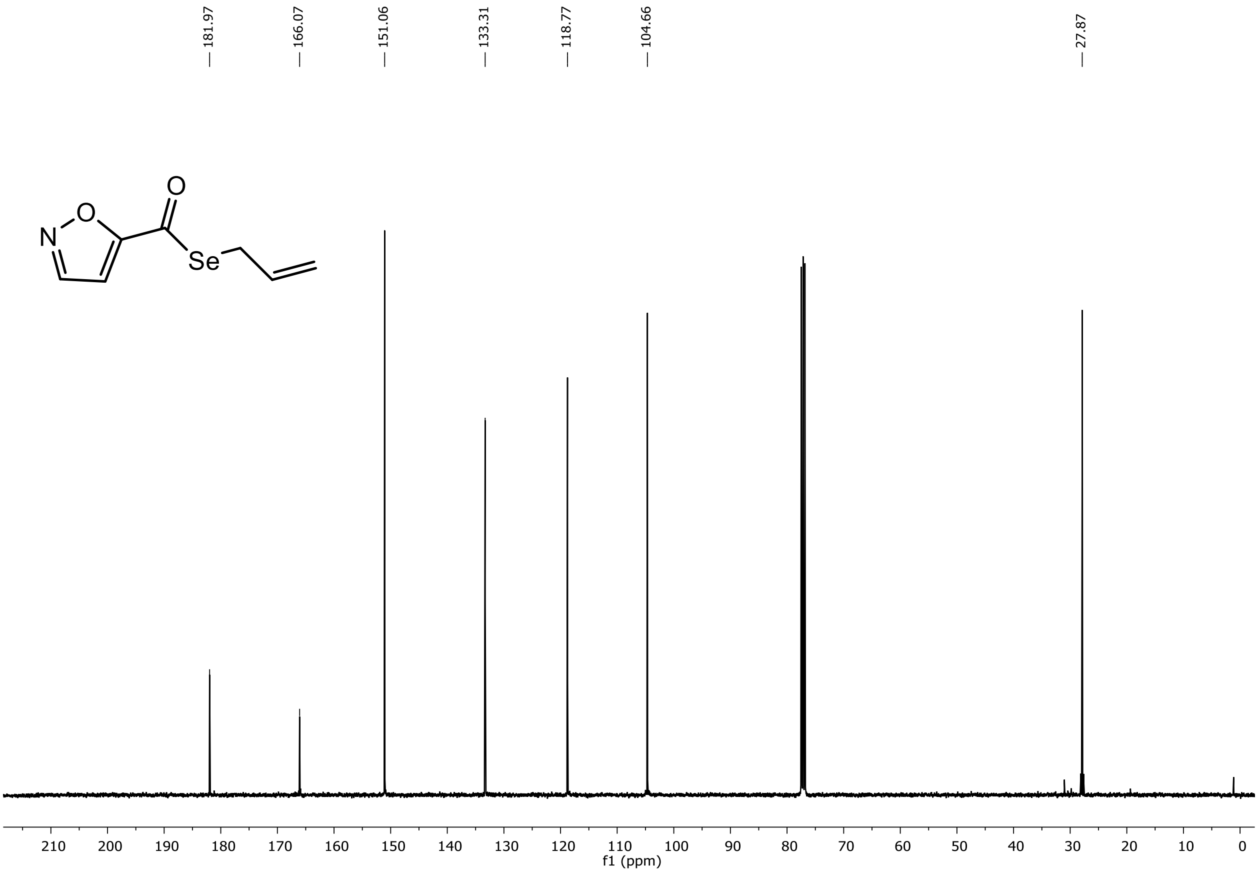

Figure S39. ^{13}C-NMR spectrum of compound **12a.**

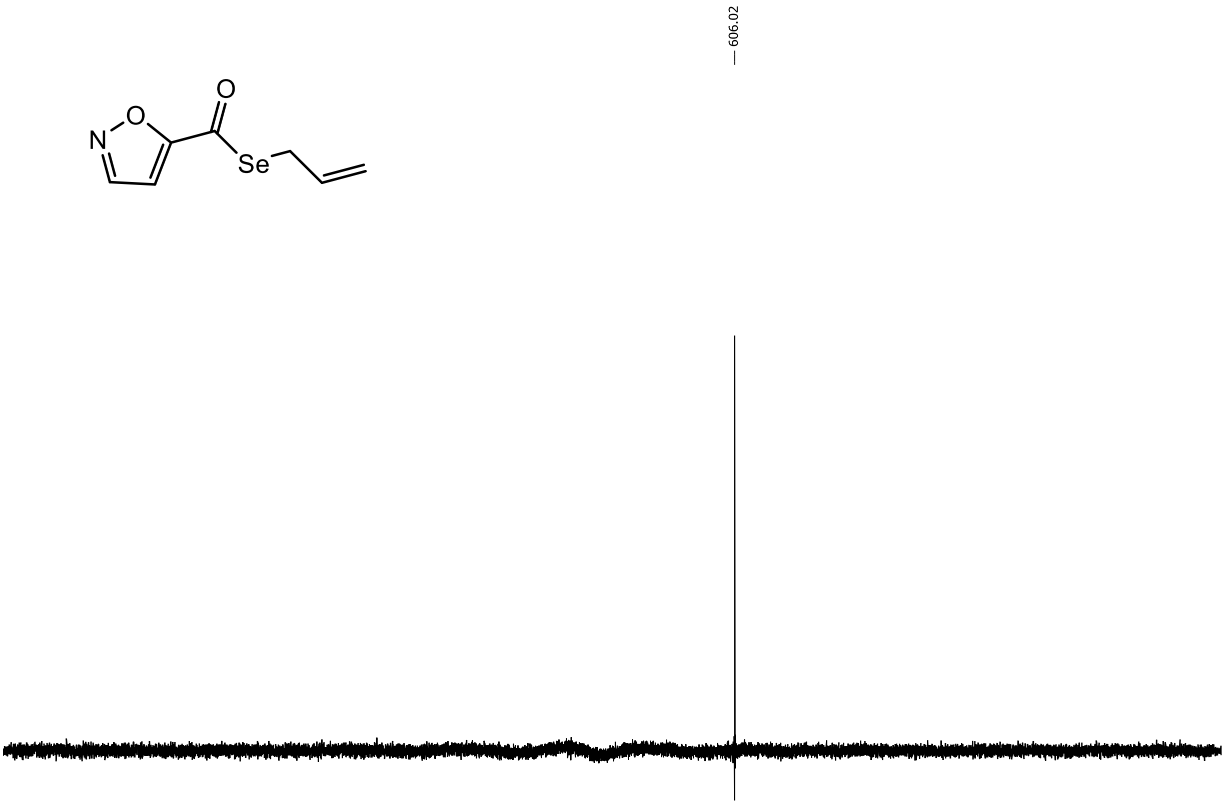

Figure S40. ^{77}Se-NMR spectrum of compound **12a.**

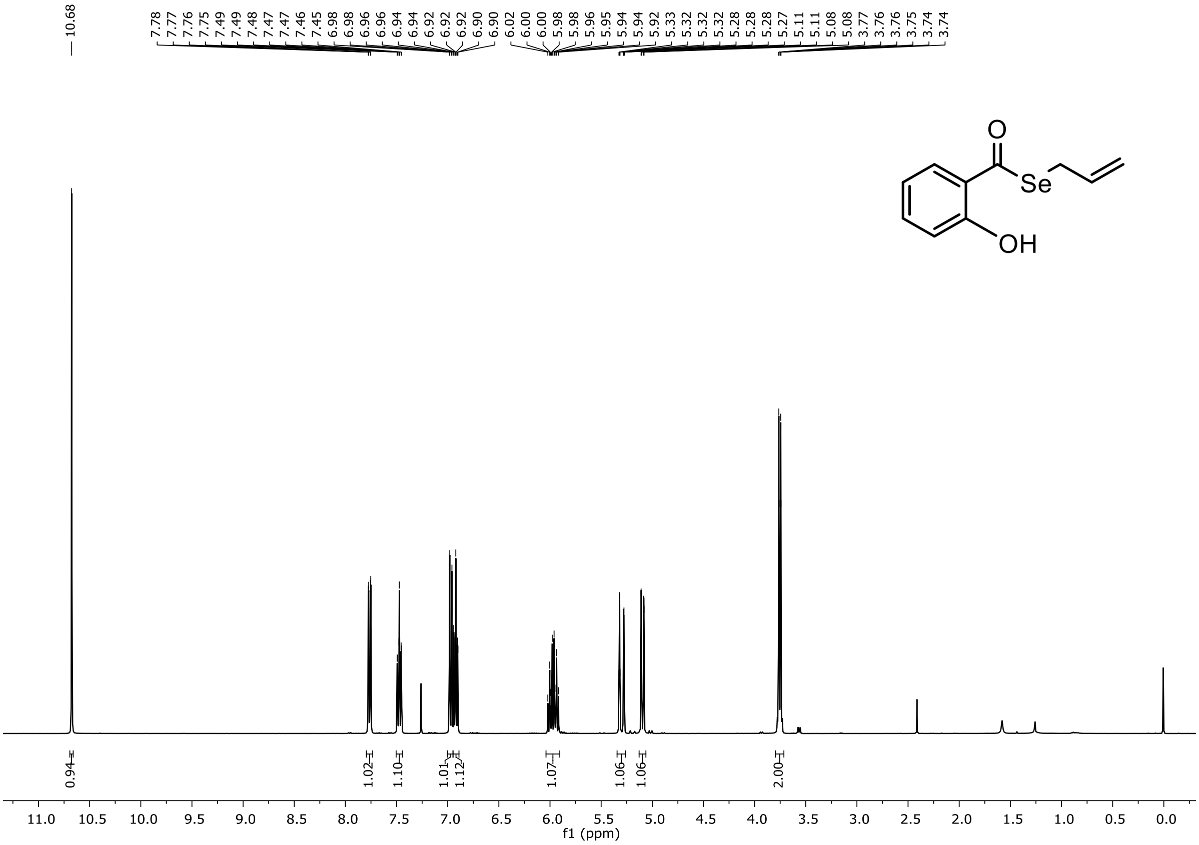

Figure S41. ^{1}H-NMR spectrum of compound **13a**.

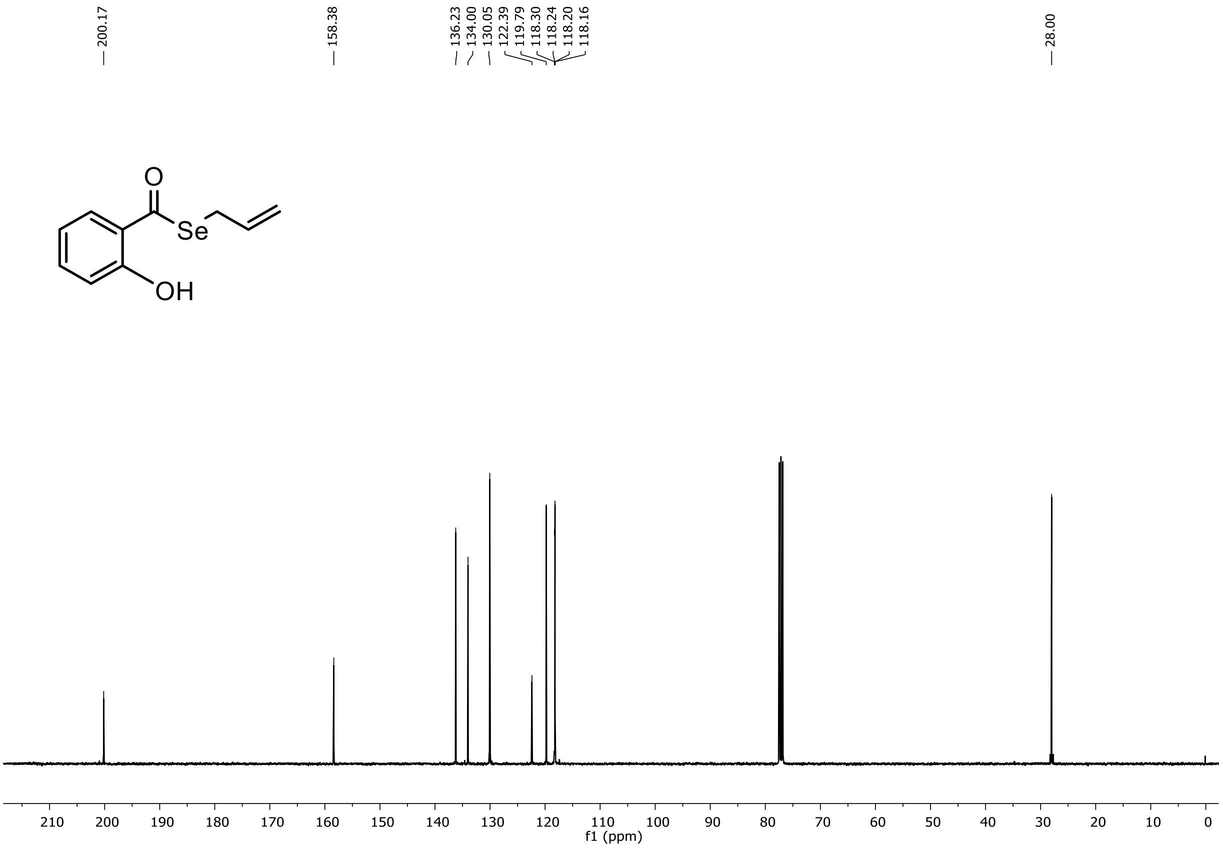

Figure S42. ^{13}C-NMR spectrum of compound **13a.**

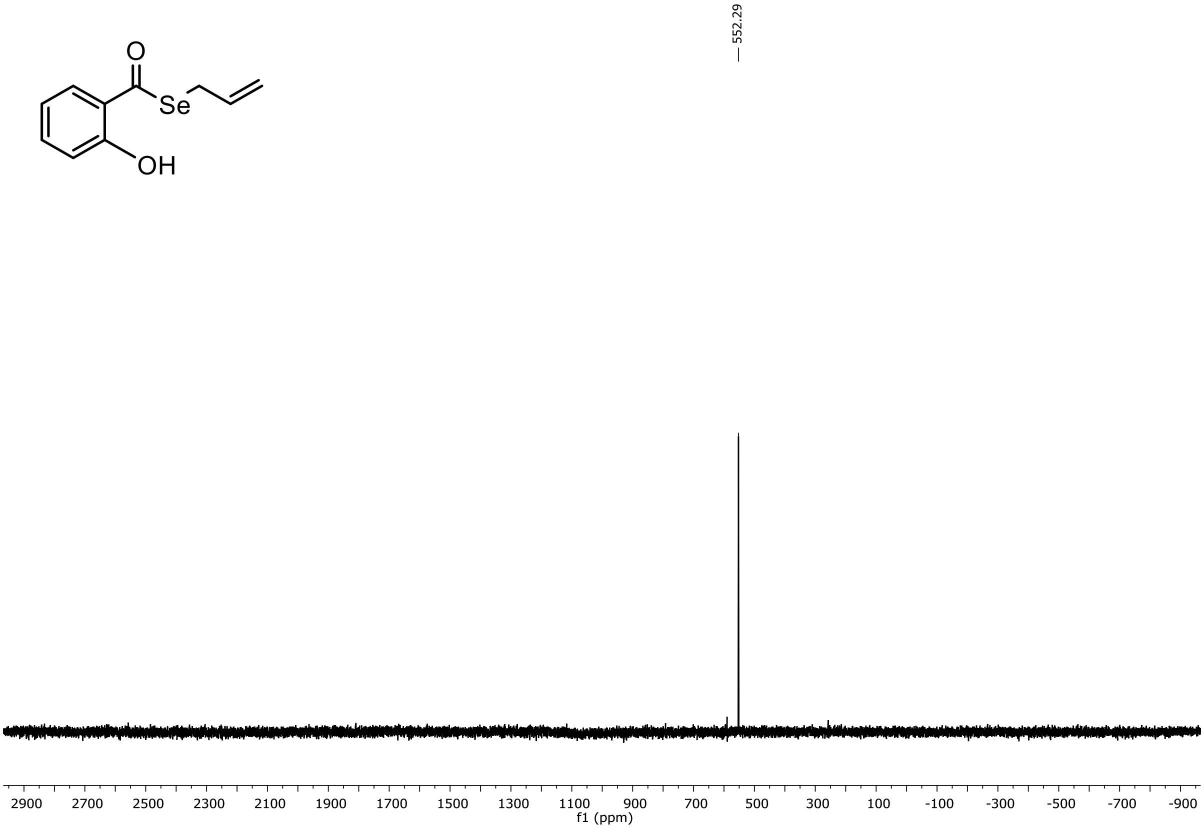

Figure S43. ^{77}Se-NMR spectrum of compound **13a.**

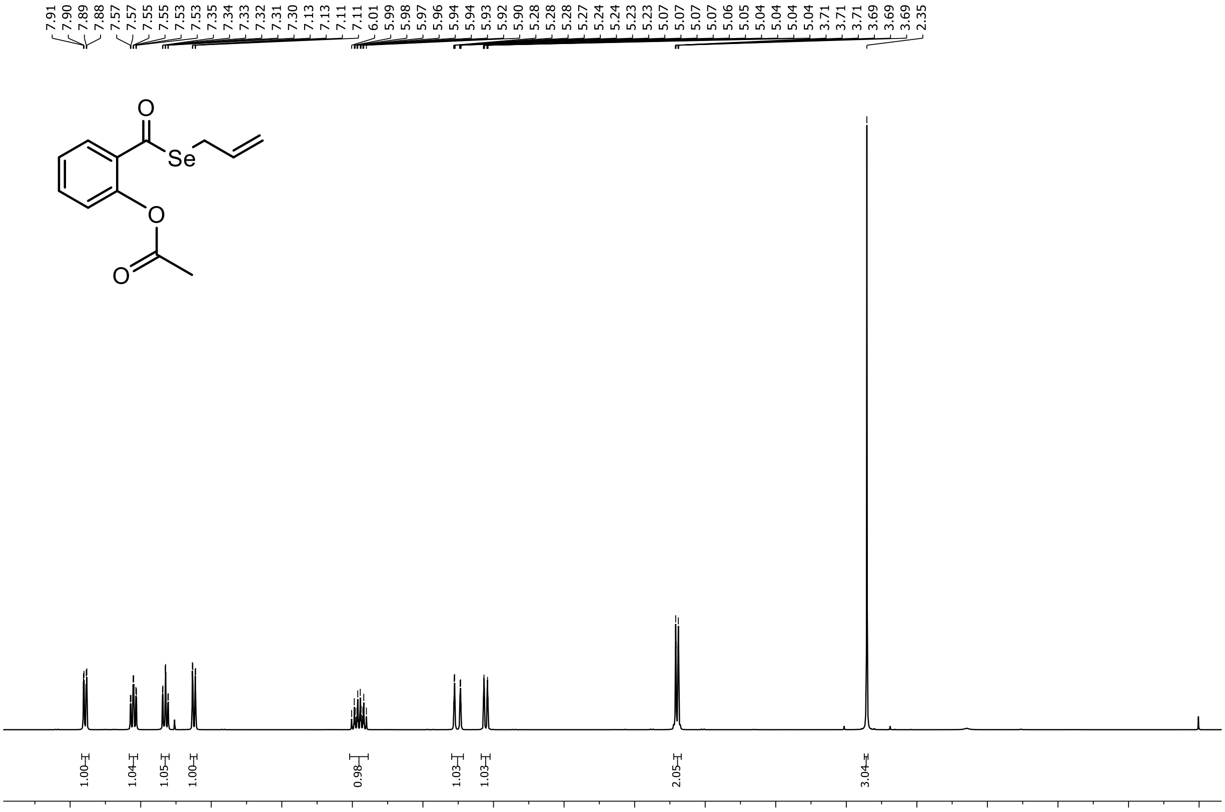

Figure S44. ^{1}H-NMR spectrum of compound **14a**.

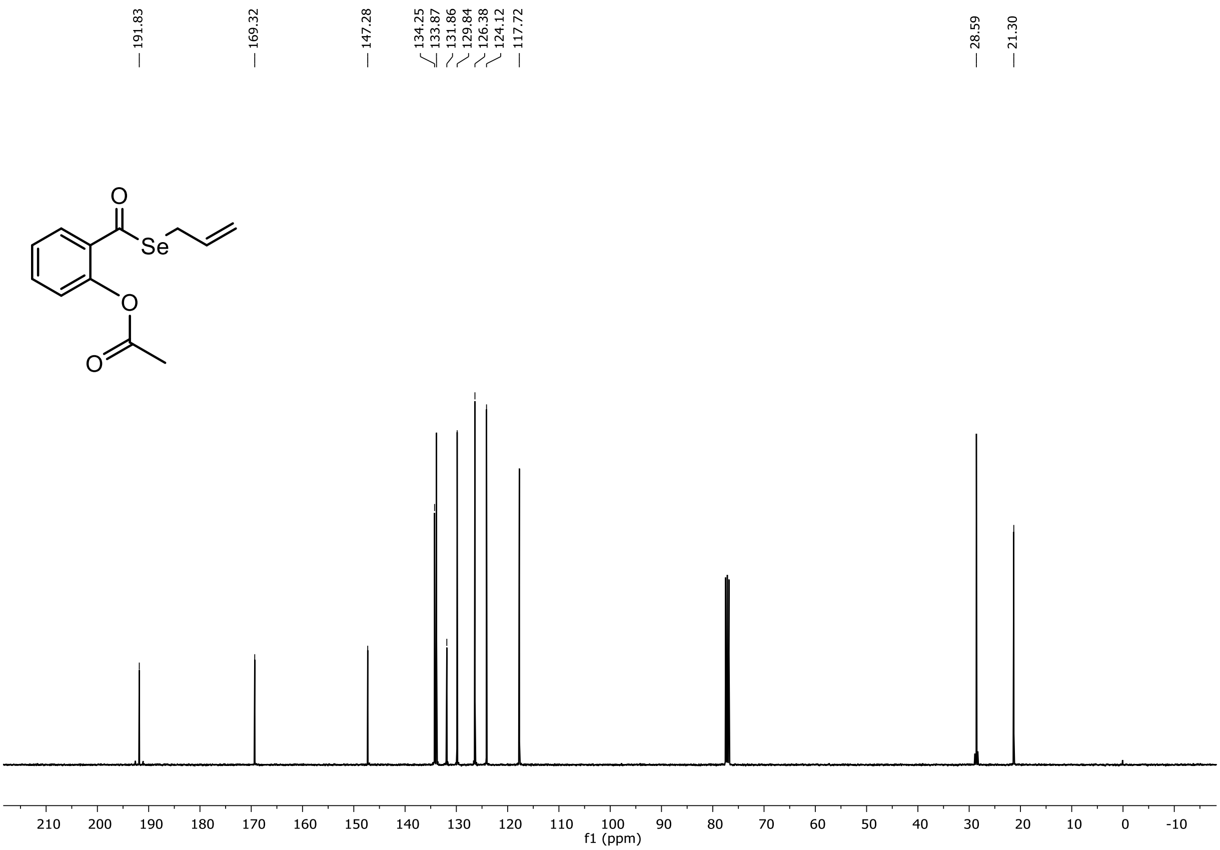

Figure S45. ^{13}C-NMR spectrum of compound **14a.**

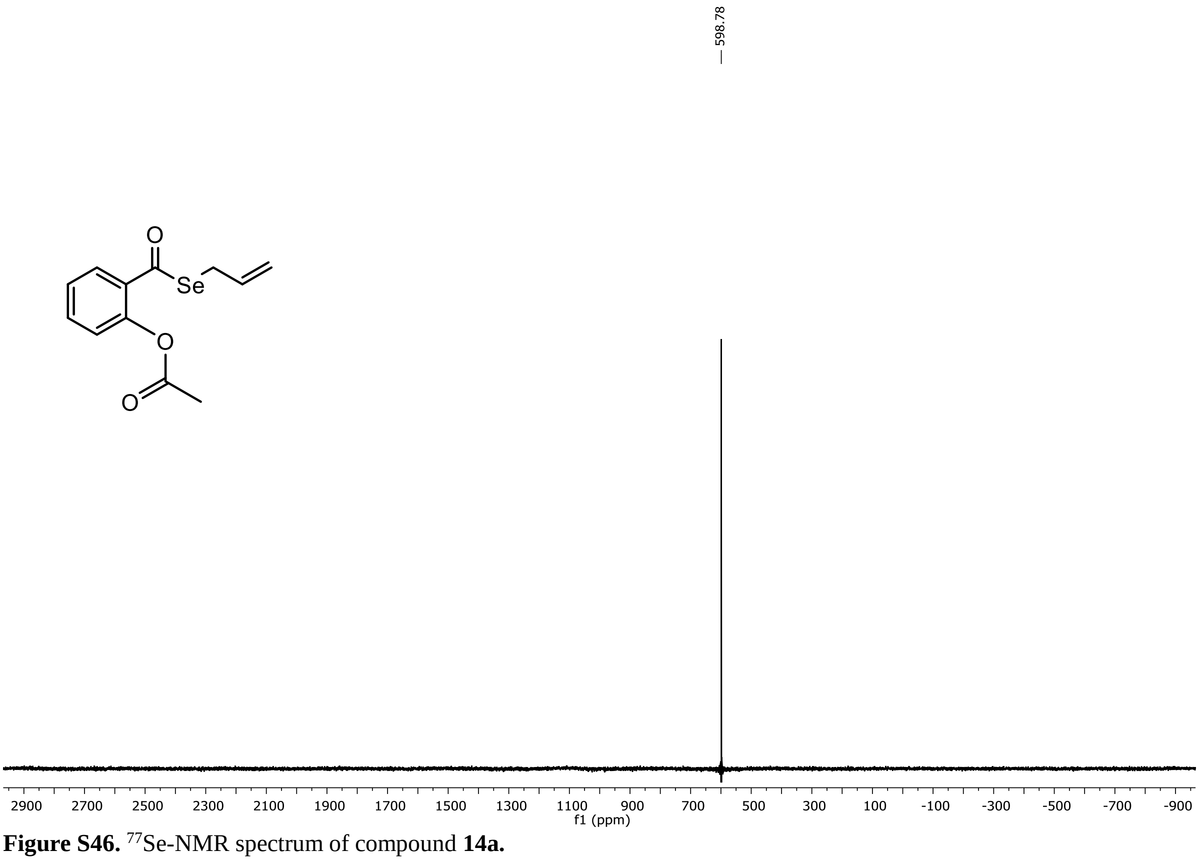

Figure S46. ^{77}Se-NMR spectrum of compound **14a.**

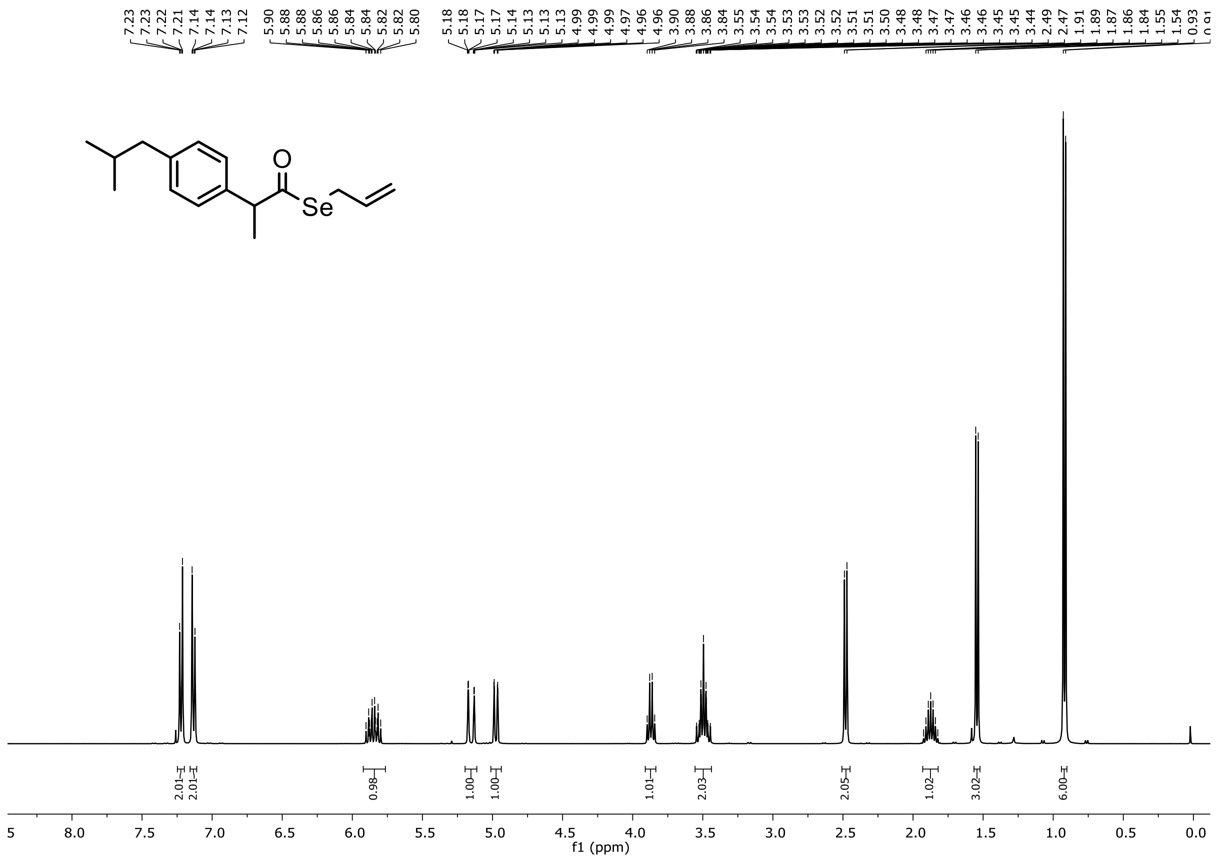

Figure S47. ^{1}H-NMR spectrum of compound **15a**.

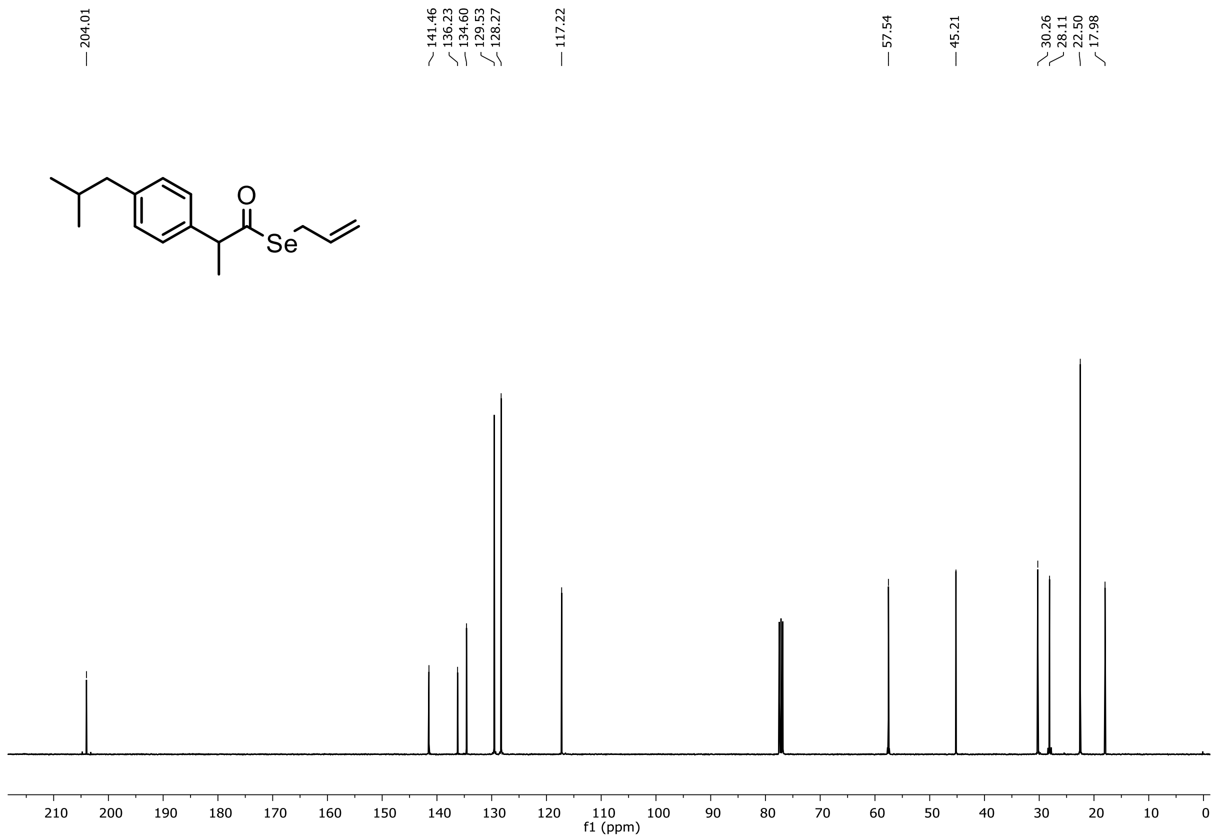

Figure S48. ^{13}C-NMR spectrum of compound **15a.**

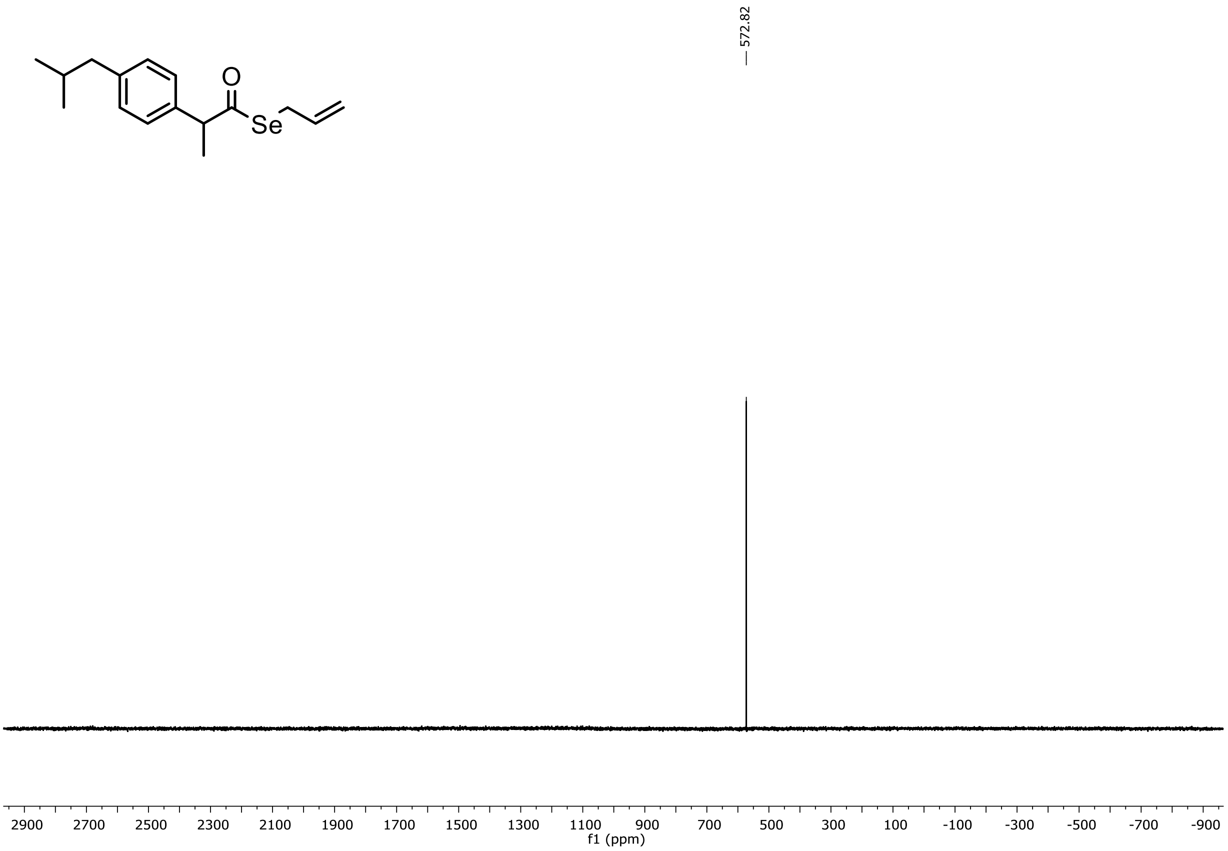

Figure S49.^{77}Se-NMR spectrum of compound **15a.**

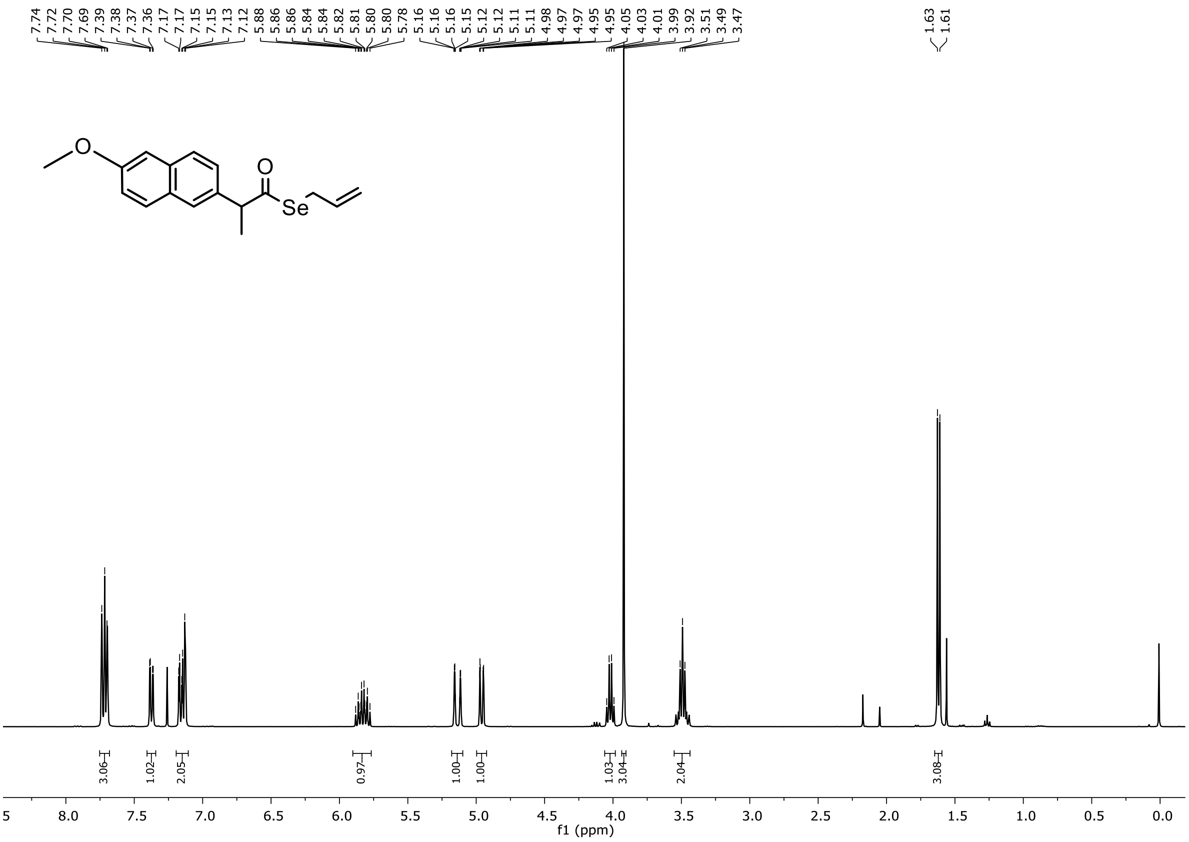

Figure S50. ^{1}H-NMR spectrum of compound **16a**.

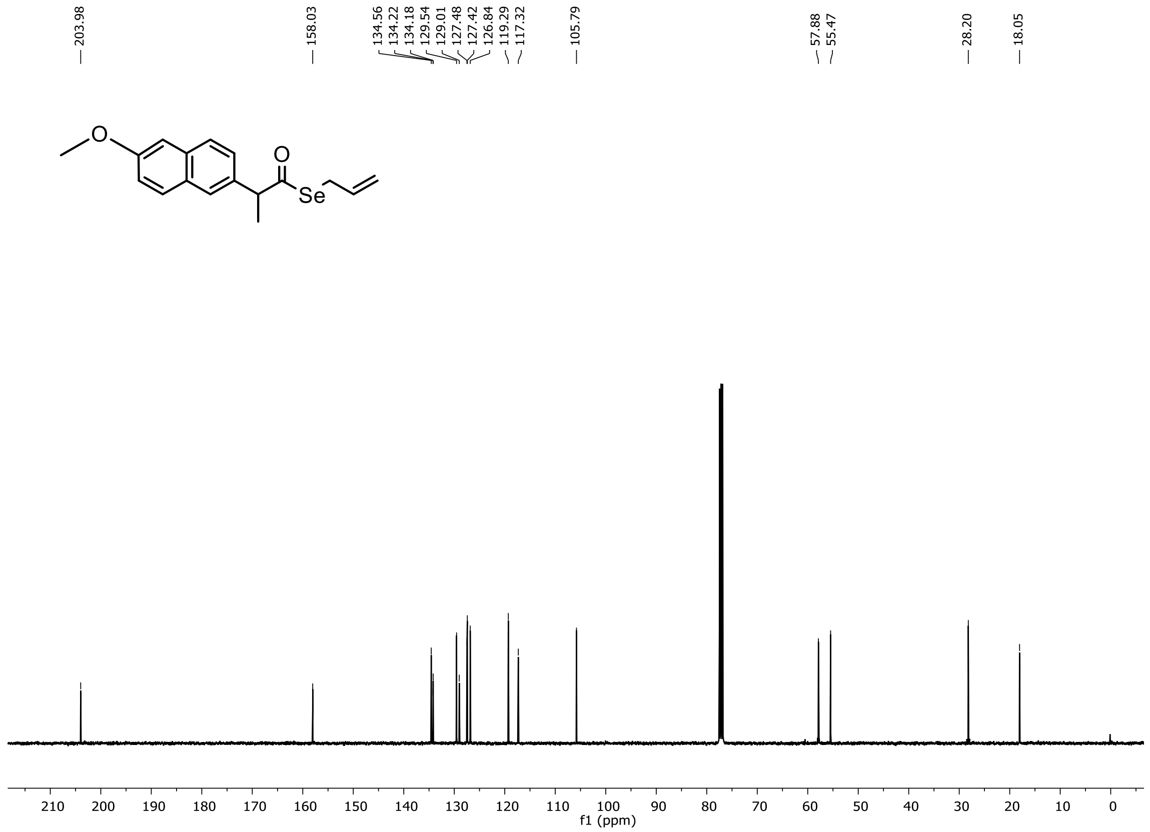

Figure S51. ^{13}C-NMR spectrum of compound **16a.**

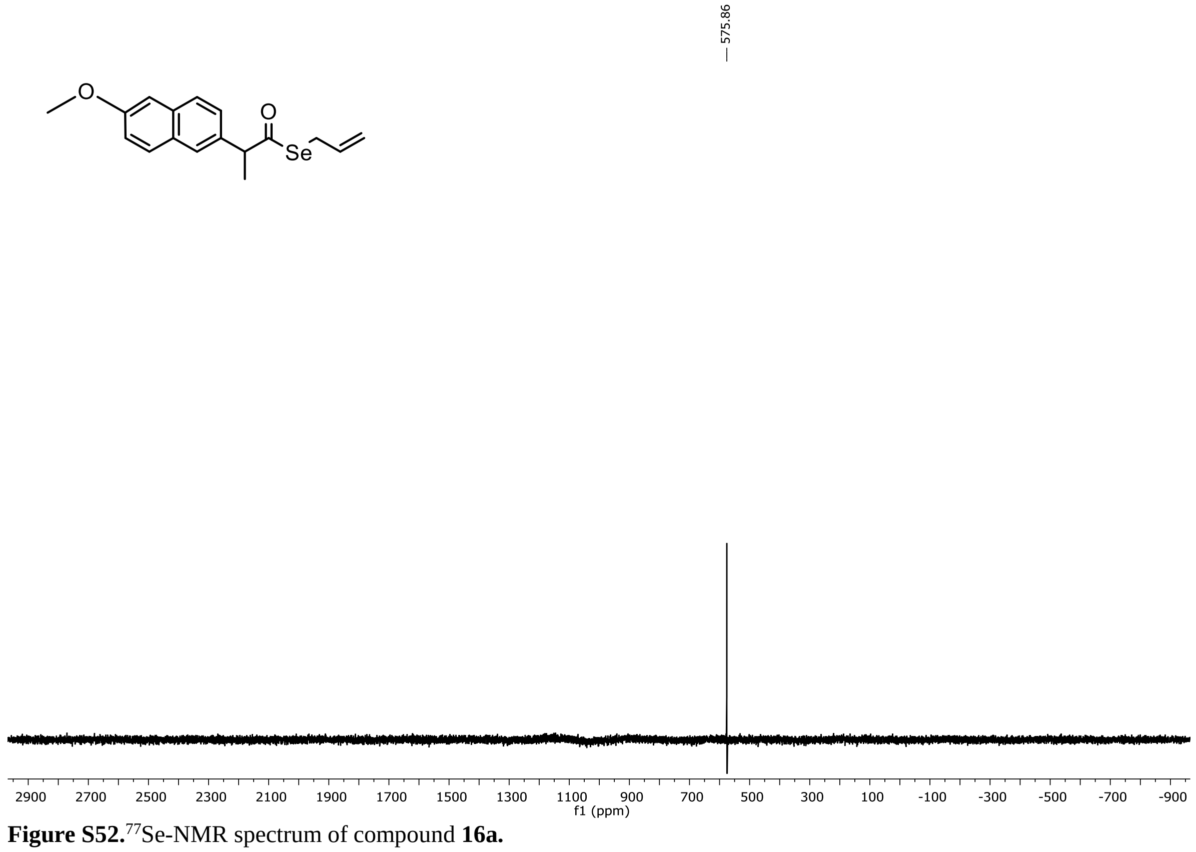

Figure S52.^{77}Se-NMR spectrum of compound **16a.**

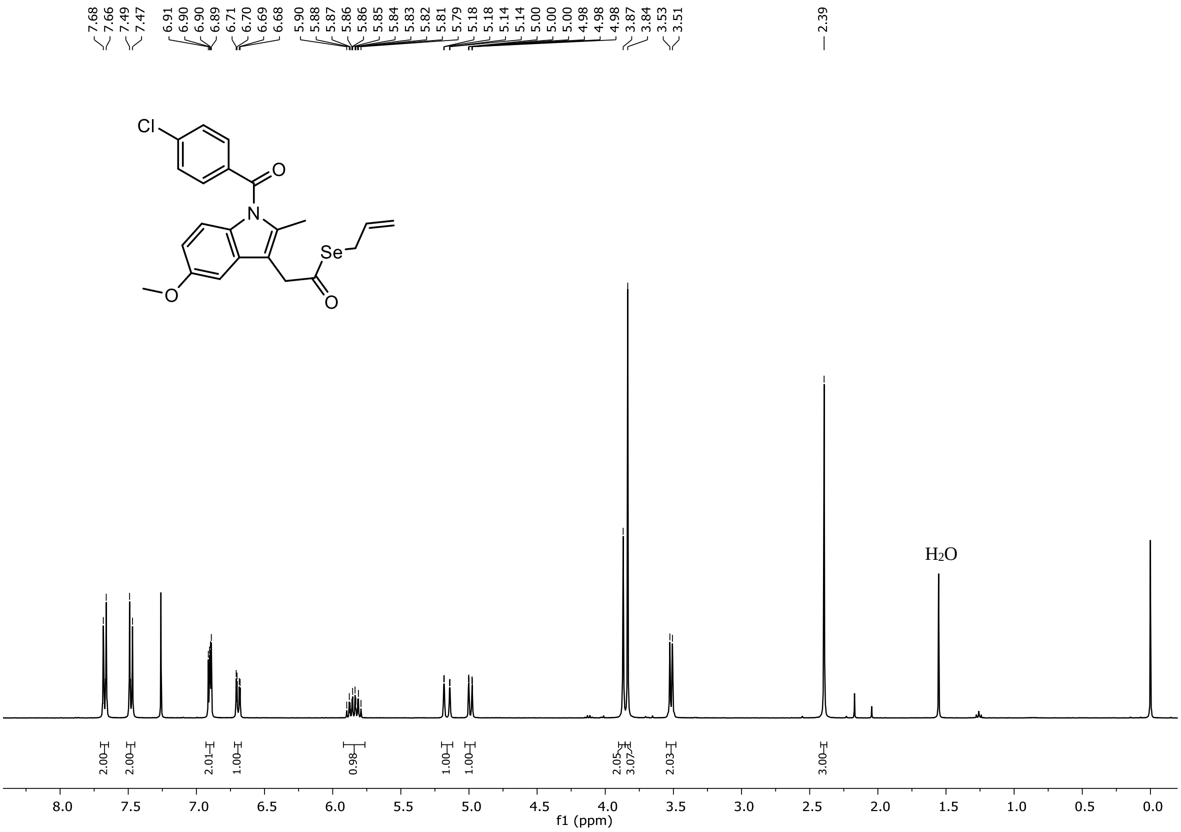

Figure S53. ^{1}H-NMR spectrum of compound **17a**.

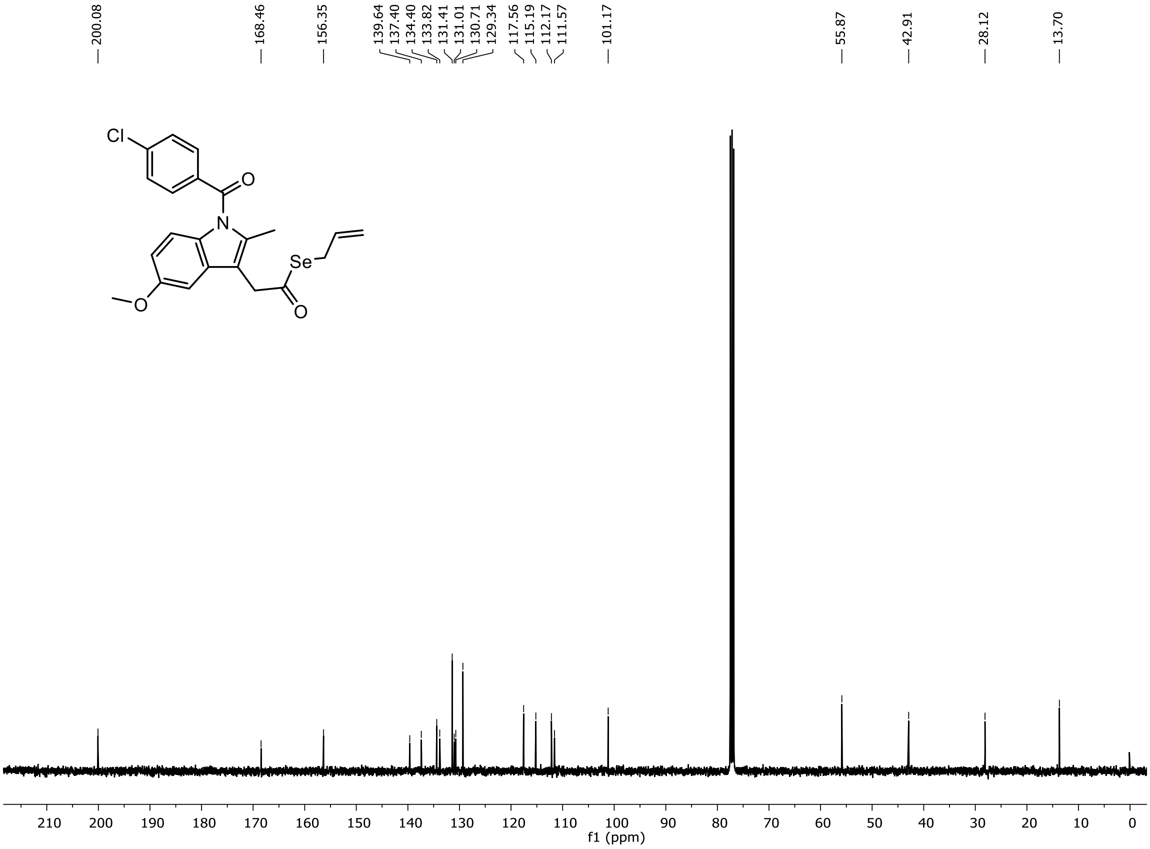

Figure S54. ^{13}C-NMR spectrum of compound **17a.**

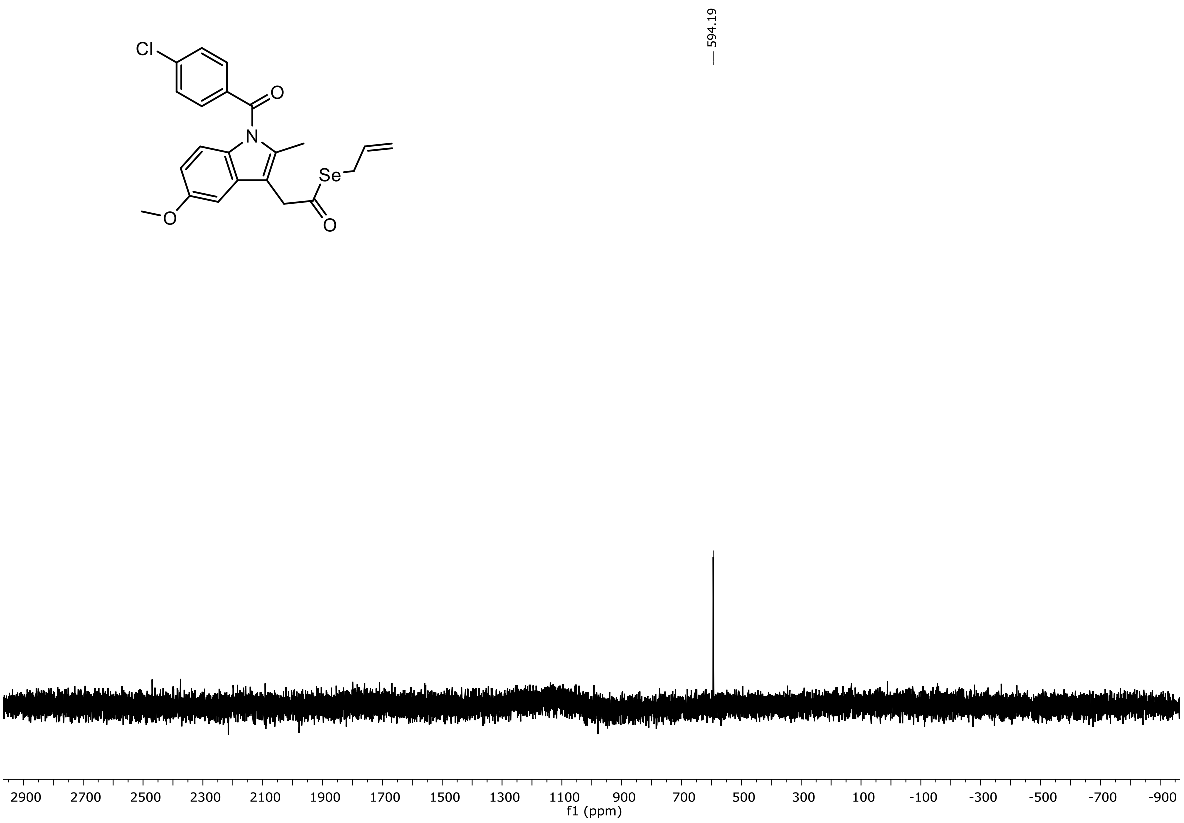

Figure S55.^{77}Se-NMR spectrum of compound **17a.**

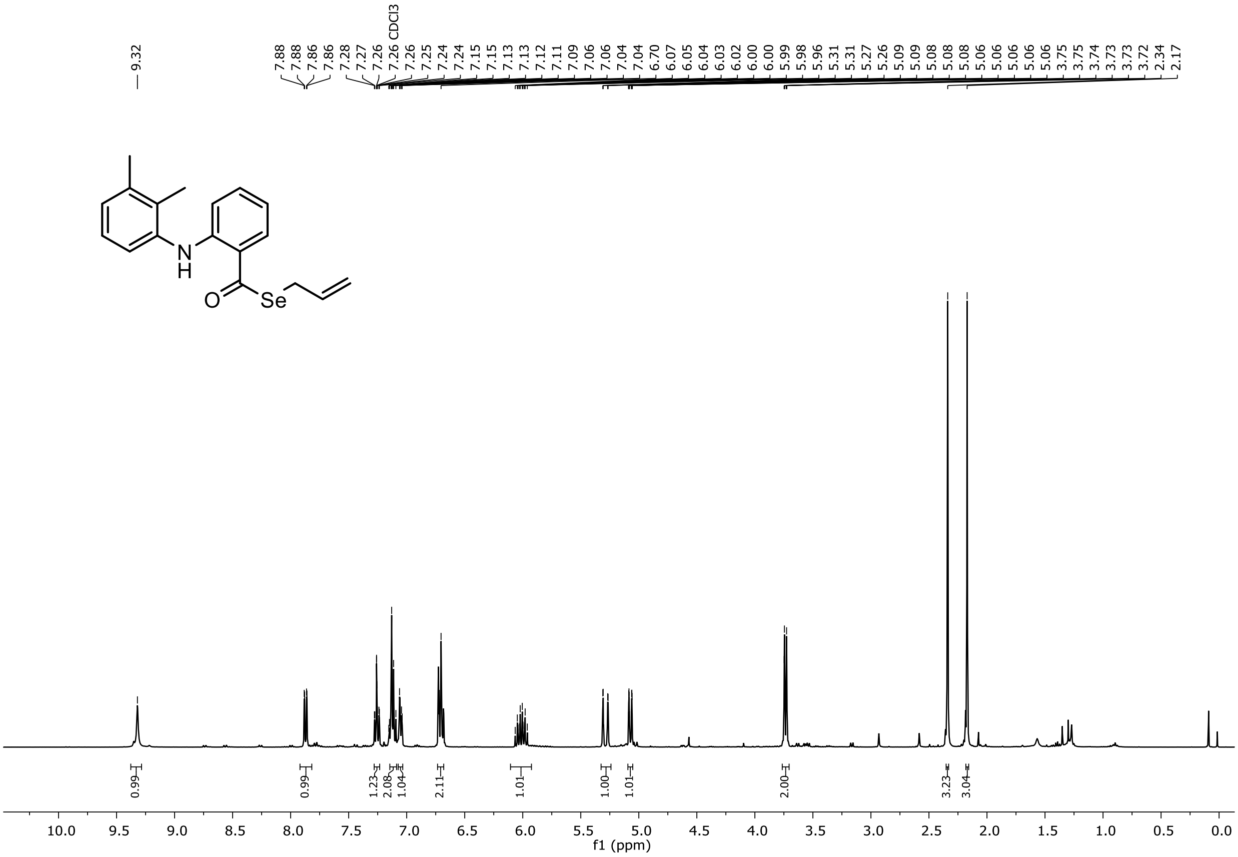

Figure S56. ^{1}H-NMR spectrum of compound **18a**.

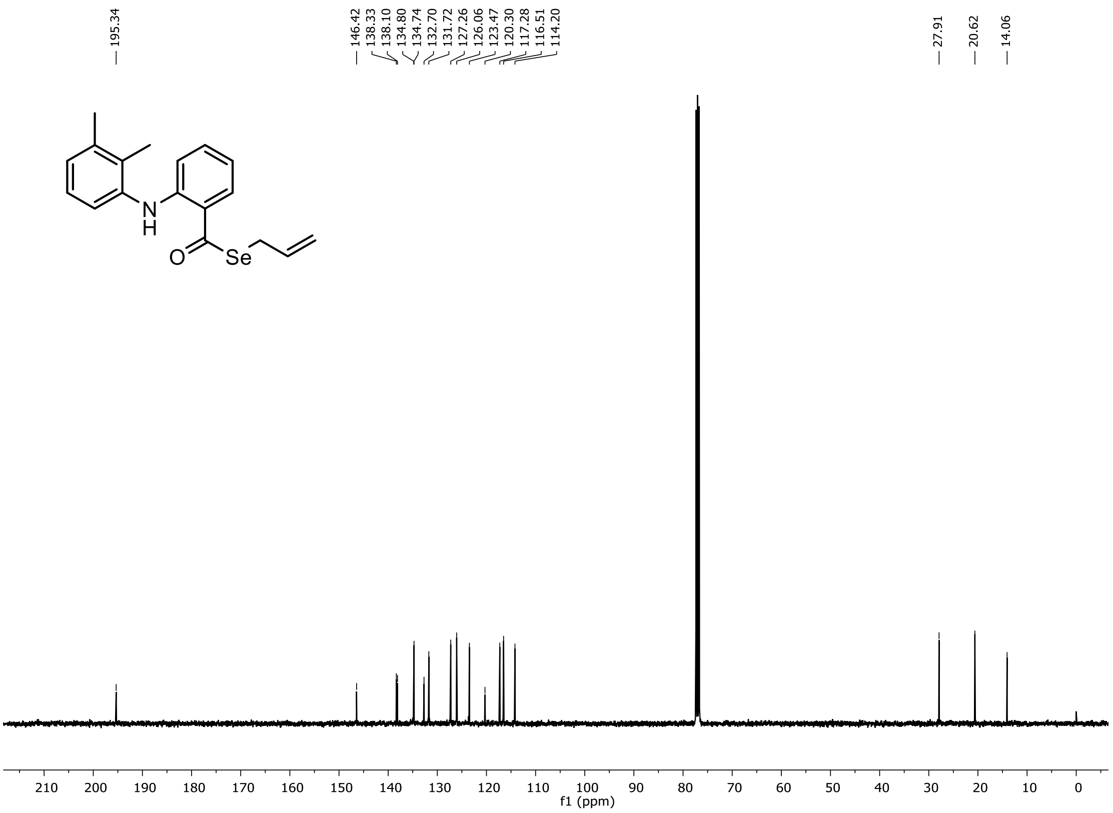

Figure S57. ^{13}C-NMR spectrum of compound **18a.**

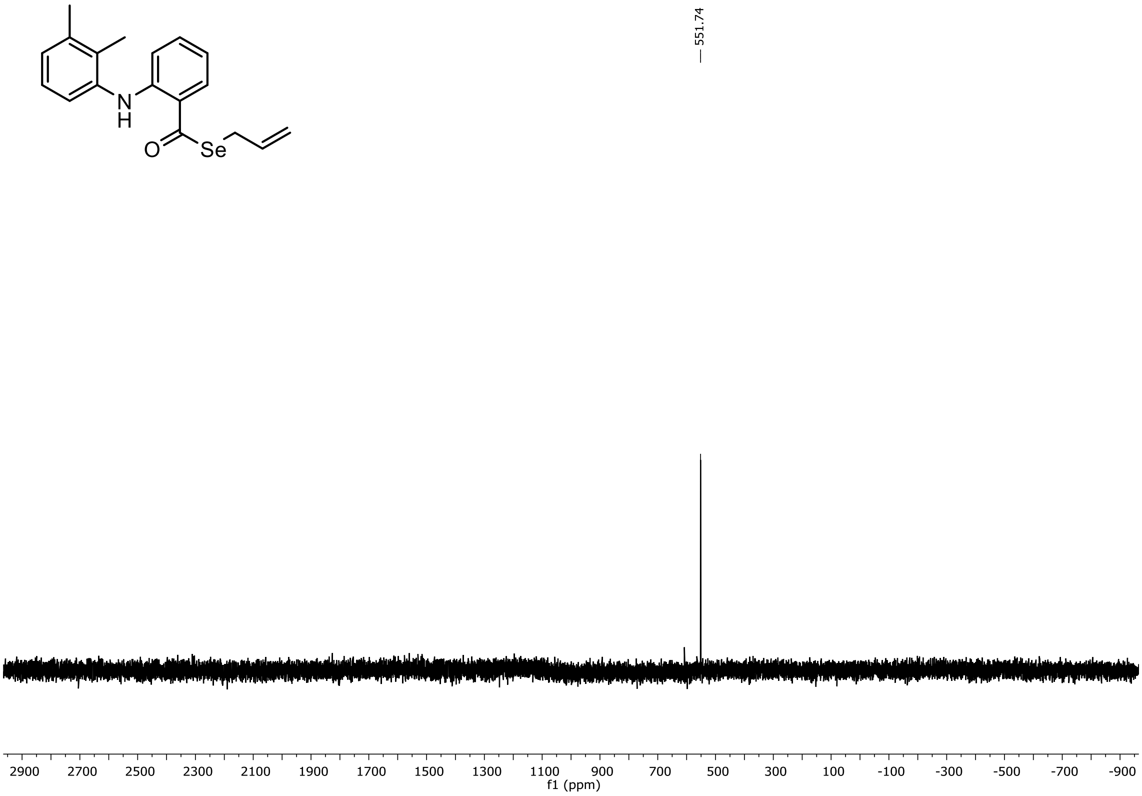

Figure S58.^{77}Se-NMR spectrum of compound **18a.**

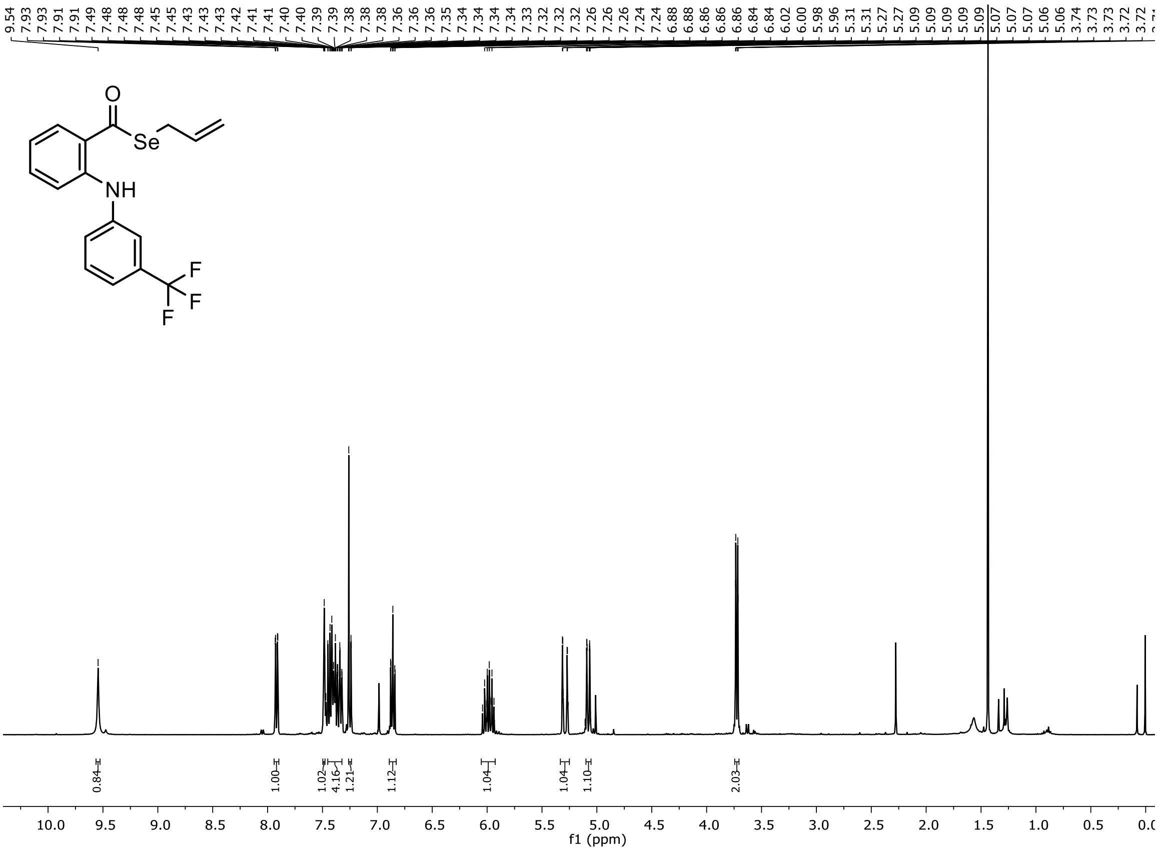

Figure S59. ^{1}H-NMR spectrum of compound **19a**.

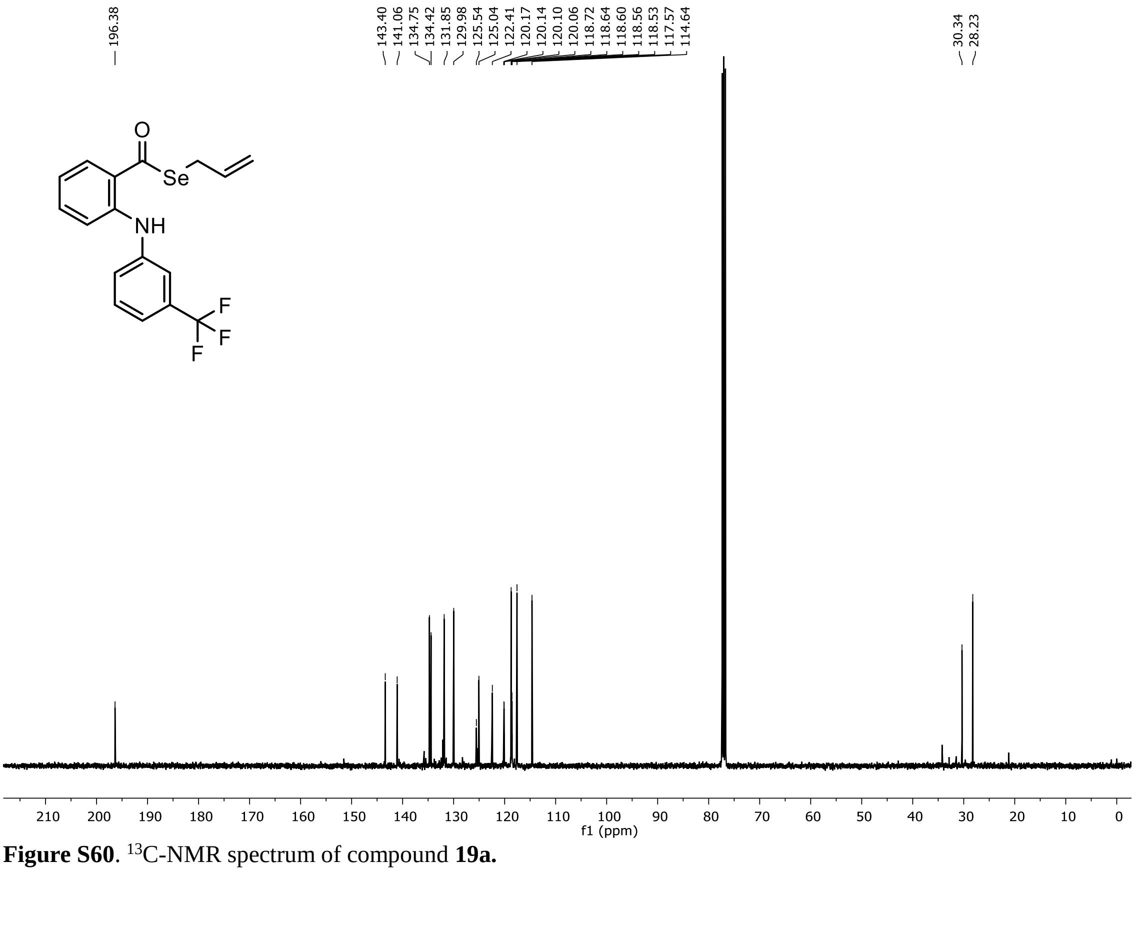

Figure S60. ^{13}C-NMR spectrum of compound **19a.**

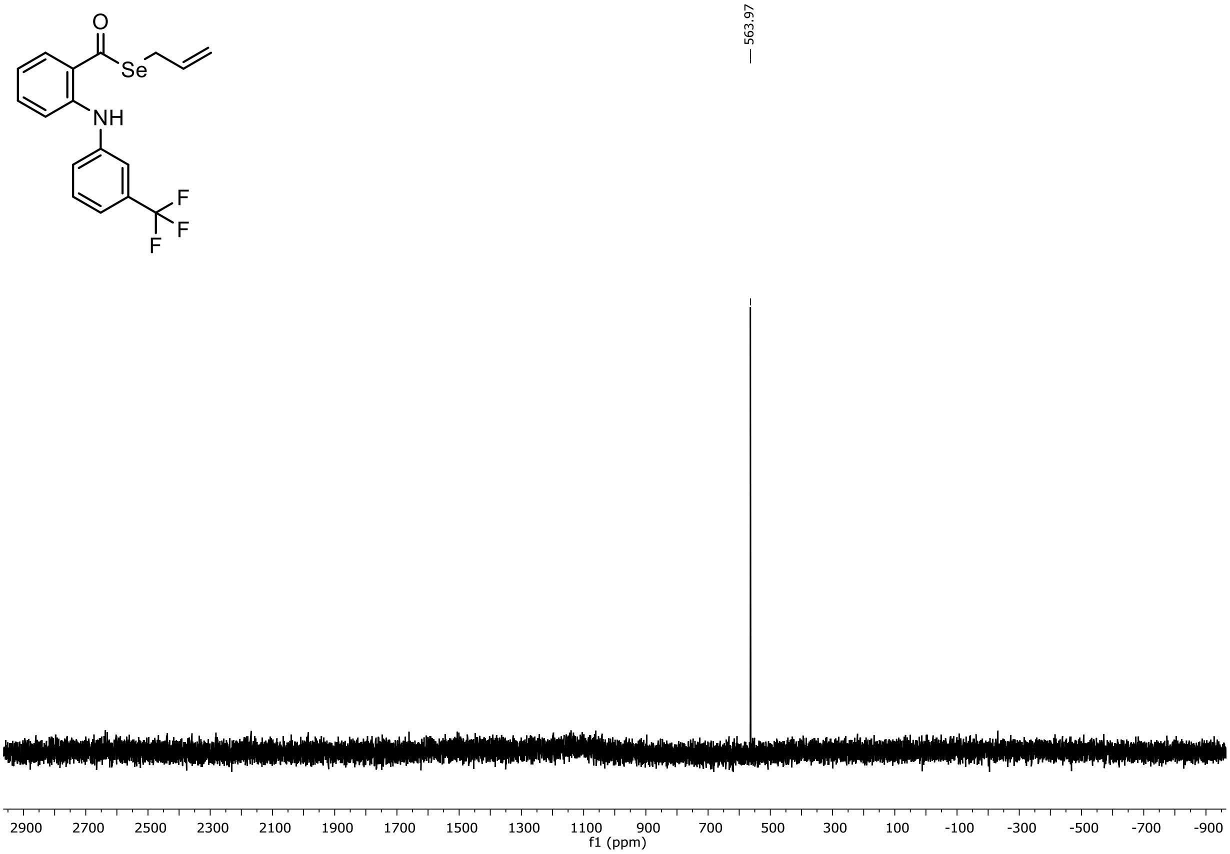

Figure S61. ^{77}Se-NMR spectrum of compound **19a.**

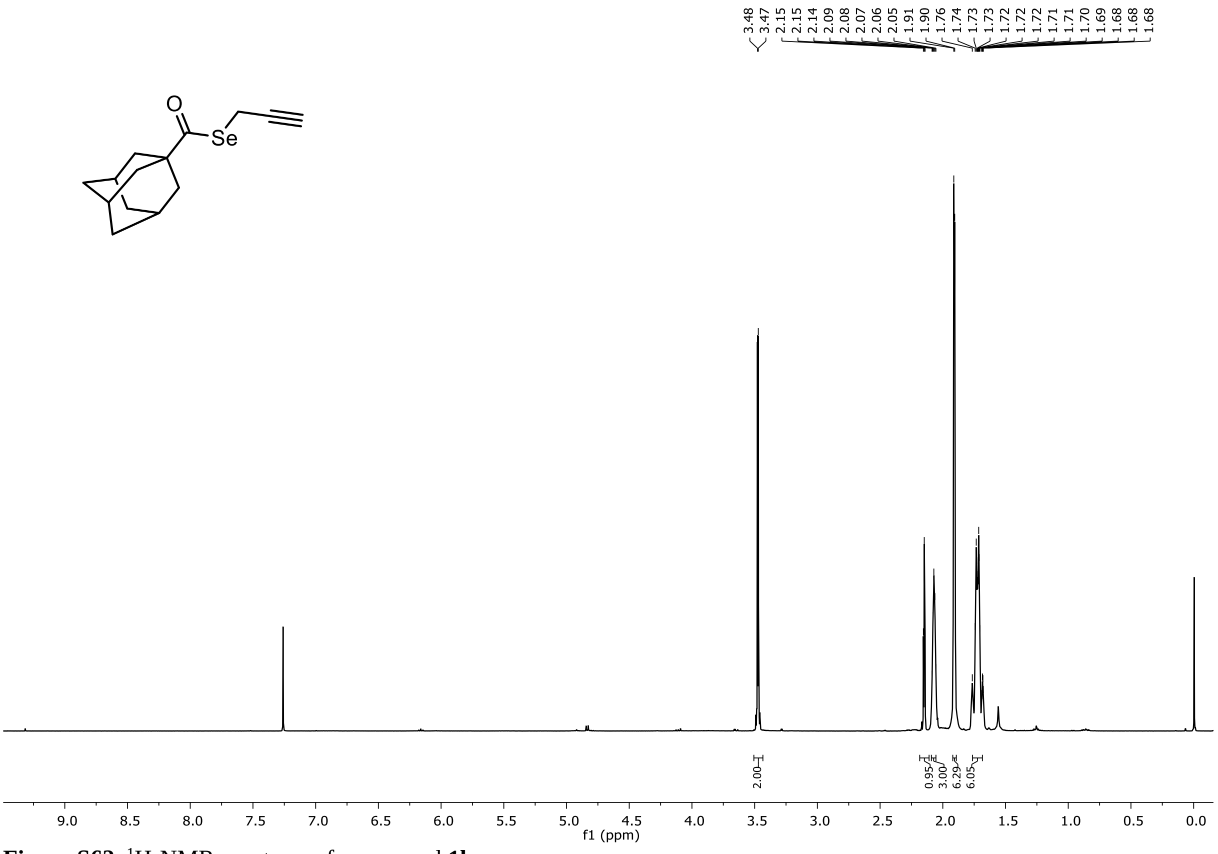

Figure S62. ^{1}H-NMR spectrum of compound **1b**.

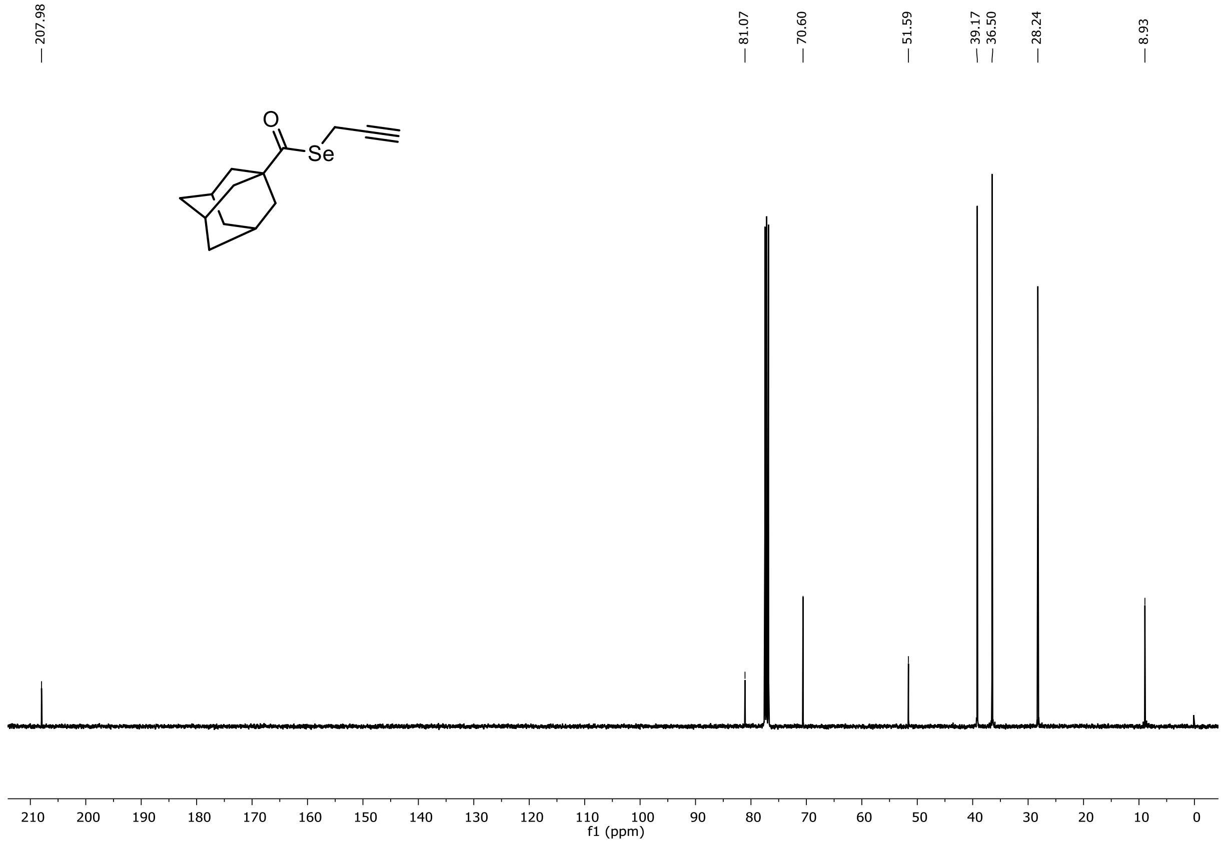

Figure S63. ^{13}C-NMR spectrum of compound **1b.**

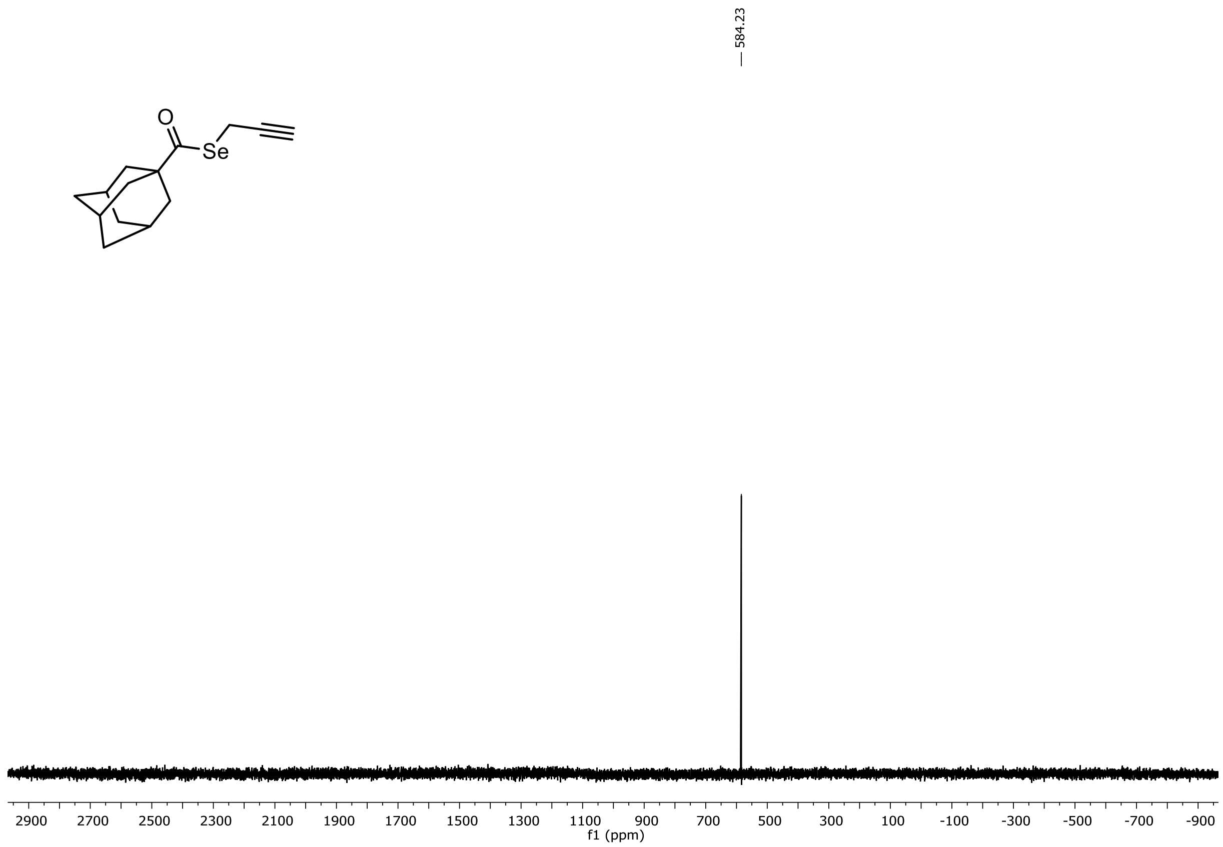

Figure S64.^{77}Se-NMR spectrum of compound **1b.**

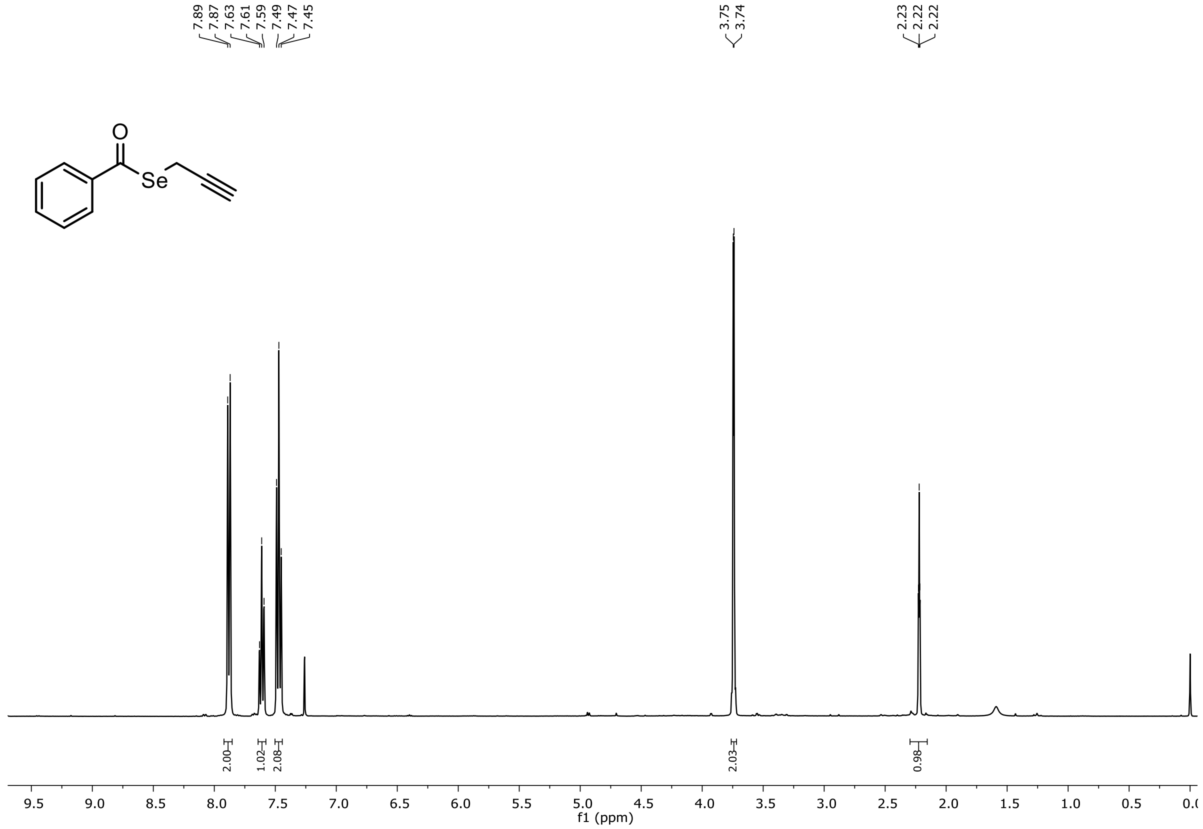

Figure S65. ^{1}H-NMR spectrum of compound **2b**.

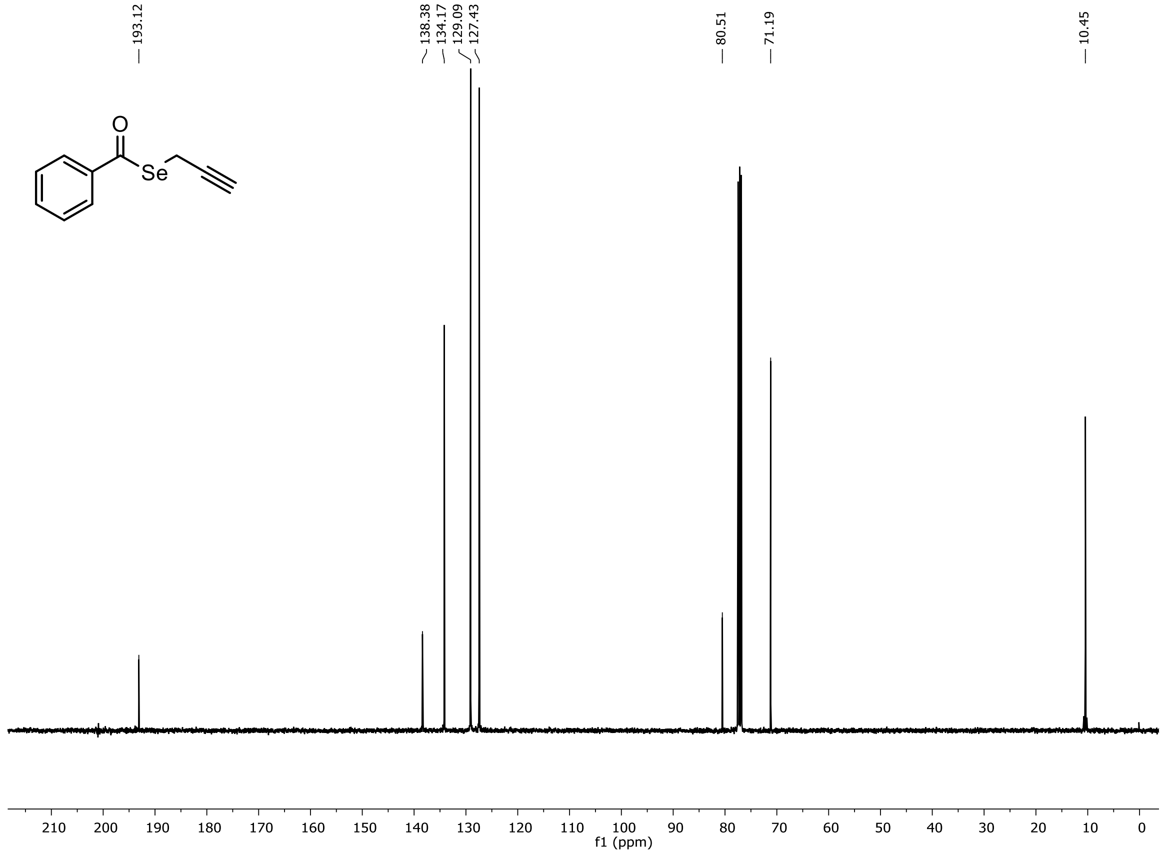

Figure S66. ^{13}C-NMR spectrum of compound **2b.**

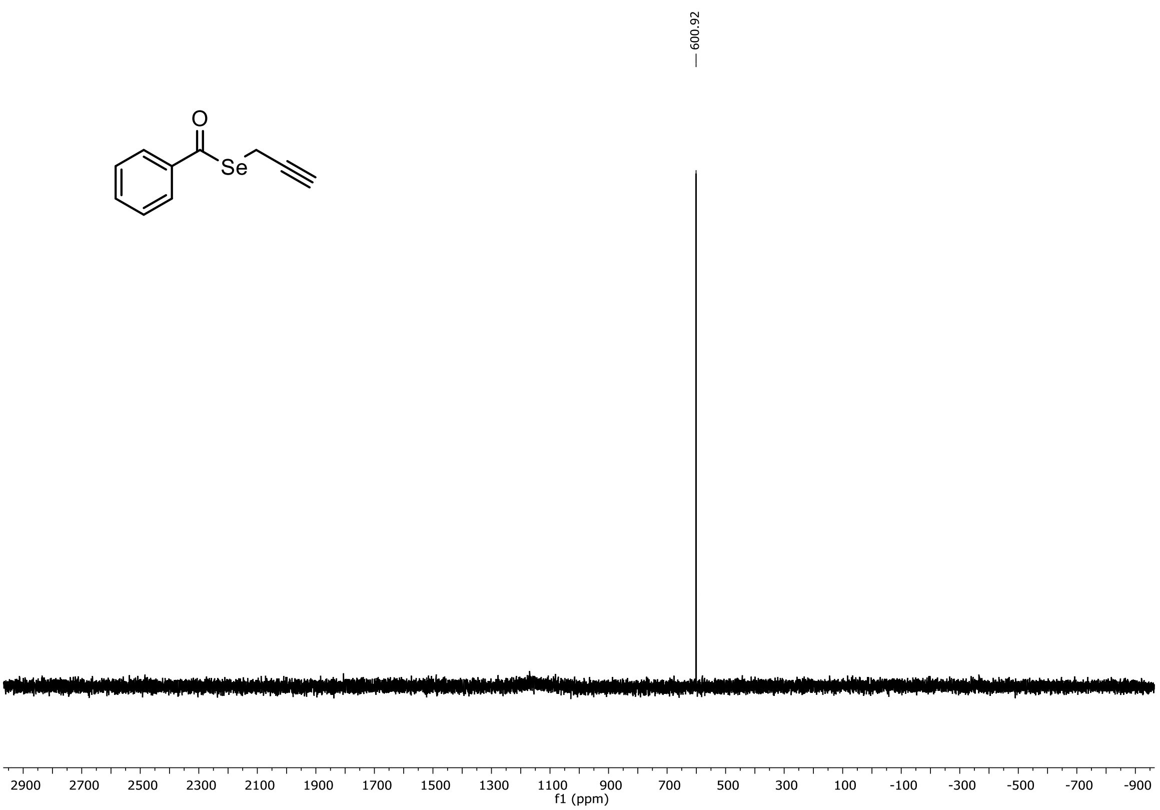

Figure S67.^{77}Se-NMR spectrum of compound **2b.**

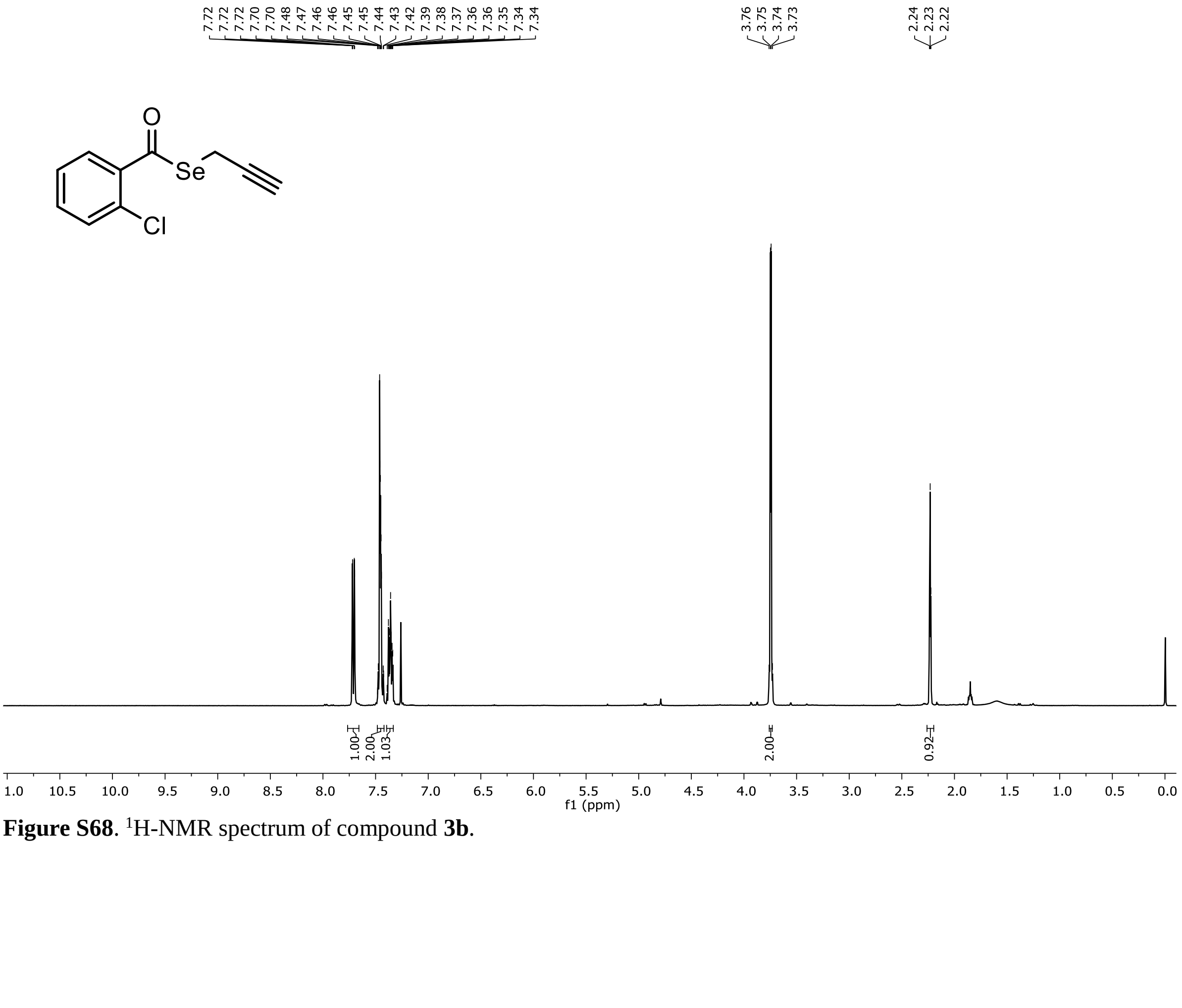

Figure S68. ^{1}H-NMR spectrum of compound **3b**.

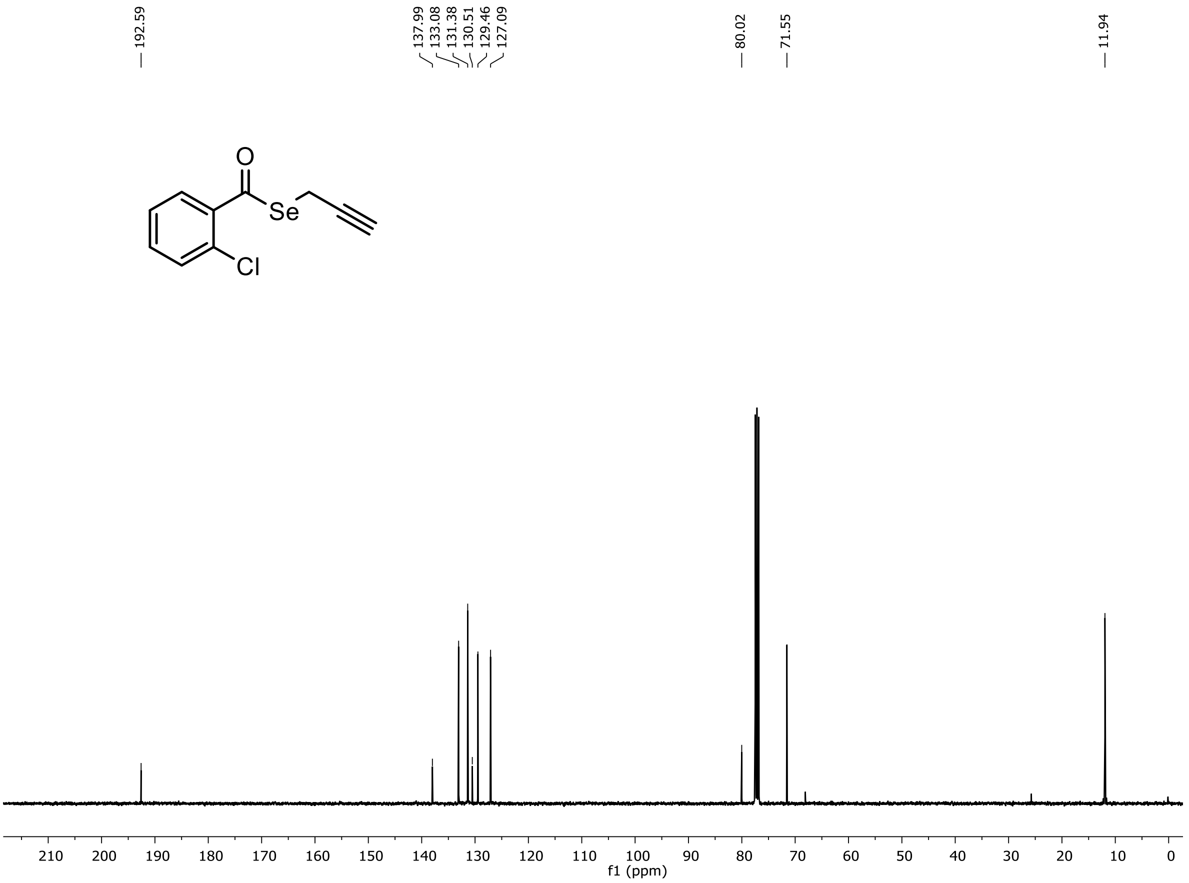

Figure S69. ^{13}C-NMR spectrum of compound **3b.**

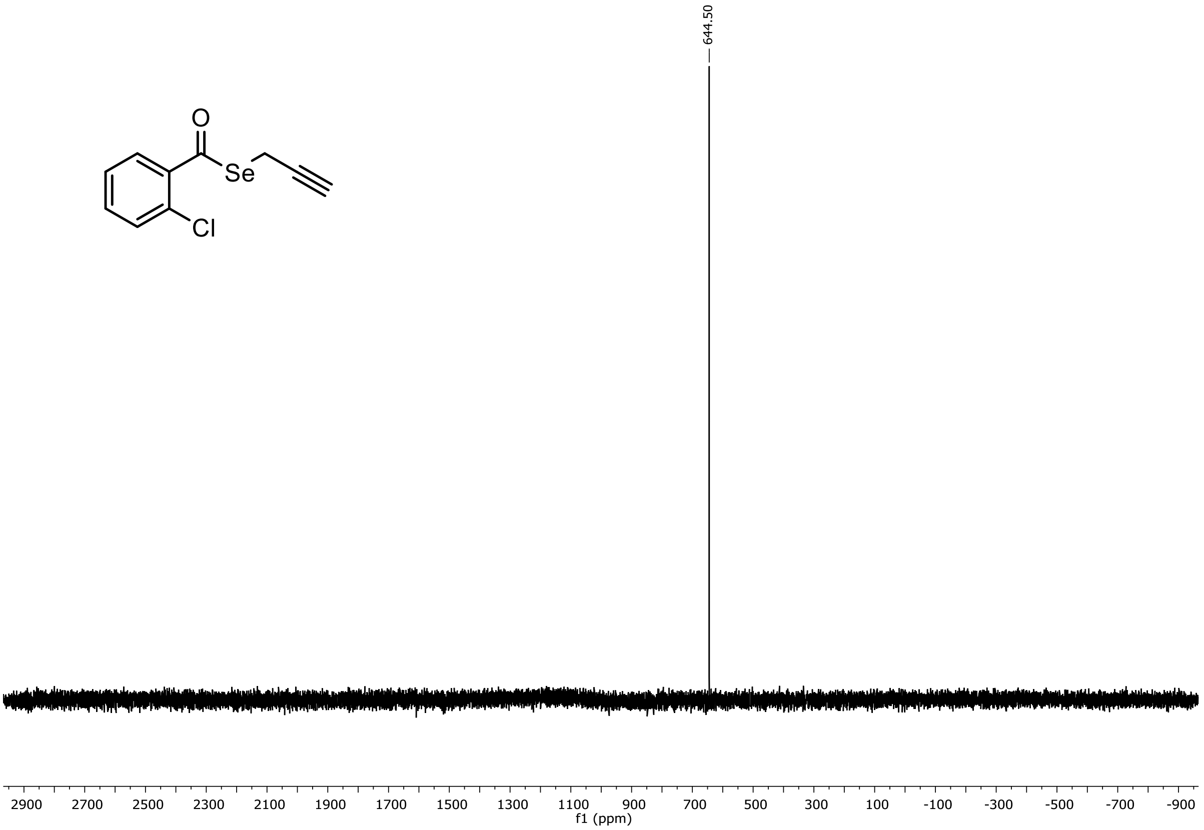

Figure S70.^{77}Se-NMR spectrum of compound **3b.**

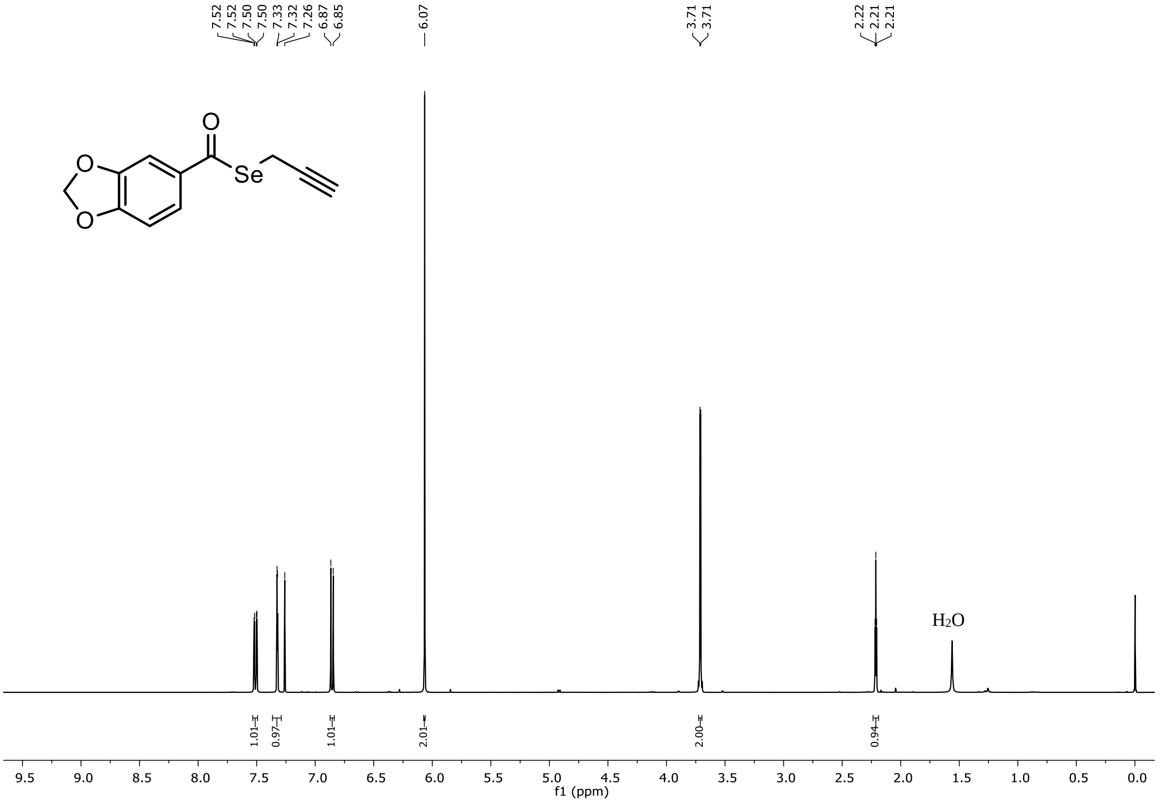

Figure S71. ^{1}H-NMR spectrum of compound **4b.**

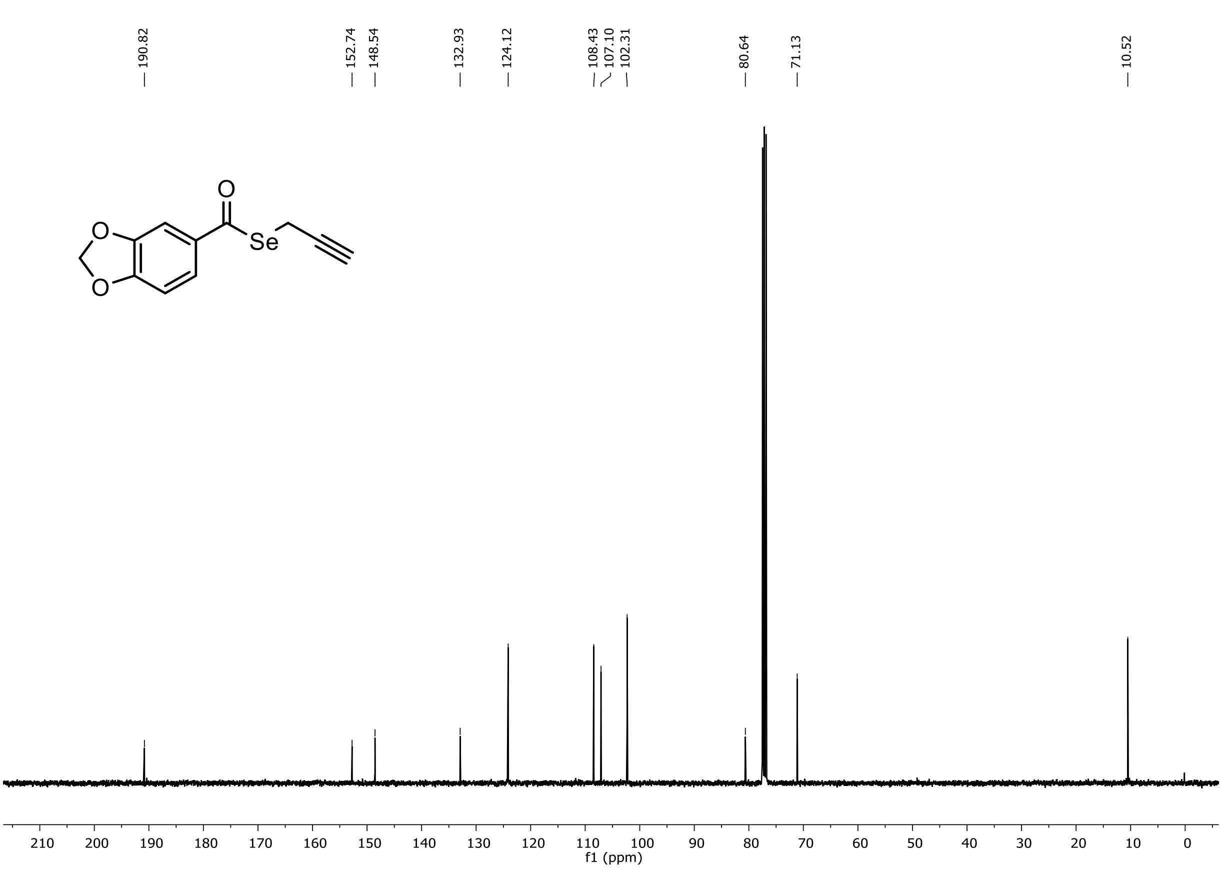

Figure S72. ^{13}C-NMR spectrum of compound **4b.**

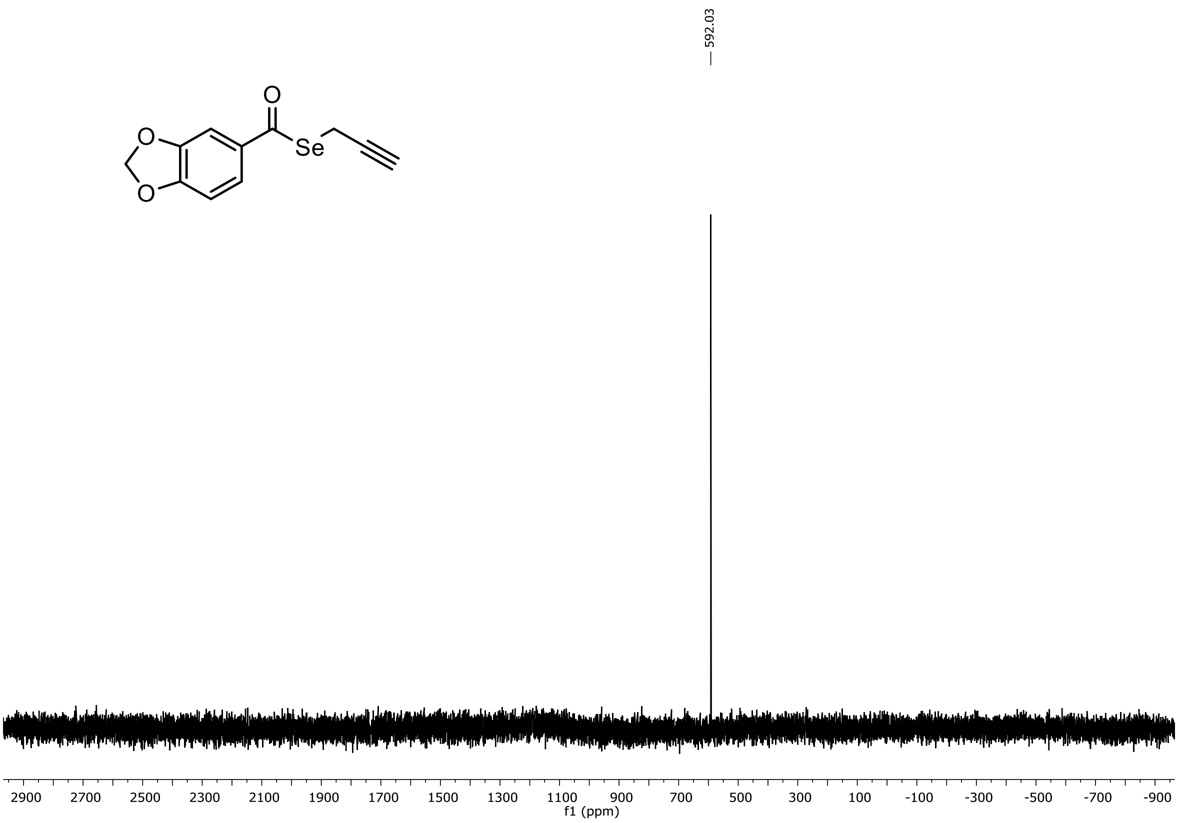

Figure S73.^{77}Se-NMR spectrum of compound **4b.**

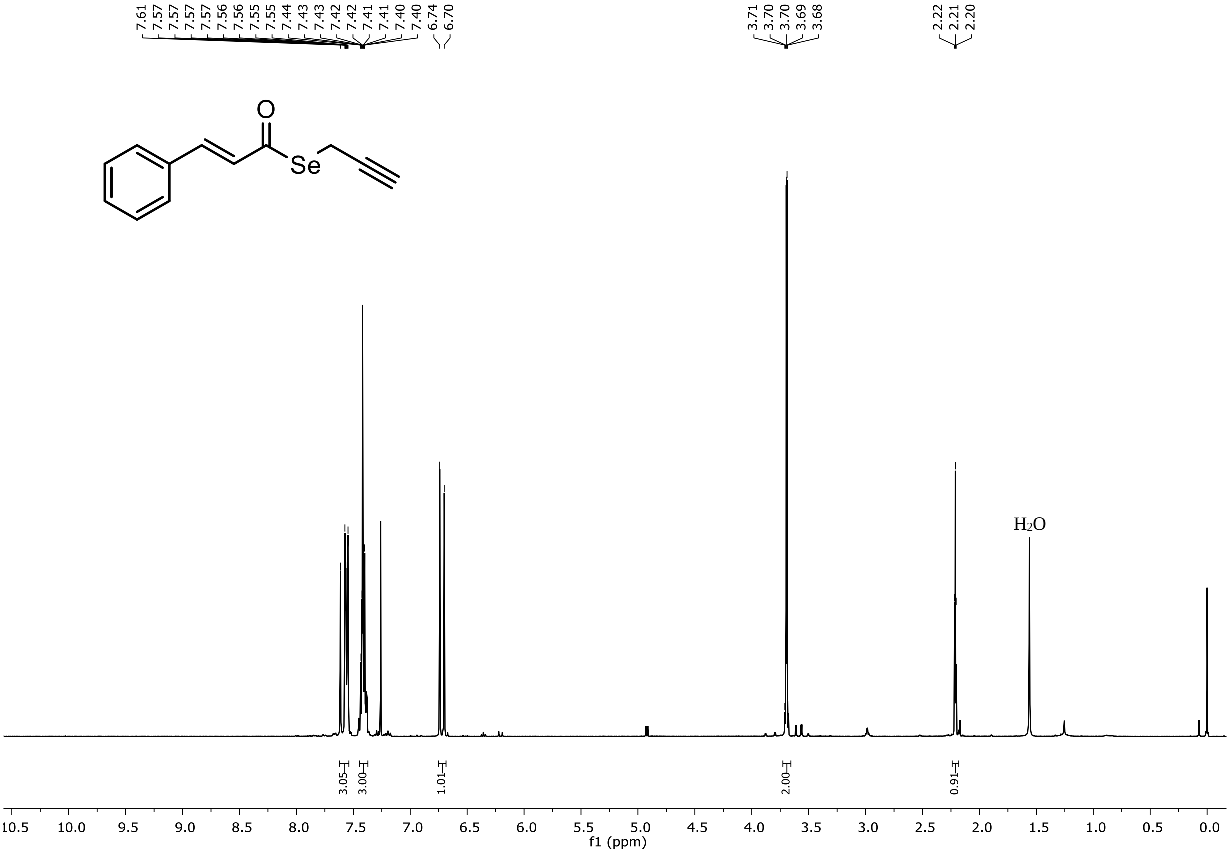

Figure S74. ^{1}H-NMR spectrum of compound **5b.**

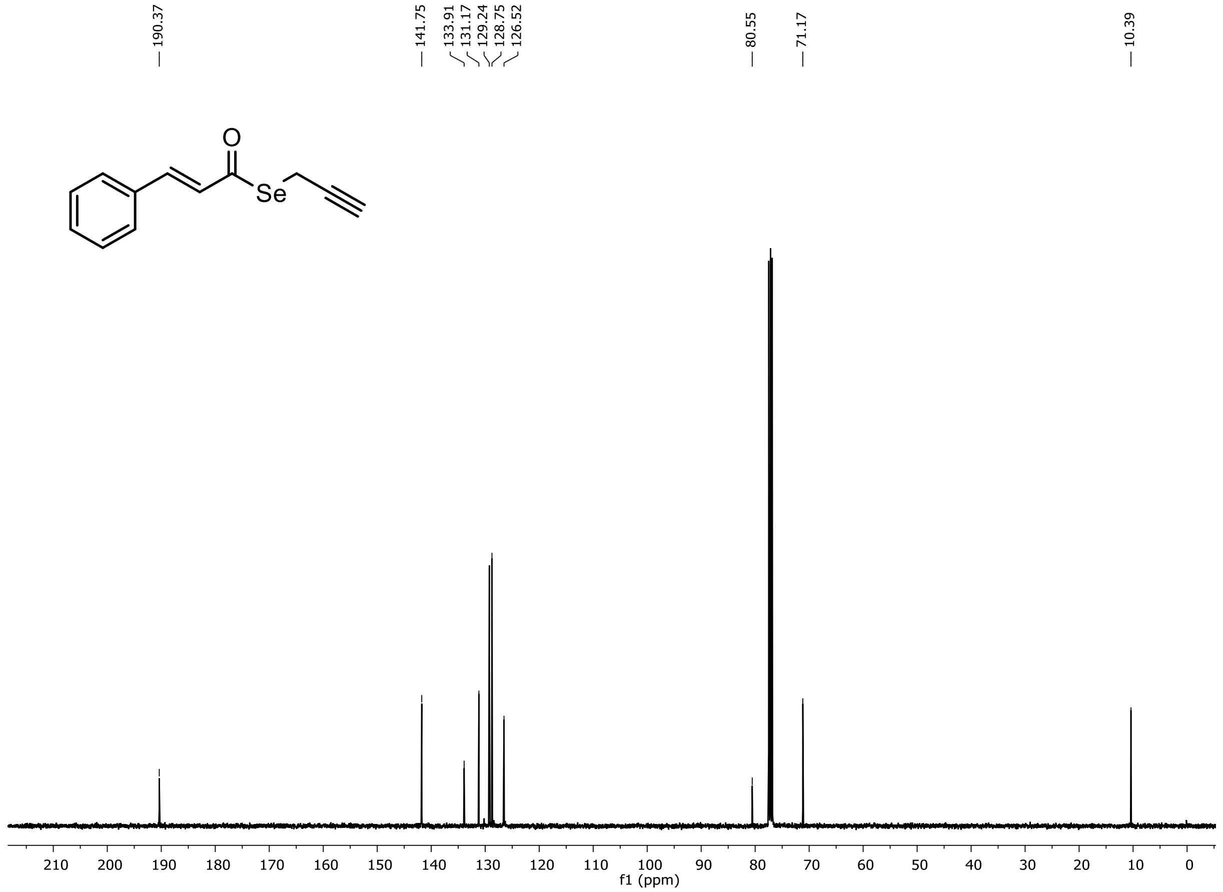

Figure S75. ^{13}C-NMR spectrum of compound **5b.**

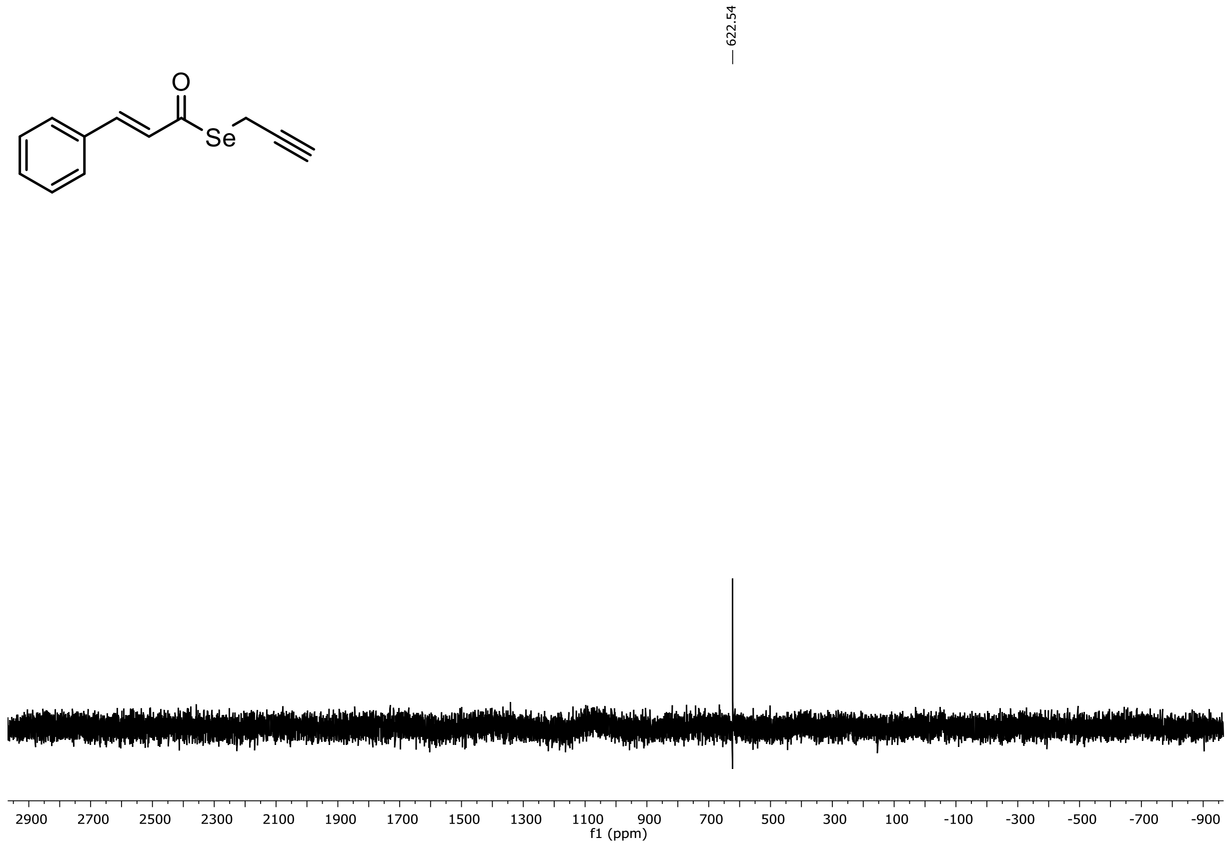

Figure S76.^{77}Se-NMR spectrum of compound **5b.**

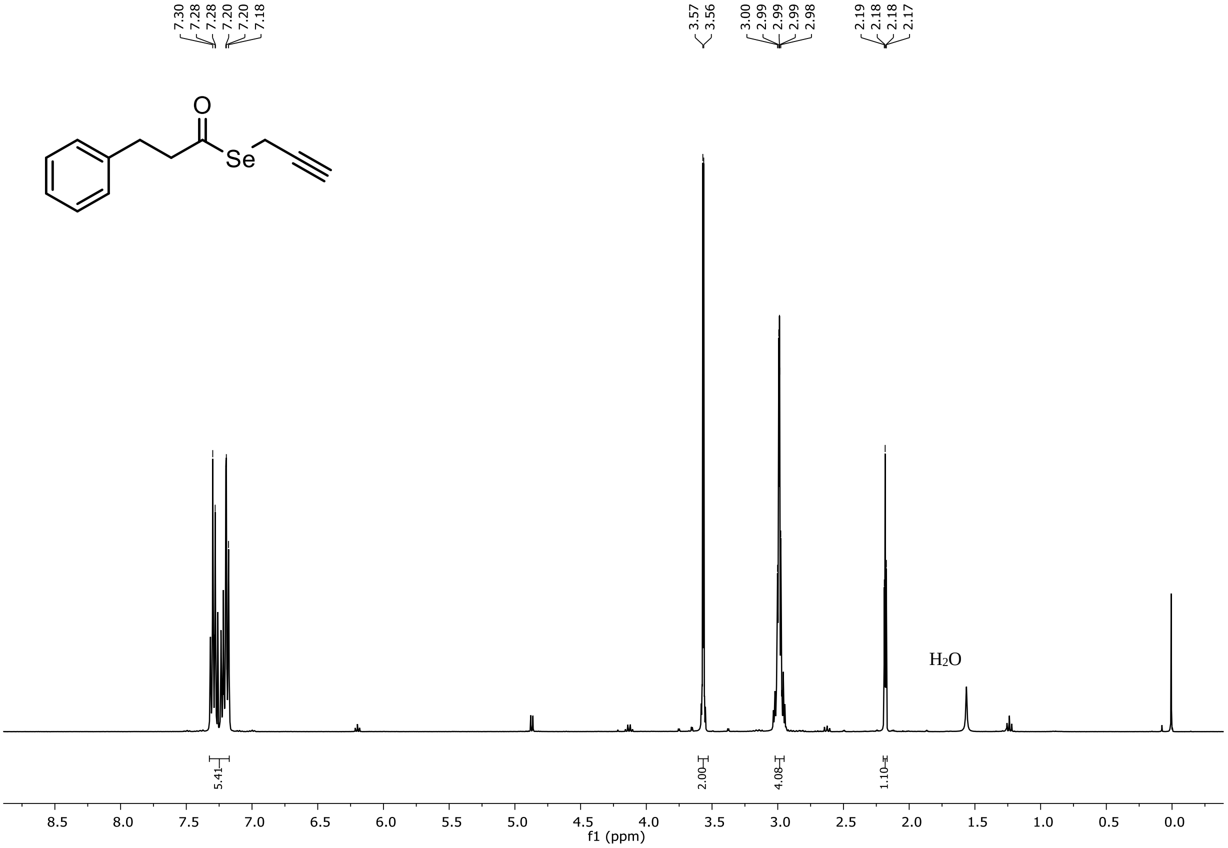

Figure S77. ^{1}H-NMR spectrum of compound **6b.**

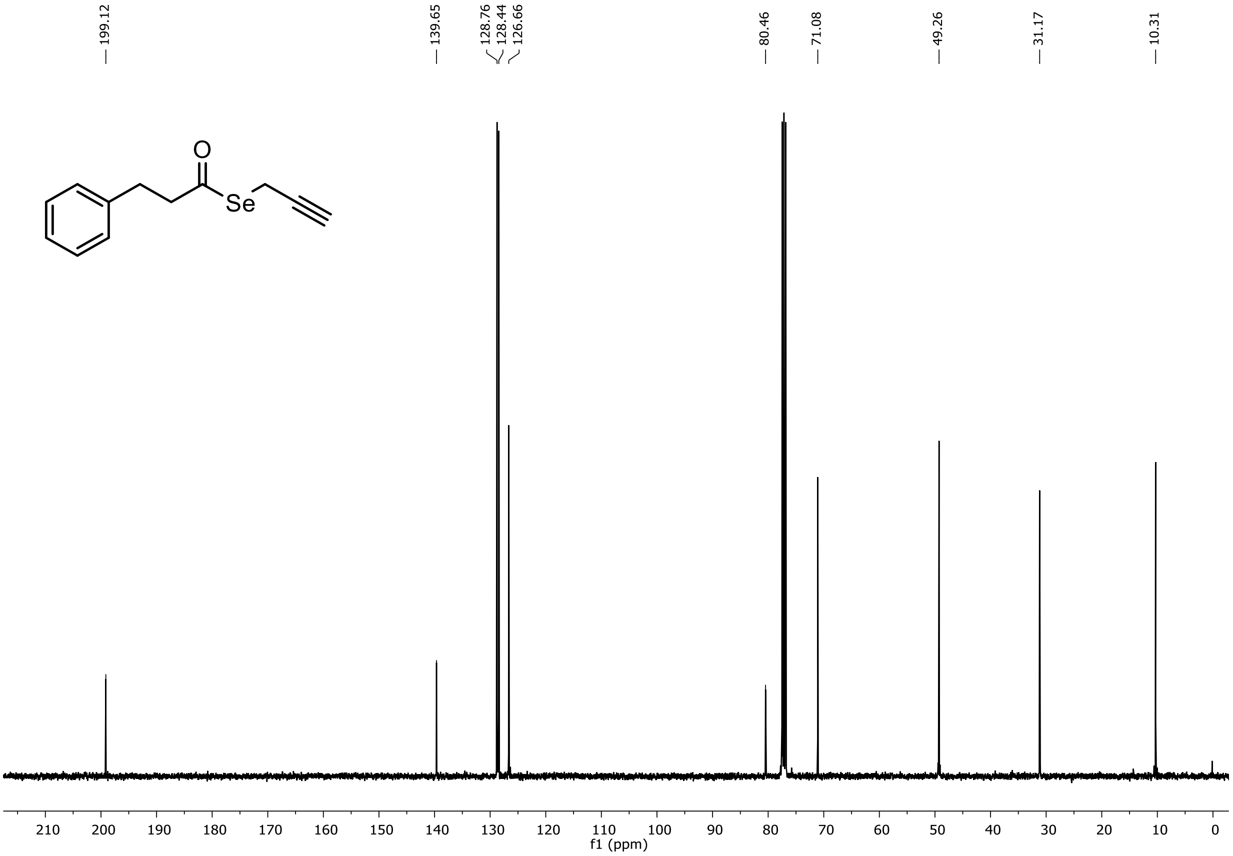

Figure S78. ^{13}C-NMR spectrum of compound **6b.**

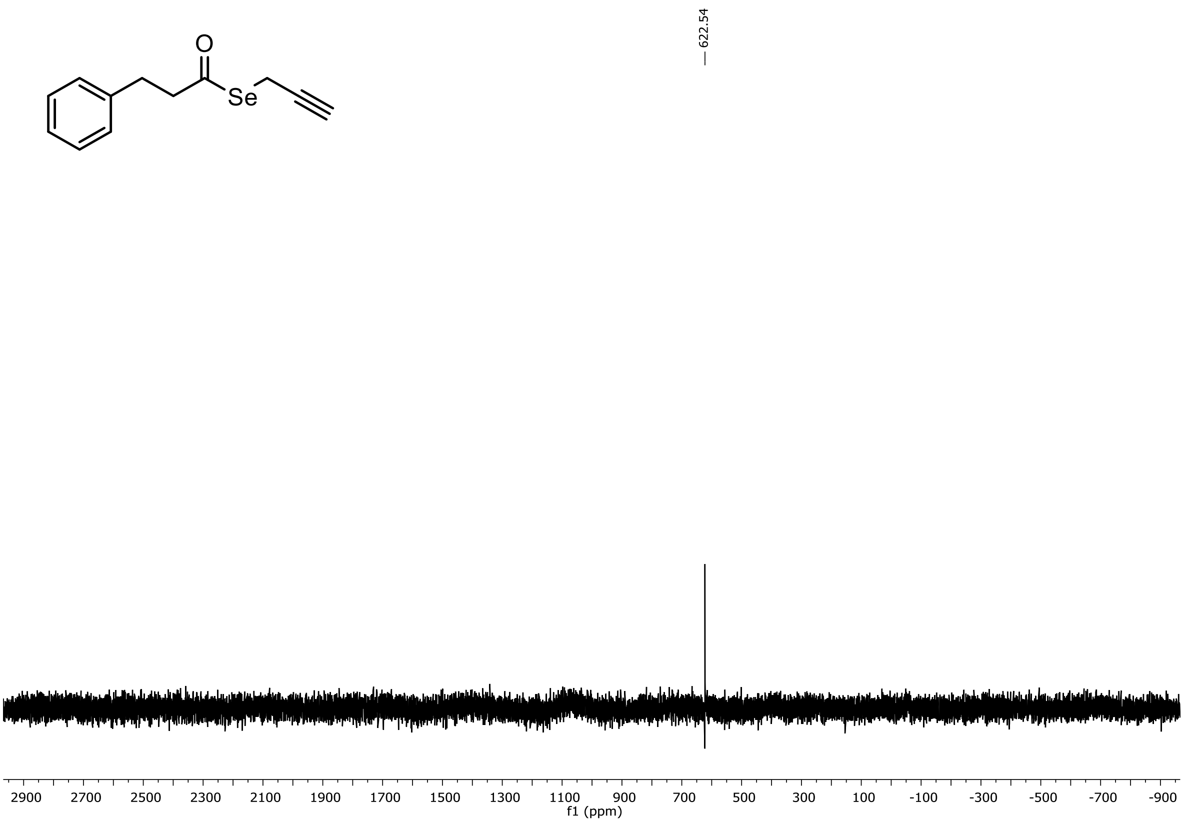

Figure S79. ^{77}Se-NMR spectrum of compound **6b.**

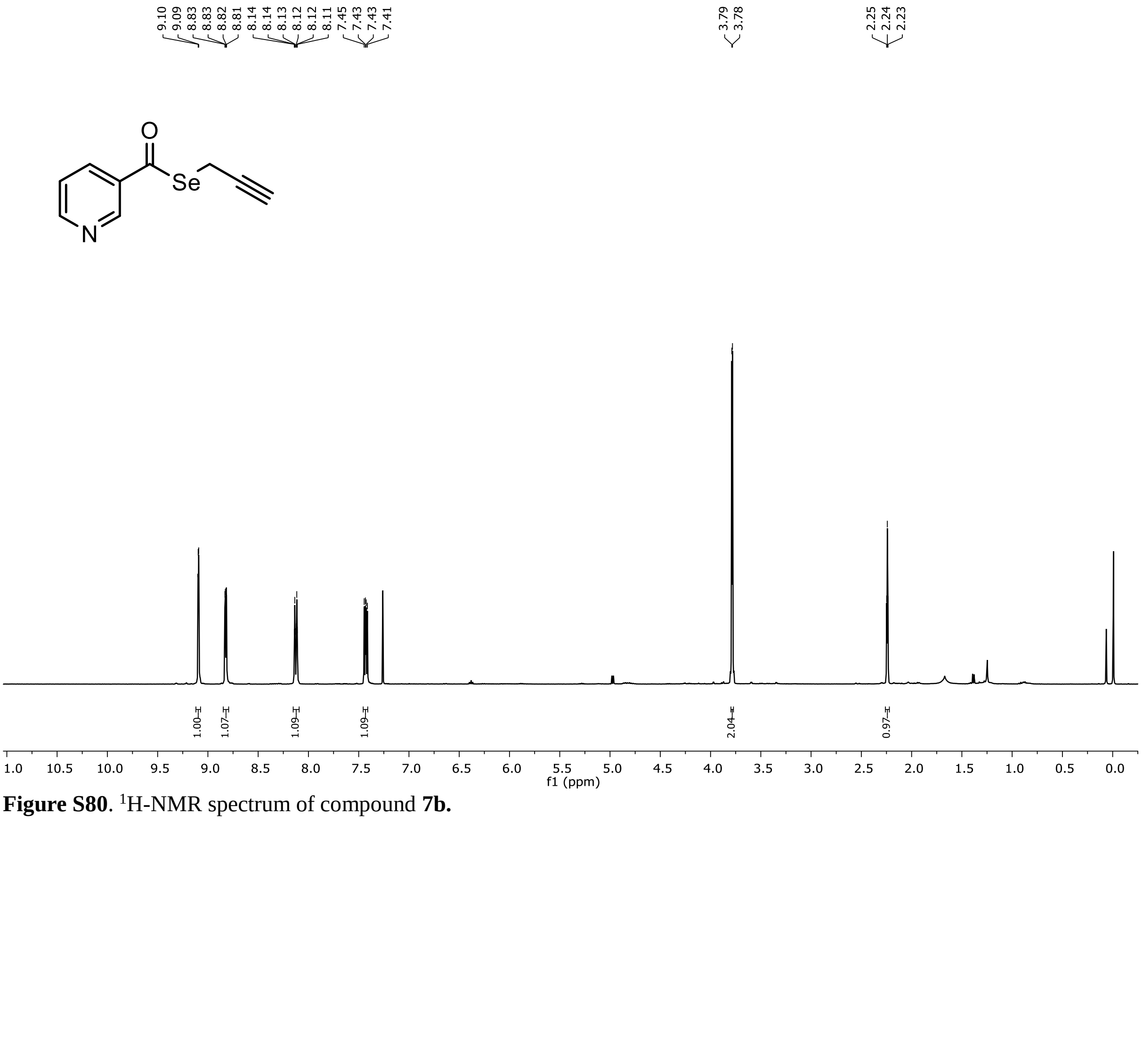

Figure S80. ^{1}H-NMR spectrum of compound **7b.**

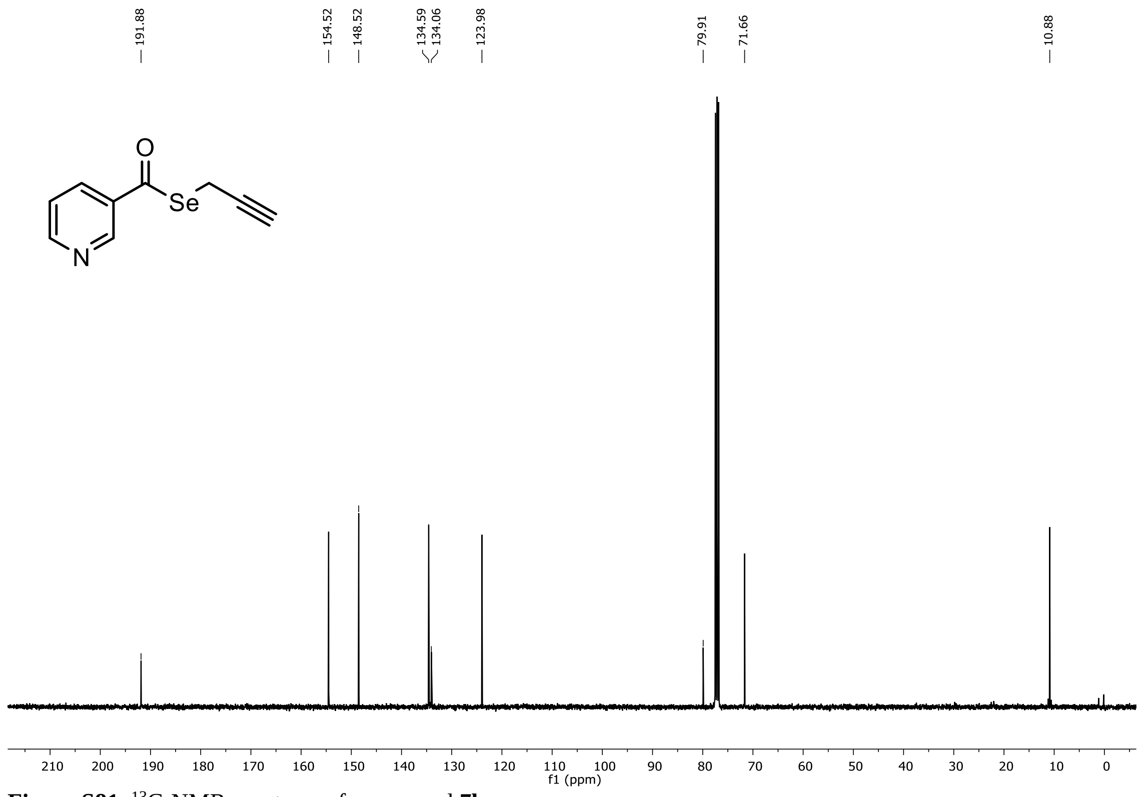

Figure S81. ^{13}C-NMR spectrum of compound **7b.**

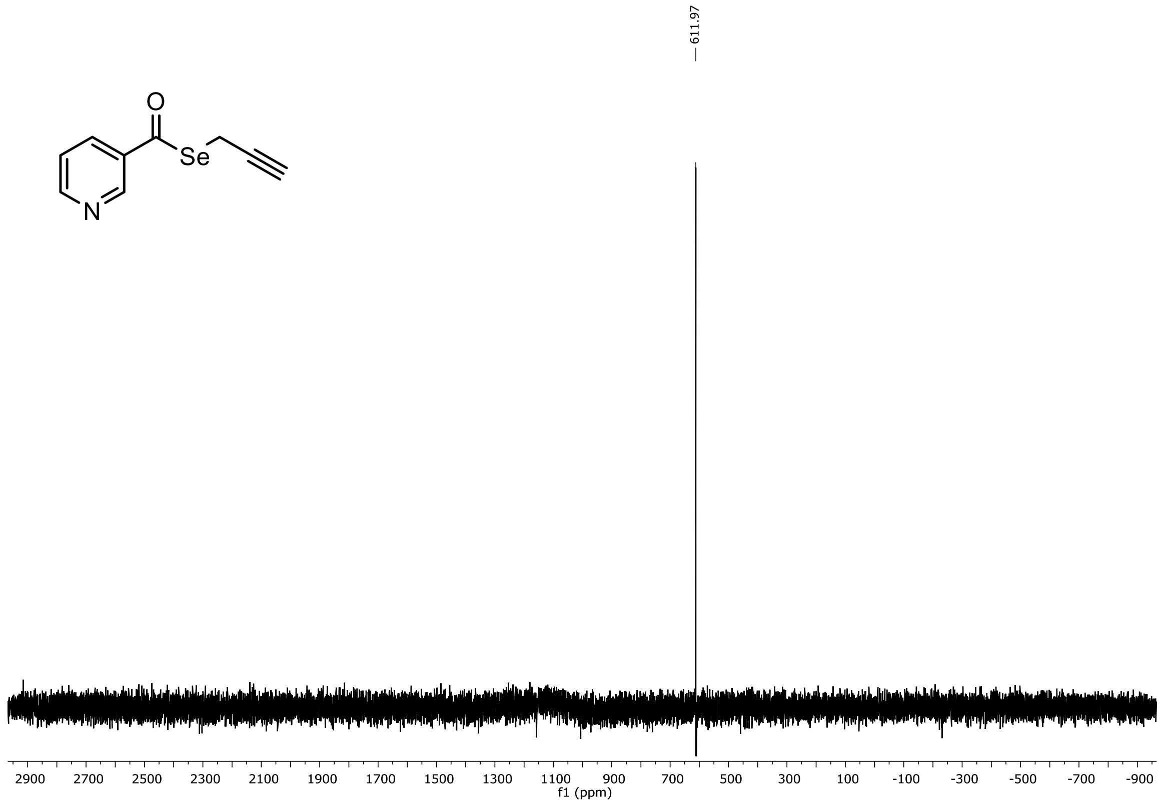

Figure S82. ^{77}Se-NMR spectrum of compound **7b.**

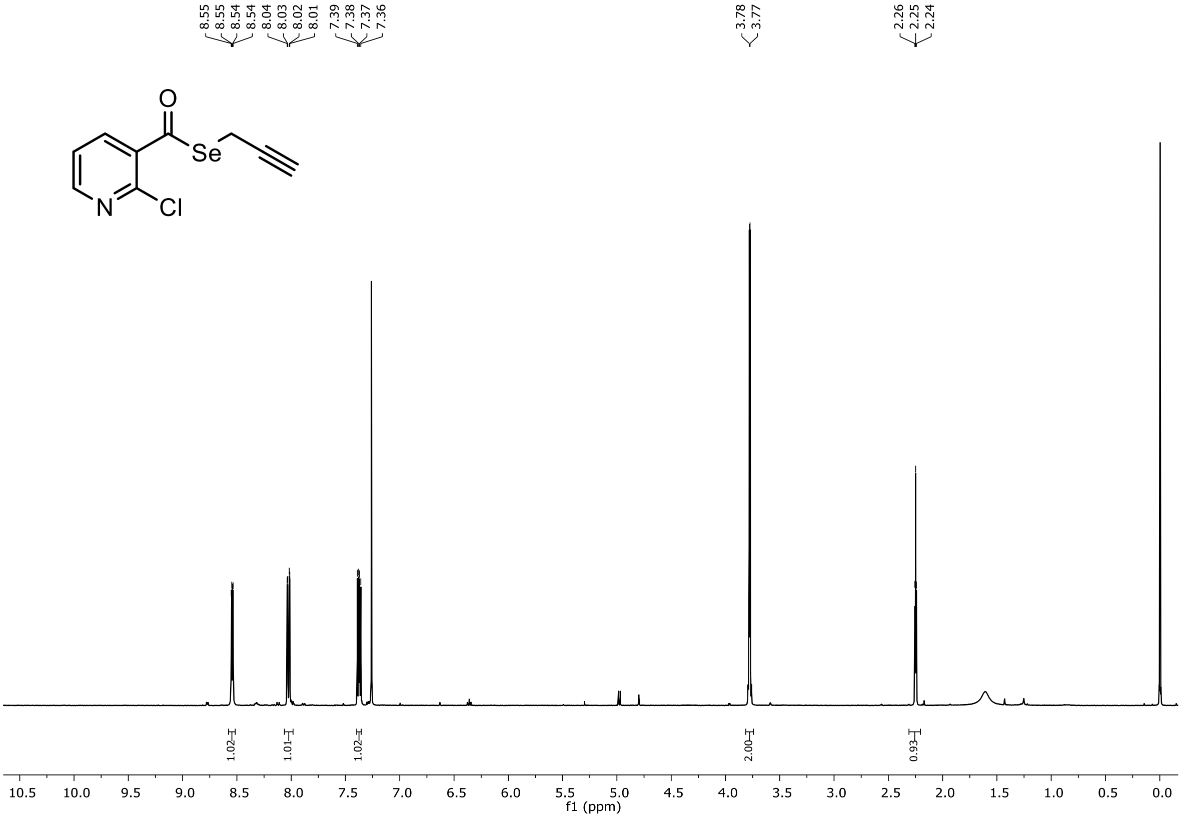

Figure S83. ^{1}H-NMR spectrum of compound **8b.**

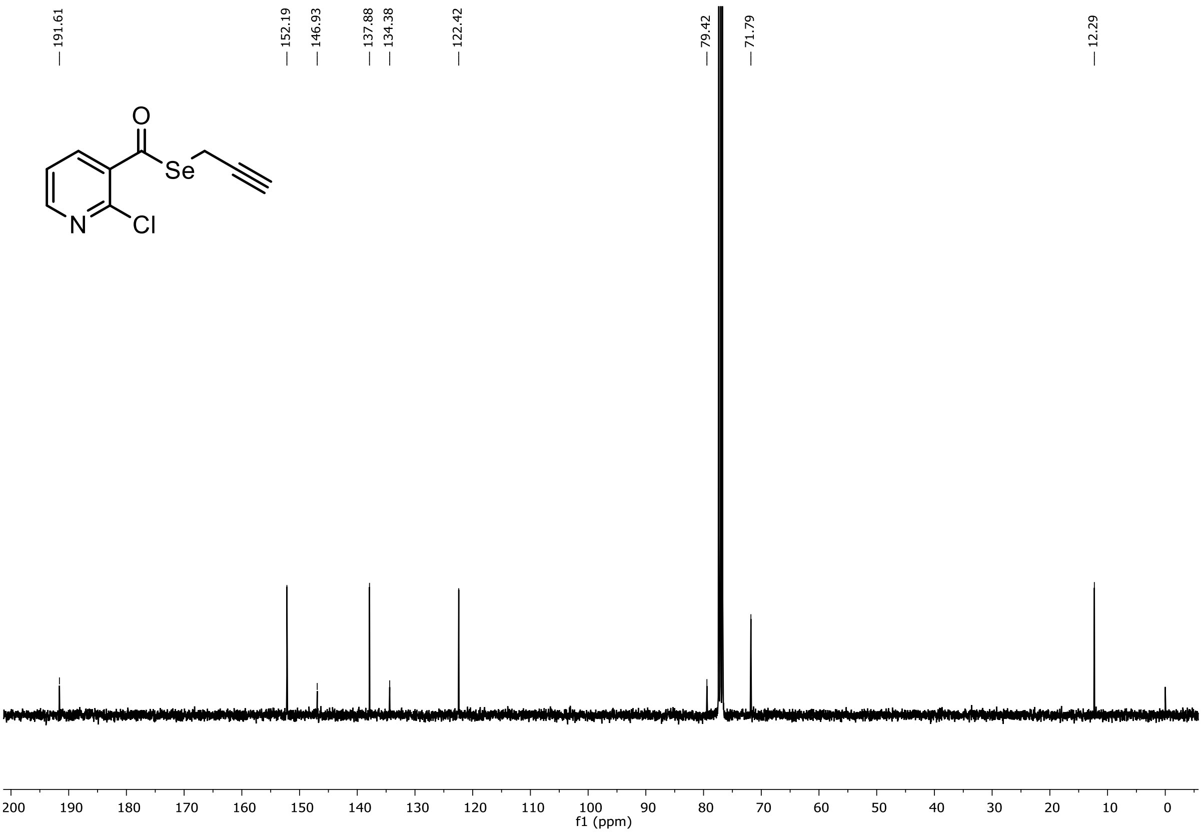

Figure S84. ^{13}C-NMR spectrum of compound **8b.**

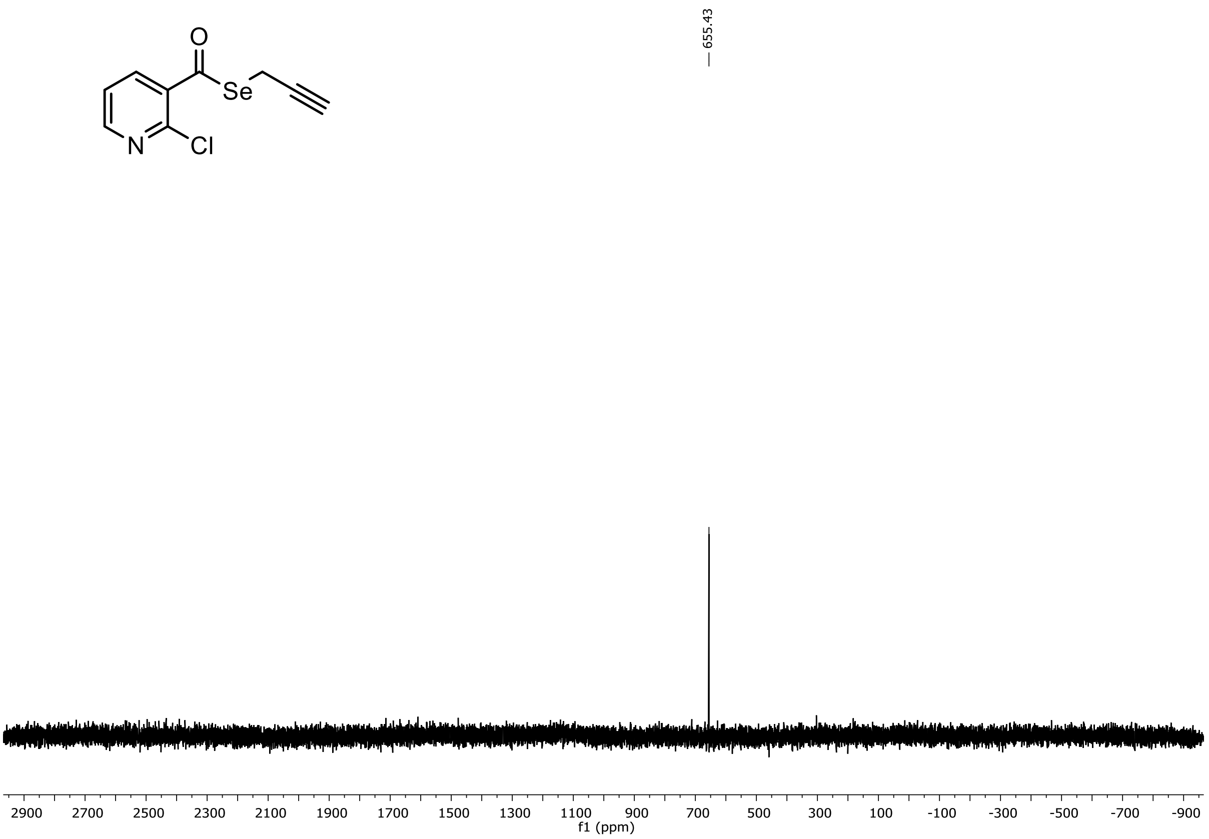

Figure S85. ^{77}Se-NMR spectrum of compound **8b.**

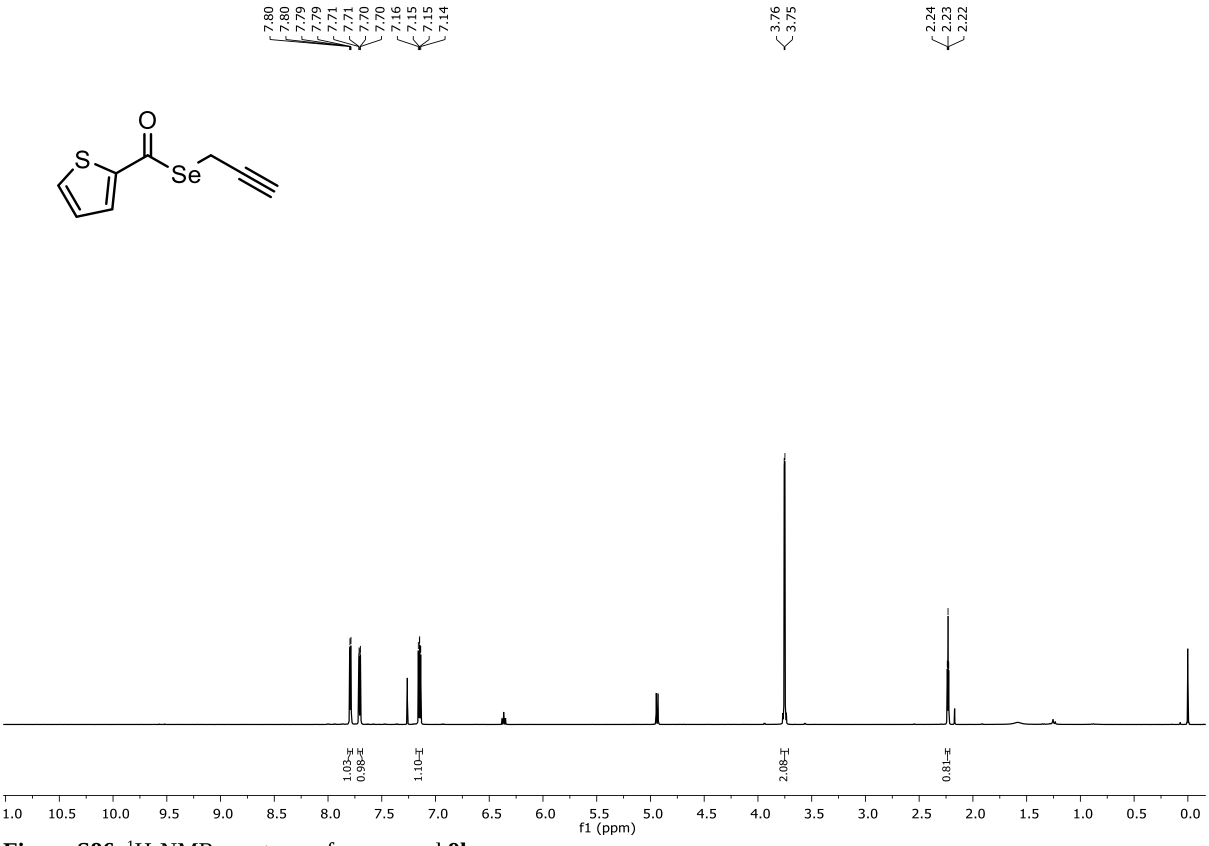

Figure S86. ^{1}H-NMR spectrum of compound **9b.**

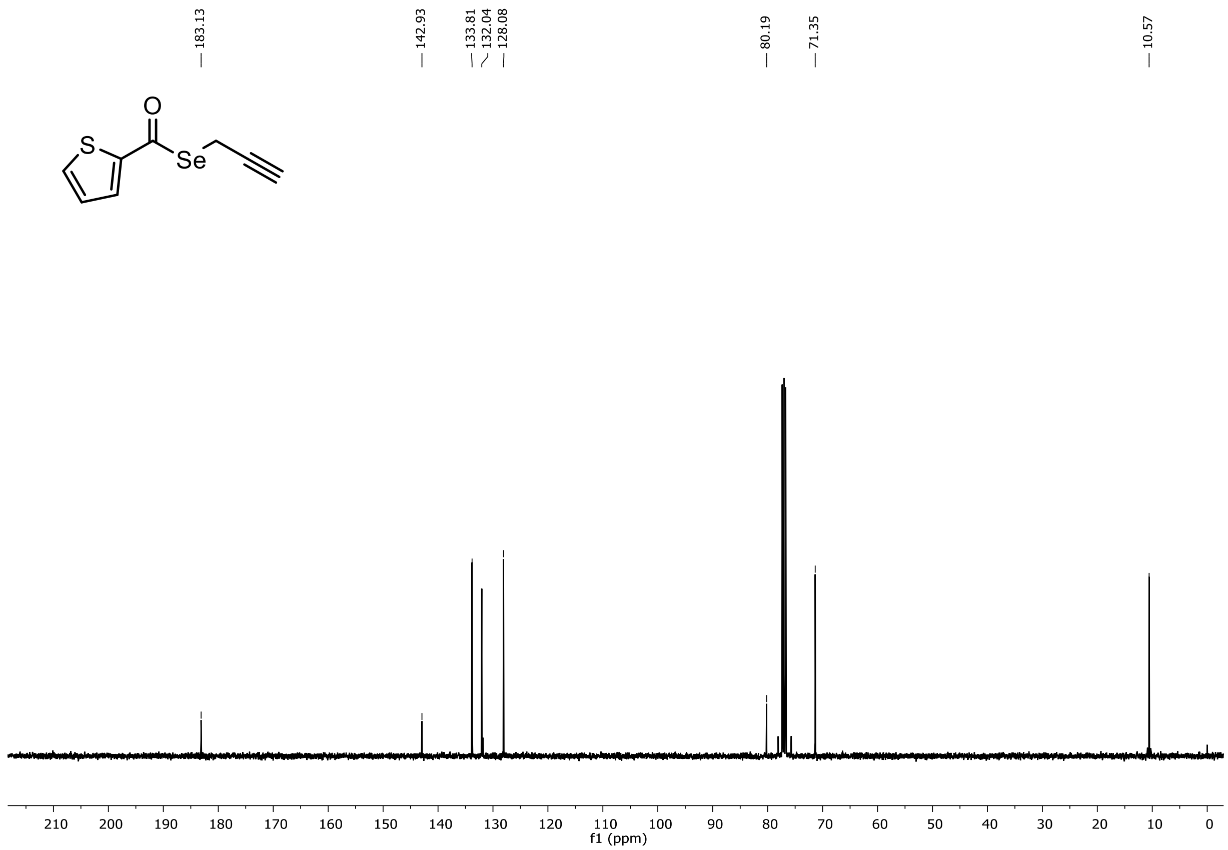

Figure S87. ^{13}C-NMR spectrum of compound **9b.**

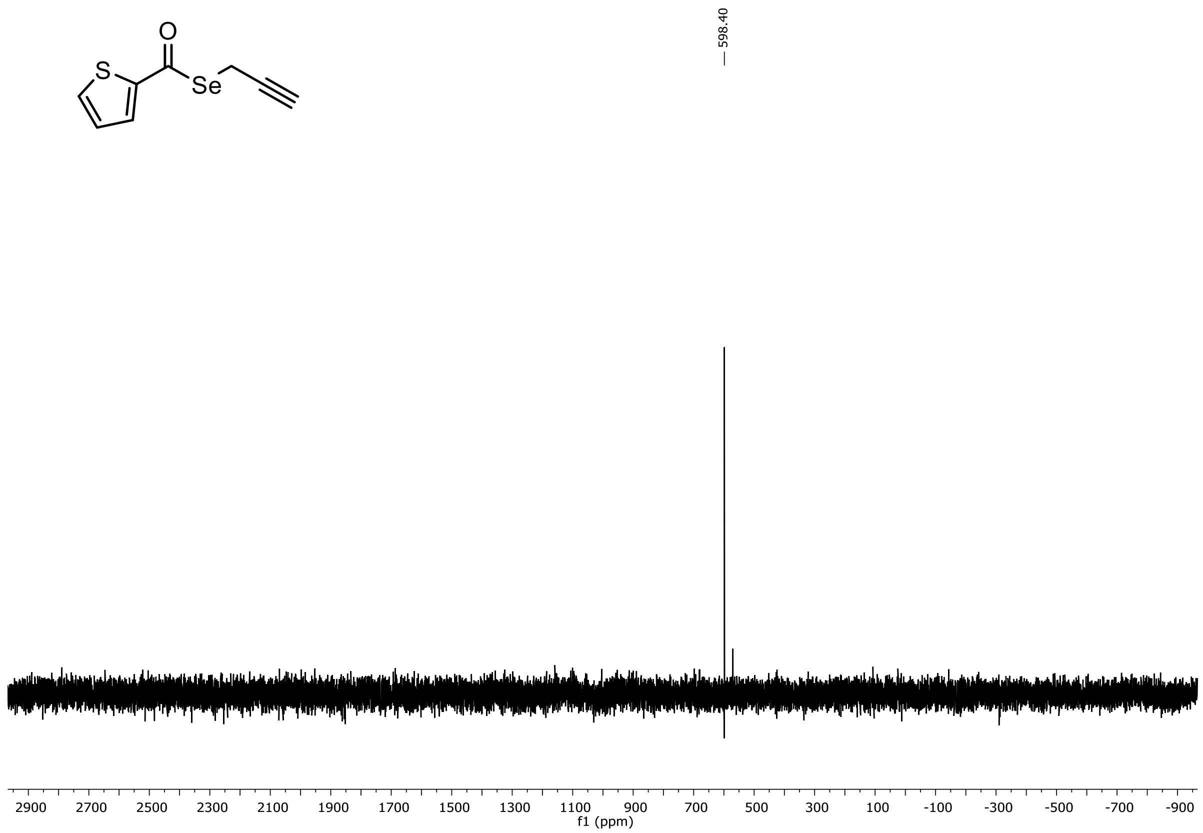

Figure S88. ^{77}Se-NMR spectrum of compound **9b.**

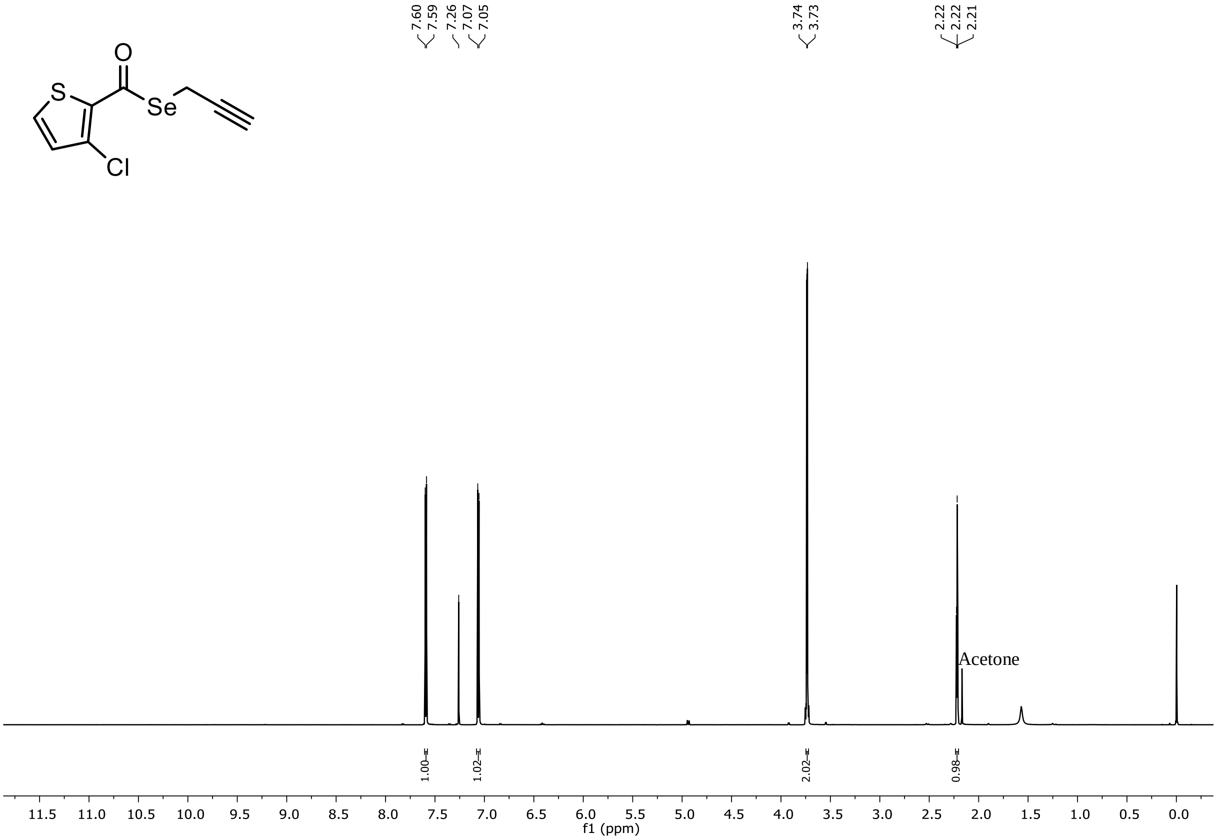

Figure S89. ^{1}H-NMR spectrum of compound **10b.**

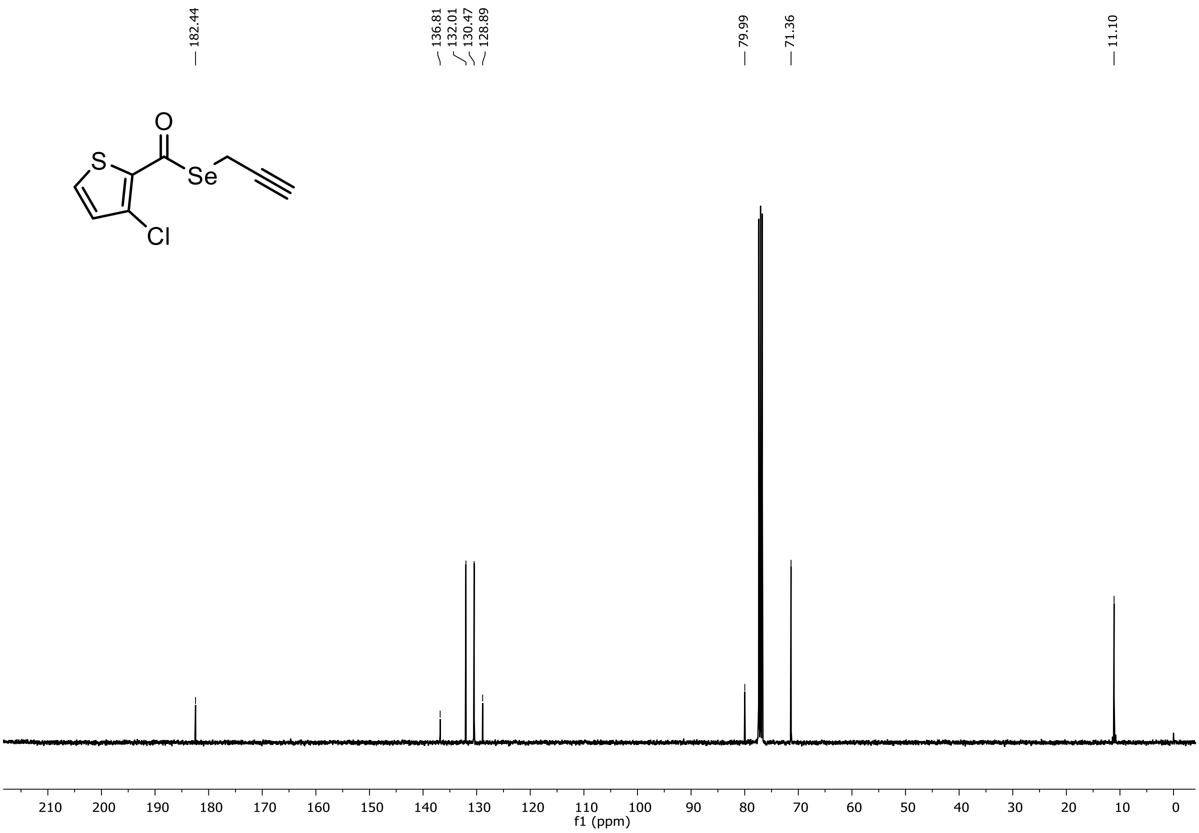

Figure S90. ^{13}C-NMR spectrum of compound **10b.**

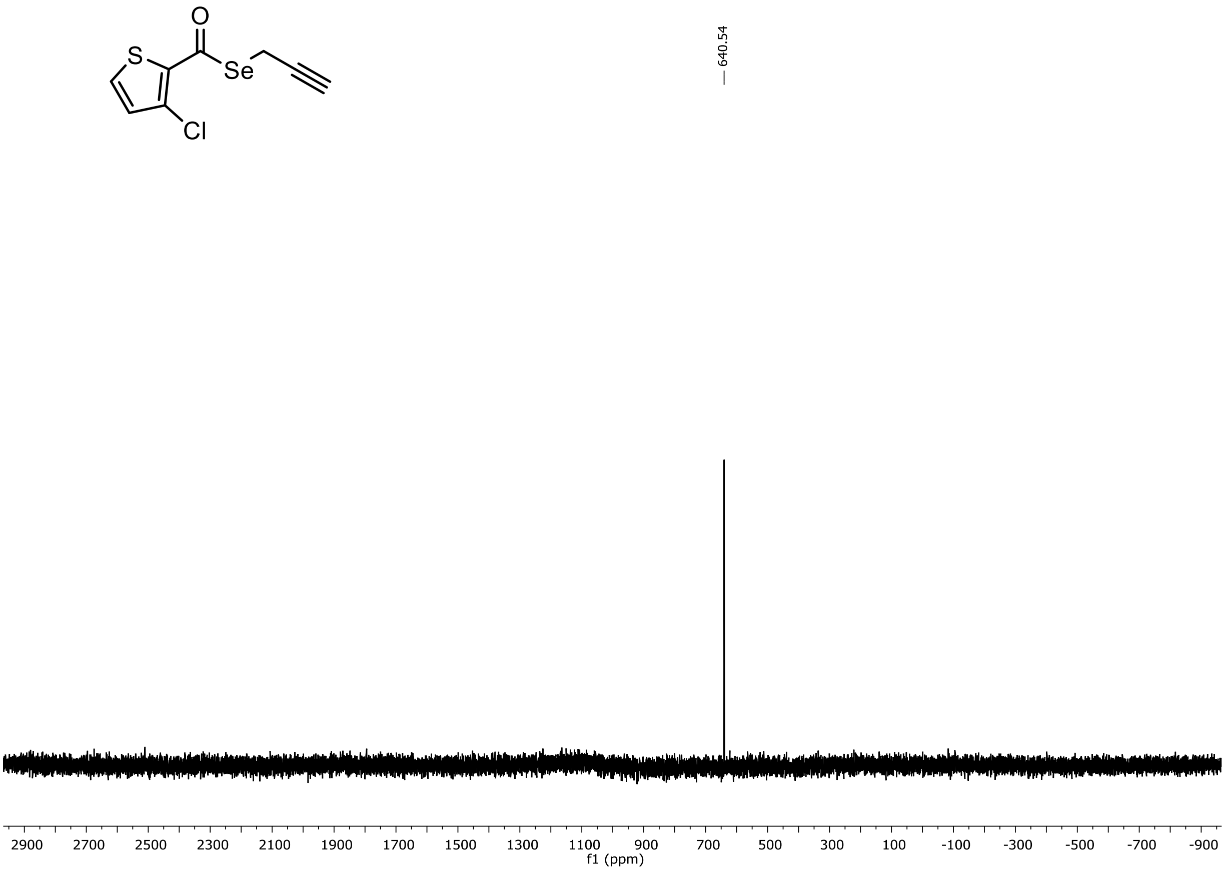

Figure S91. ^{77}Se-NMR spectrum of compound **10b.**

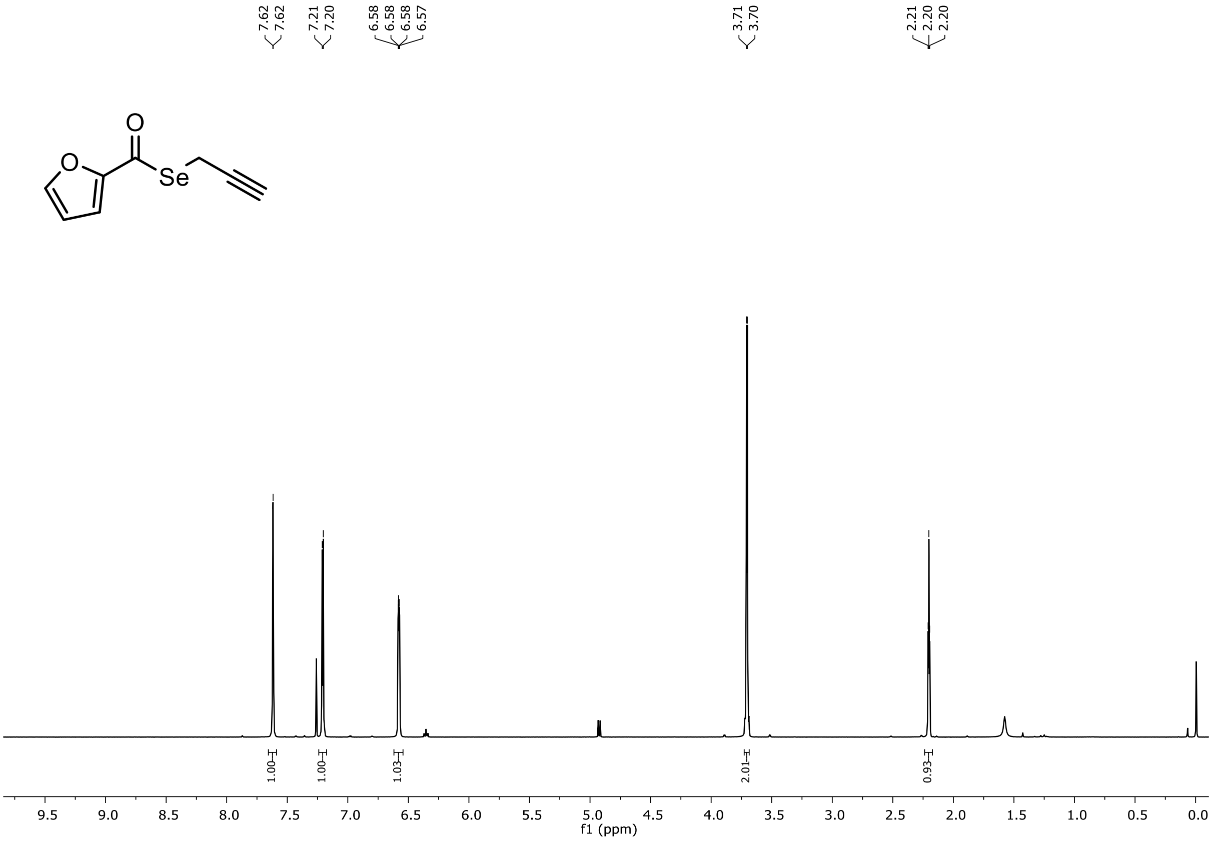

Figure S92. ^{1}H-NMR spectrum of compound **11b.**

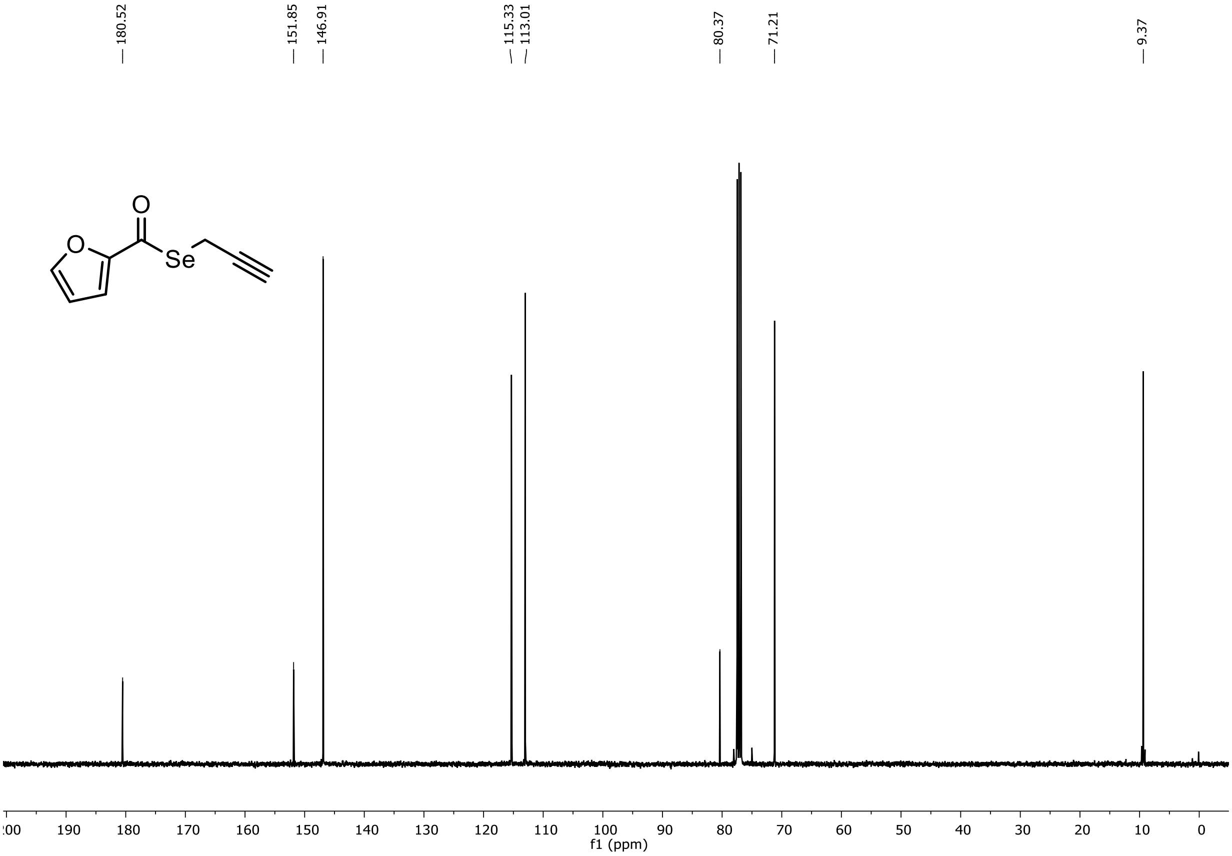

Figure S93. ^{13}C-NMR spectrum of compound **11b.**

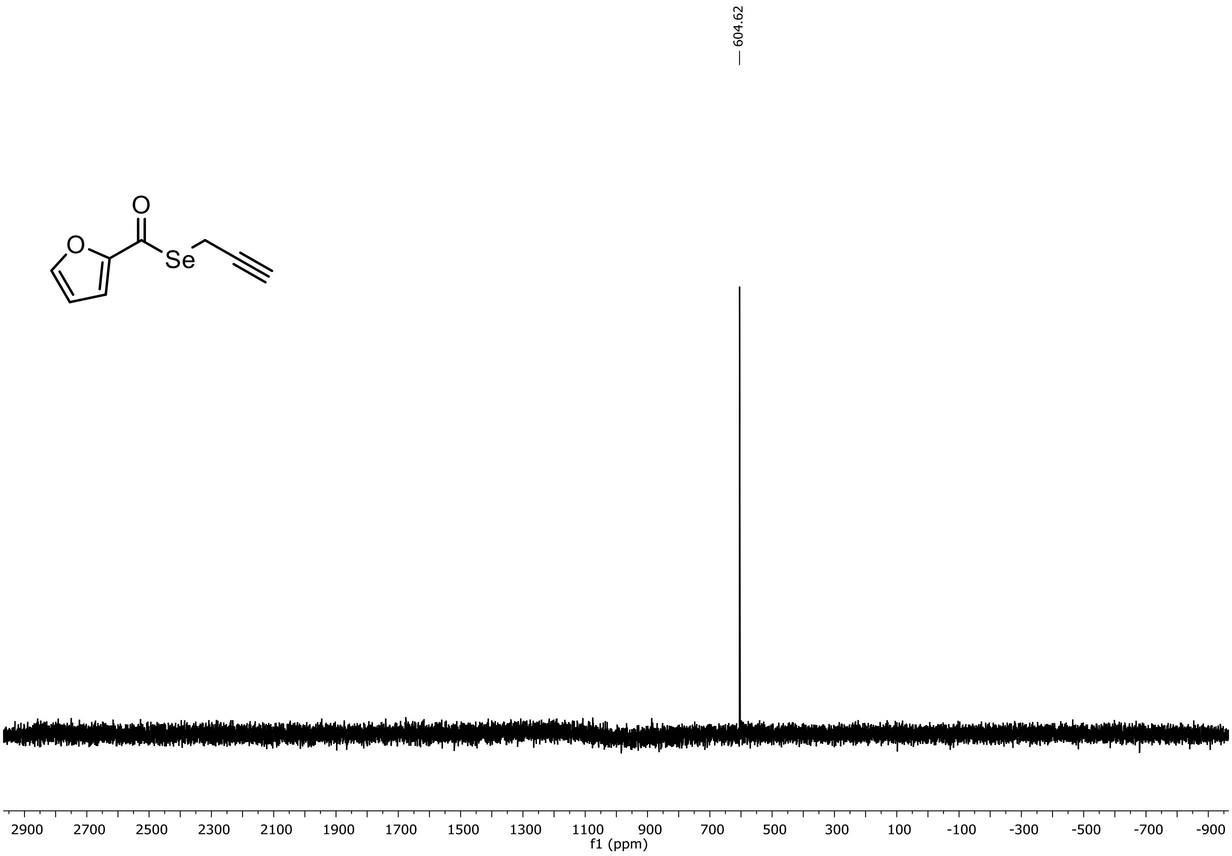

Figure S94. ^{77}Se-NMR spectrum of compound **11b.**

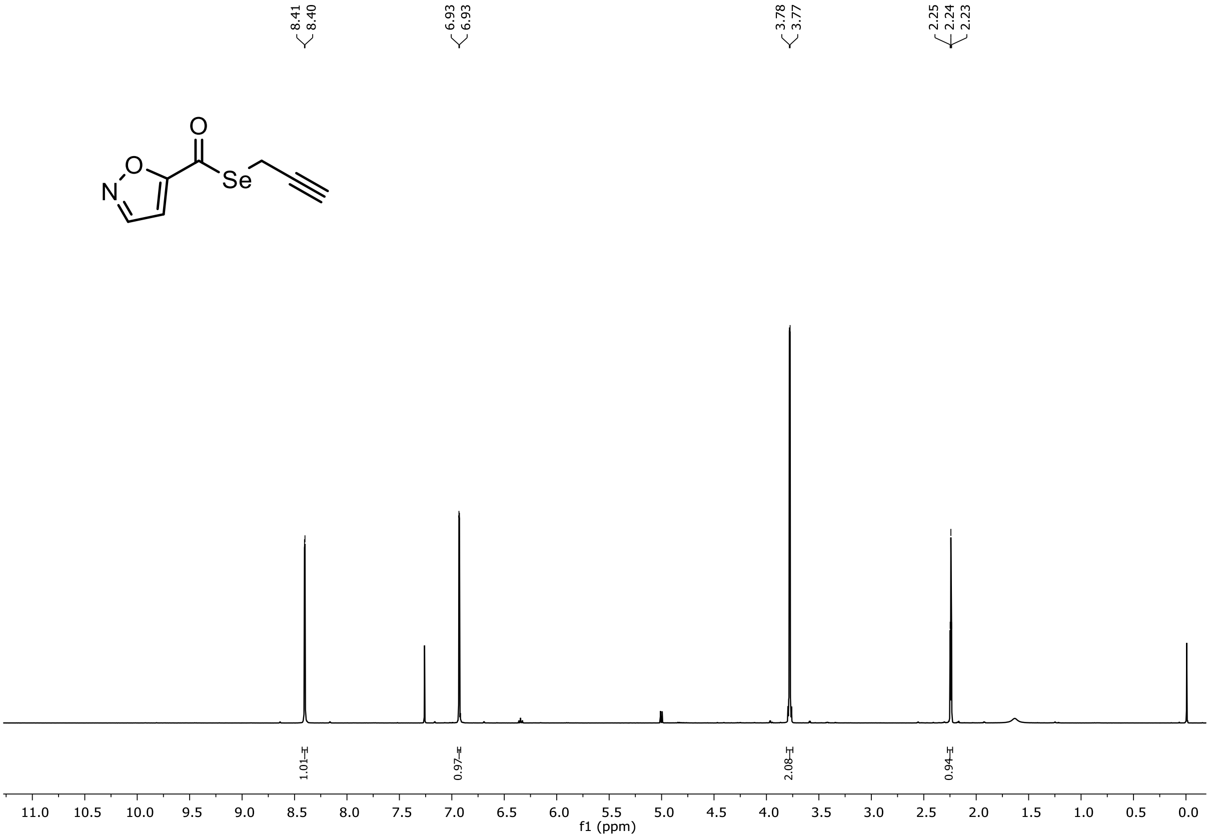

Figure S95. ^{1}H-NMR spectrum of compound **12b.**

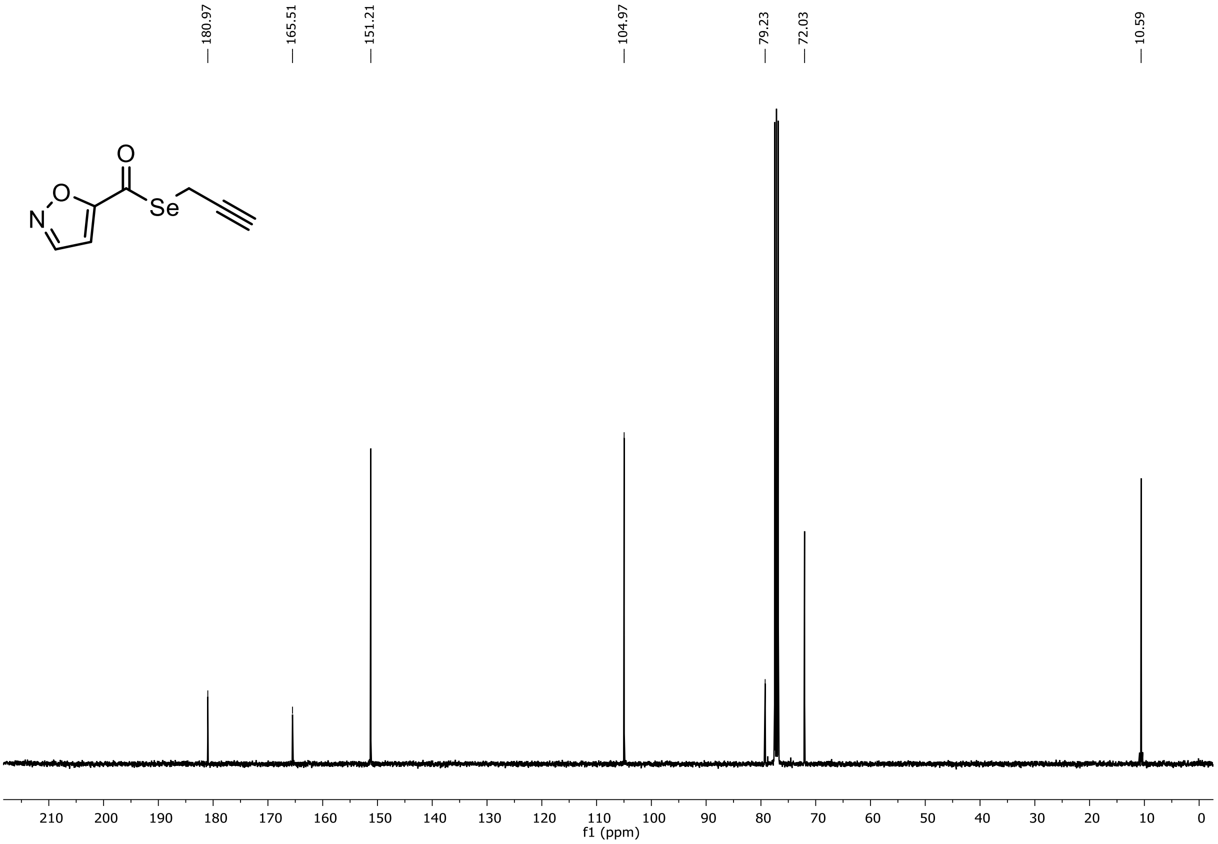

Figure S96. ^{13}C-NMR spectrum of compound **12b.**

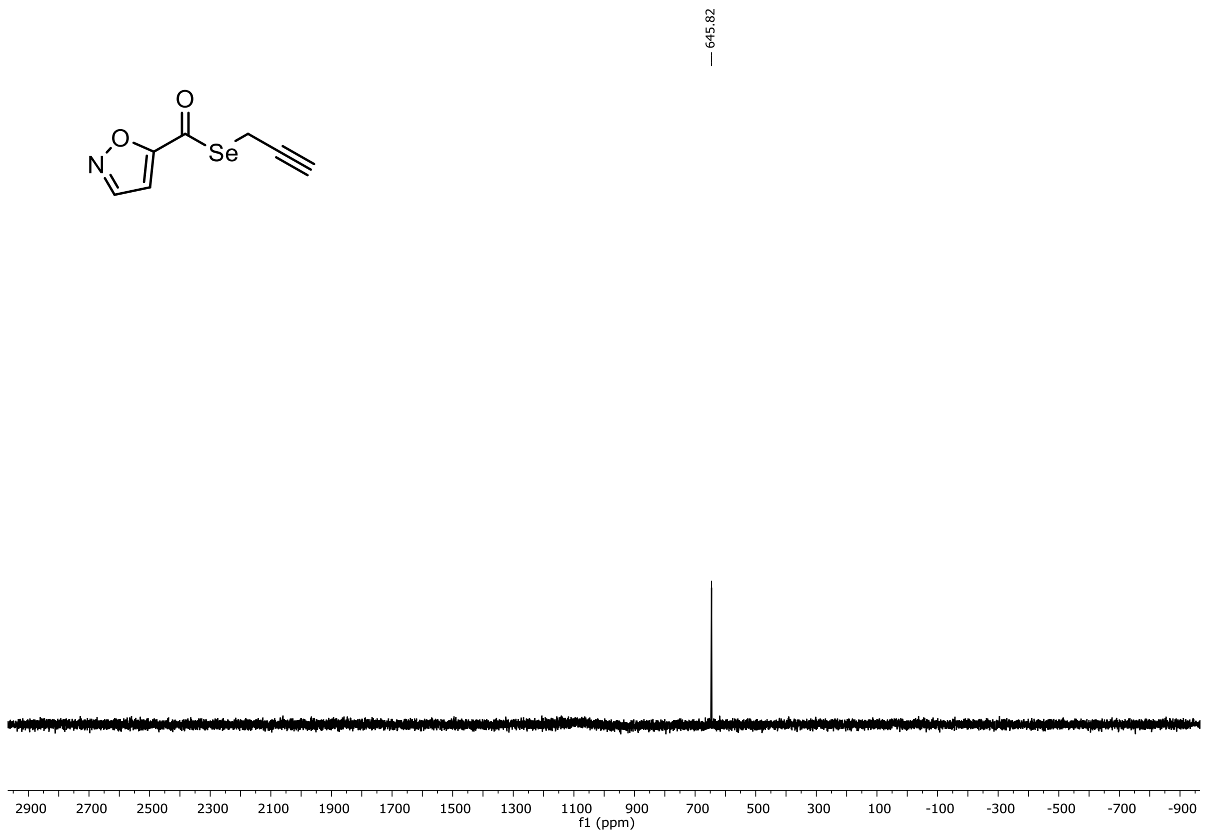

Figure S97. ^{77}Se-NMR spectrum of compound **12b.**

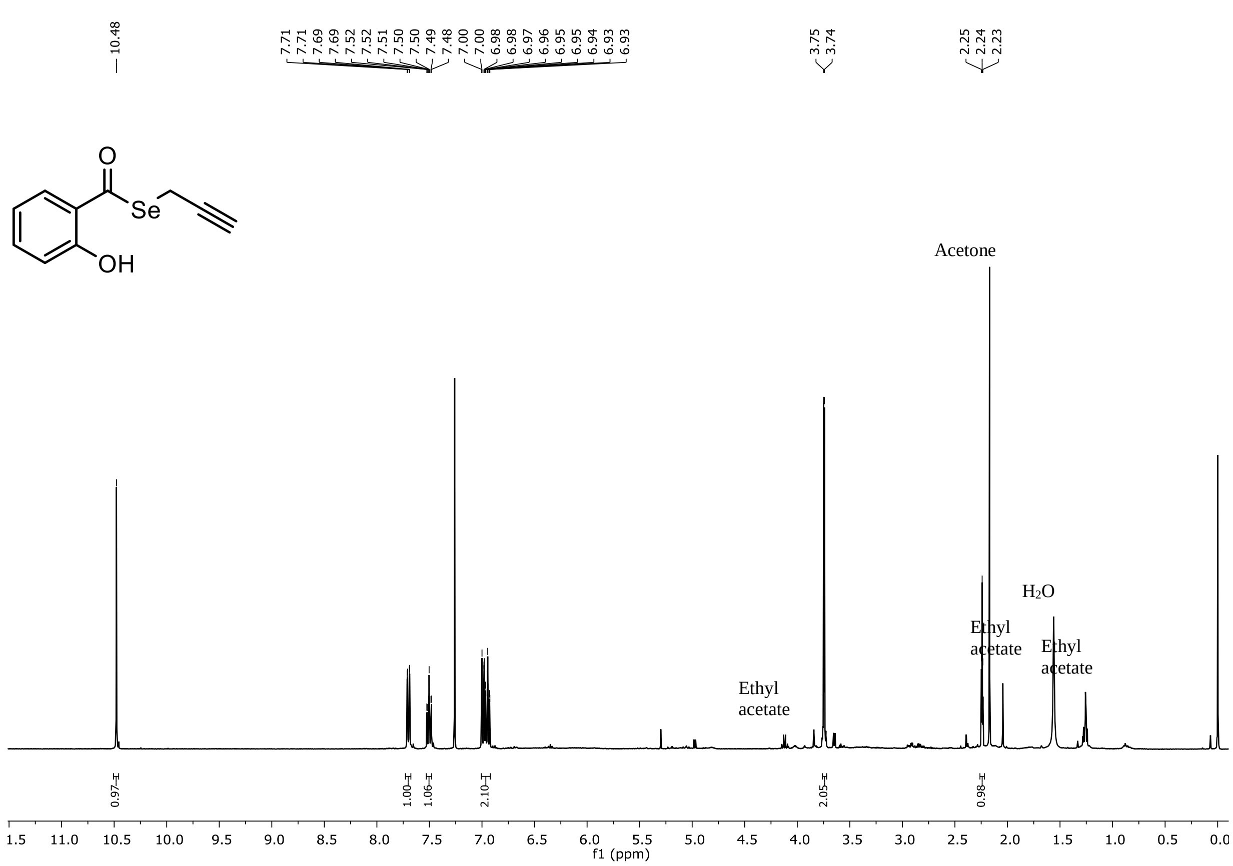

Figure S98. ^{1}H-NMR spectrum of compound **13b.**

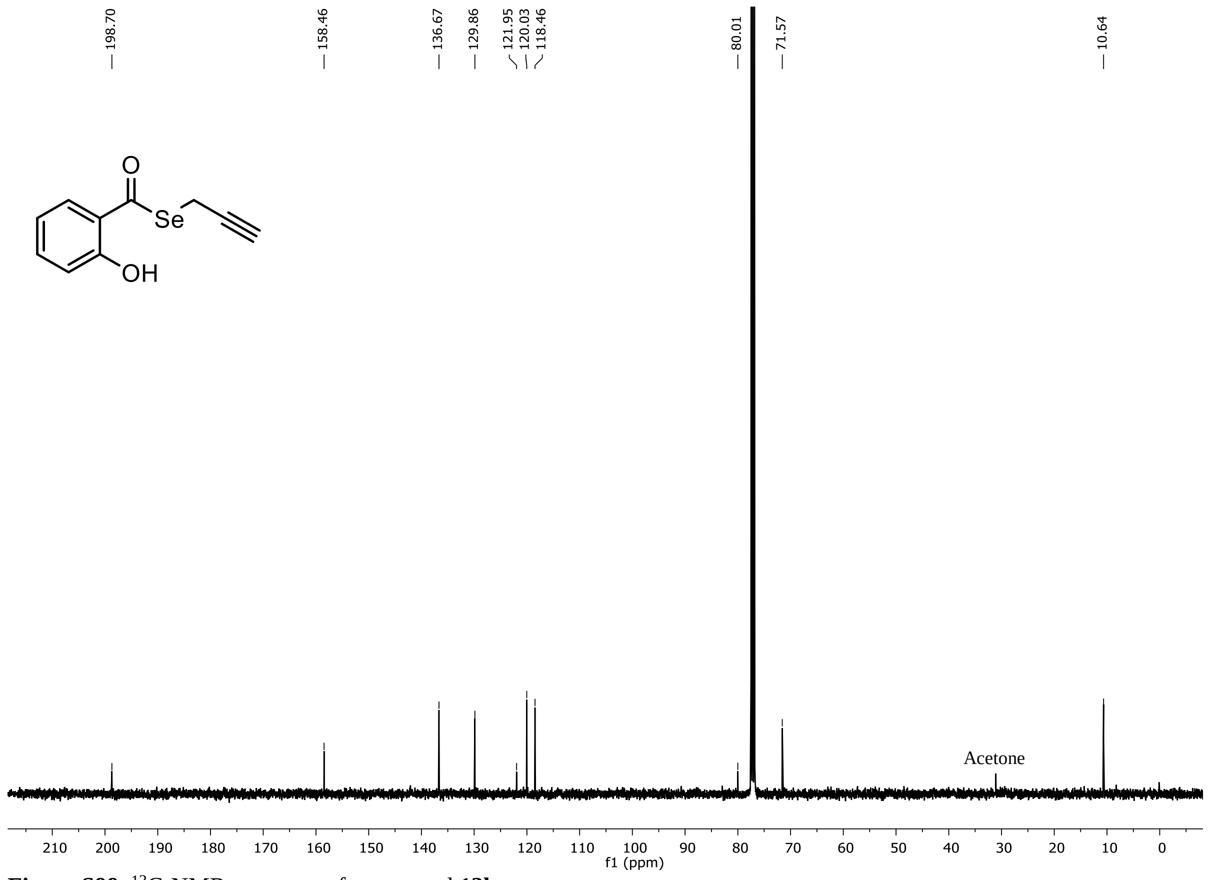

Figure S99. ^{13}C-NMR spectrum of compound **13b.**

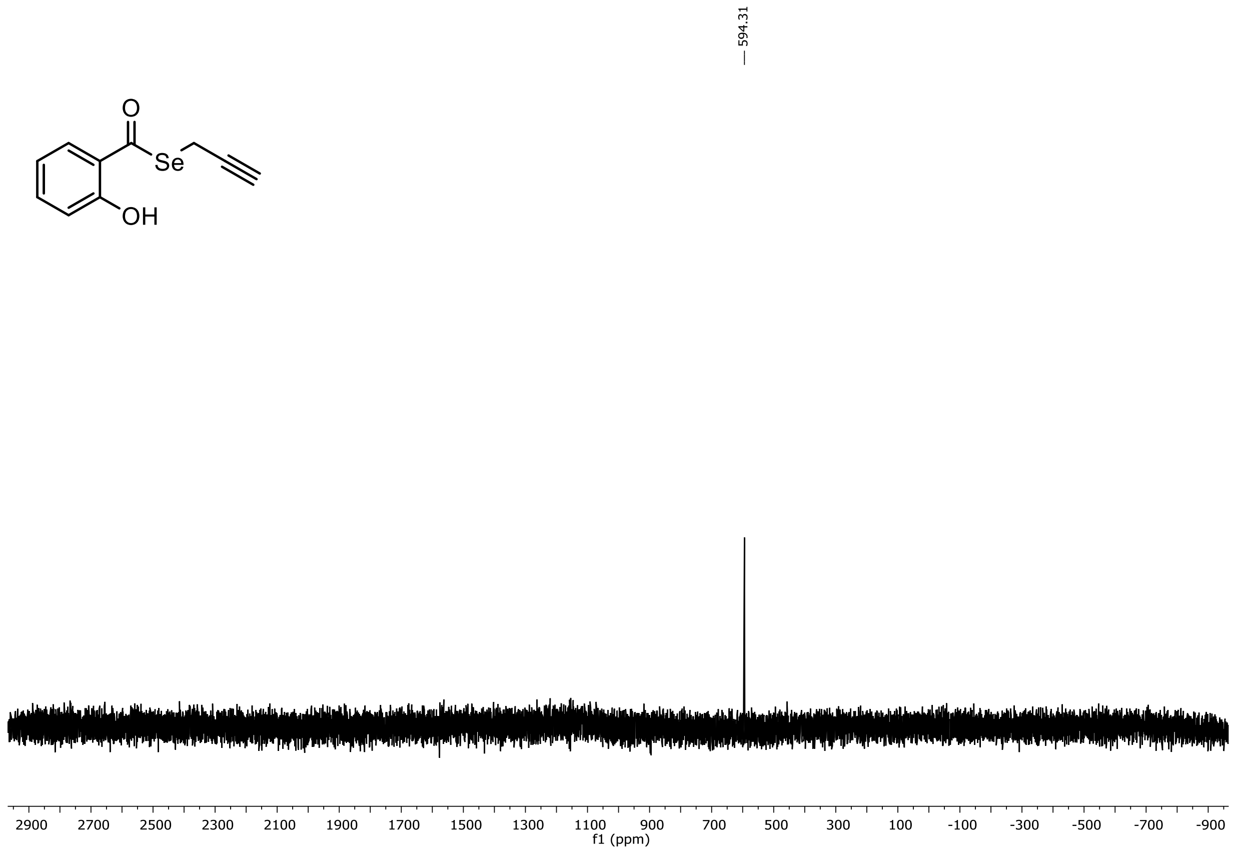

Figure S100. ^{77}Se-NMR spectrum of compound **13b.**

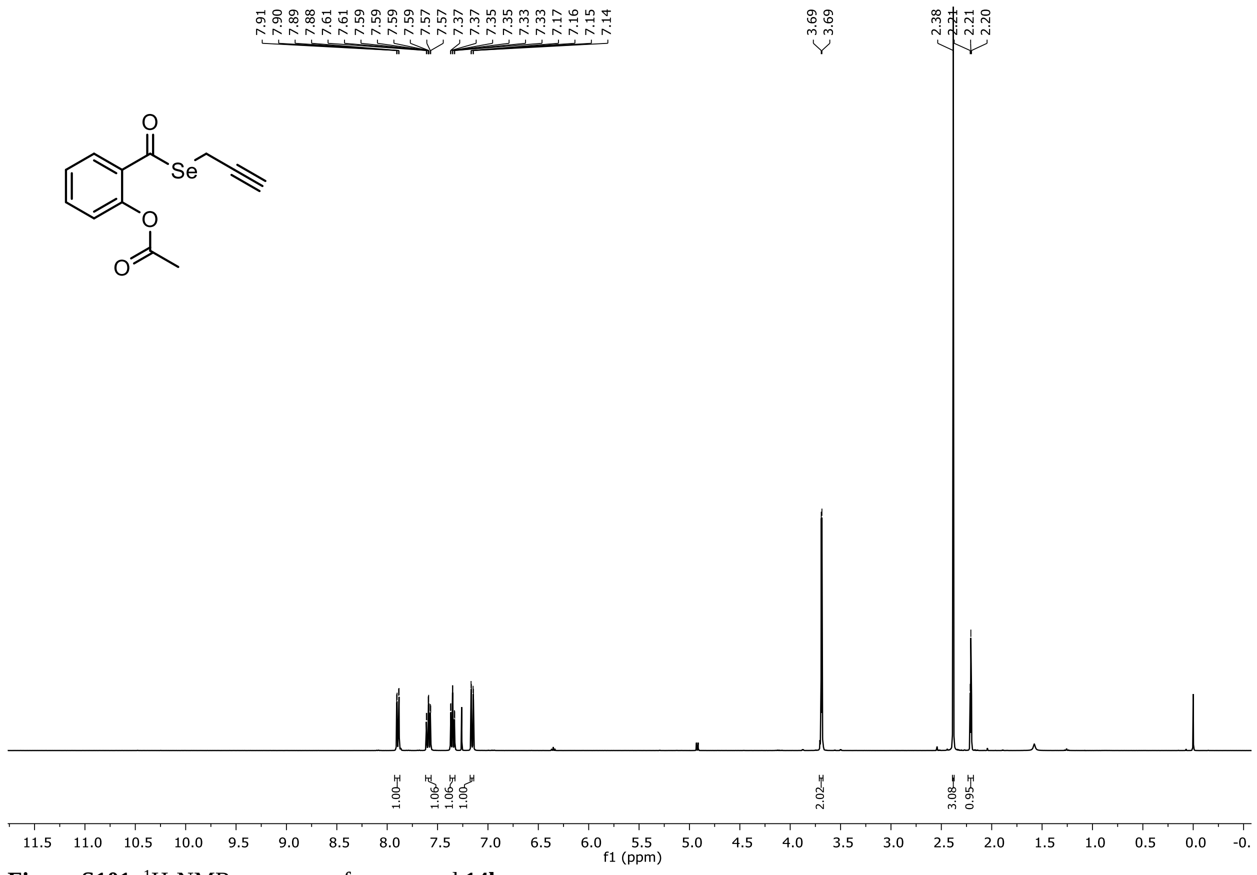

Figure S101. ^{1}H-NMR spectrum of compound **14b.**

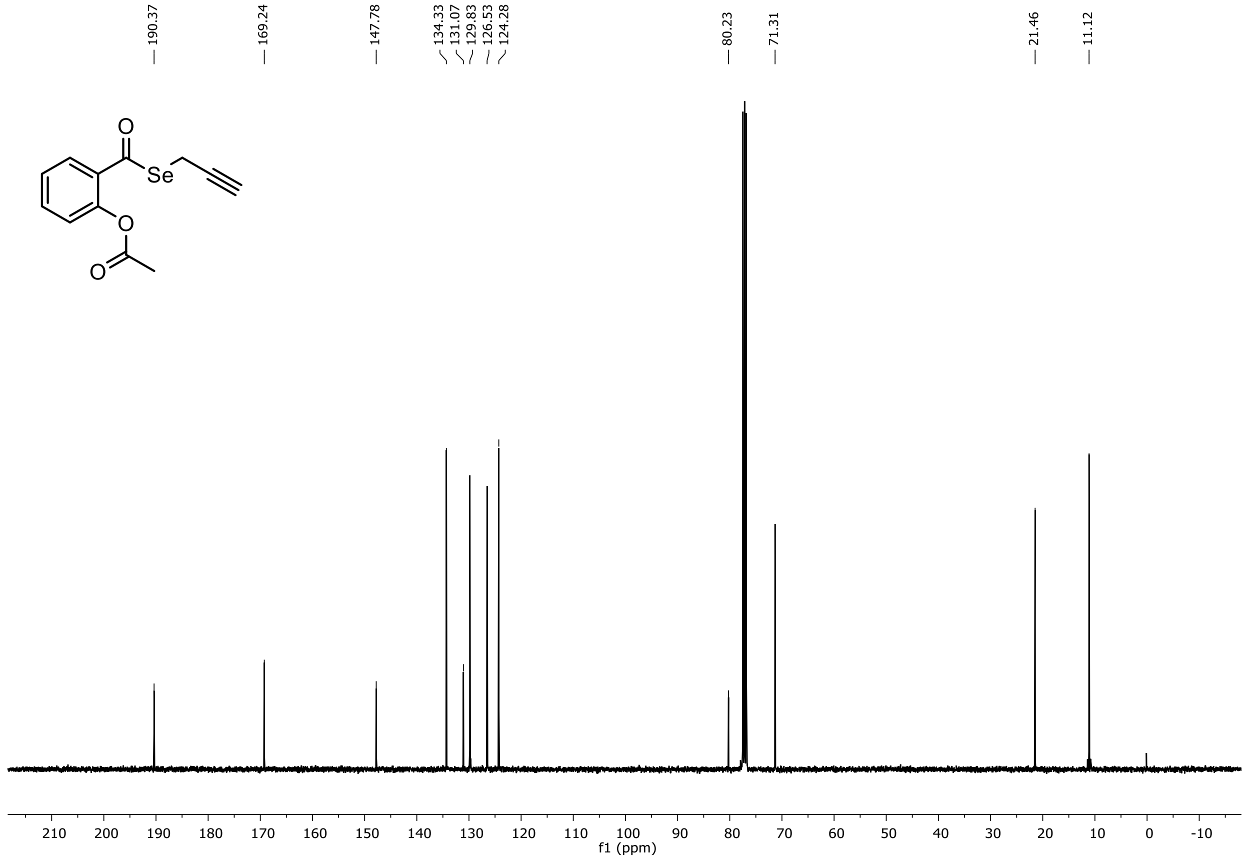

Figure S102. ^{13}C-NMR spectrum of compound **14b.**

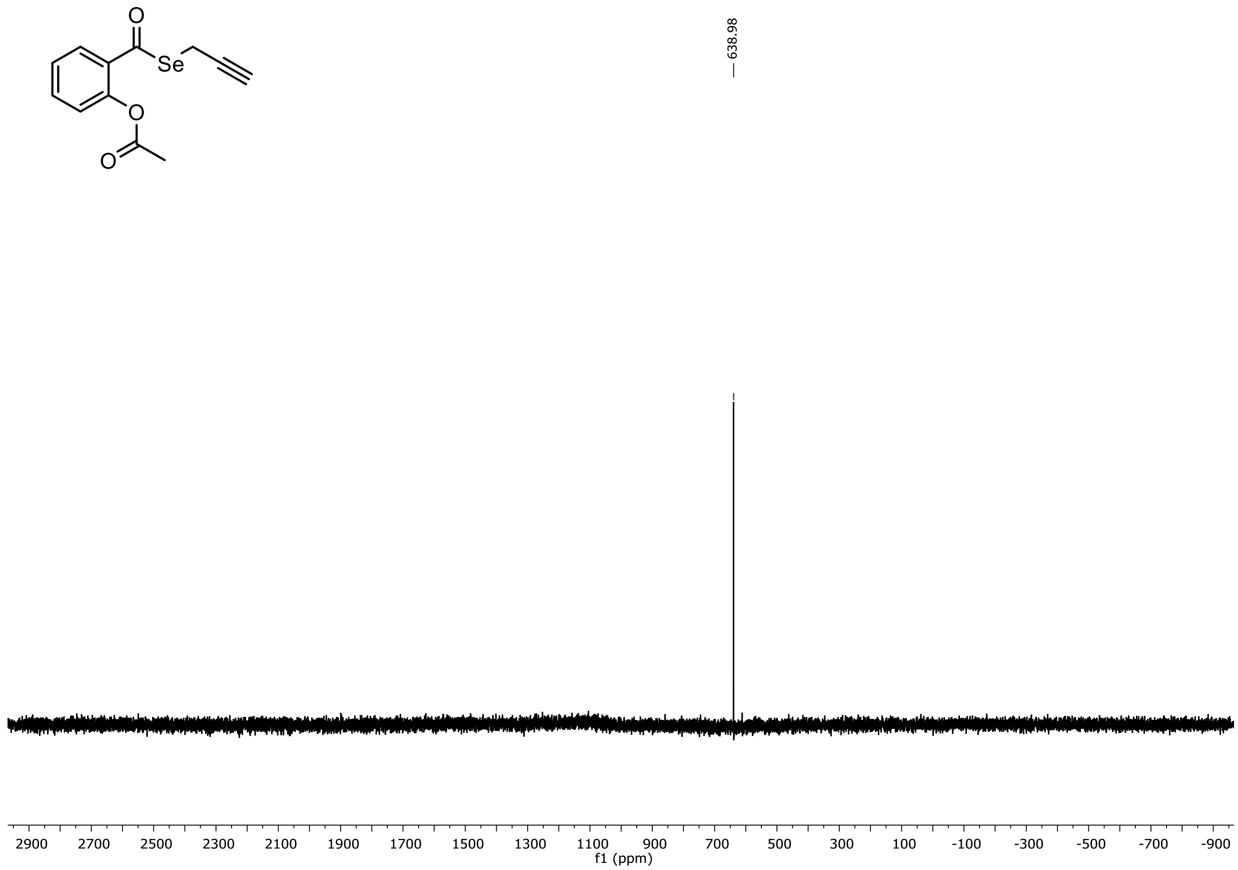

Figure S103. ^{77}Se-NMR spectrum of compound **14b.**

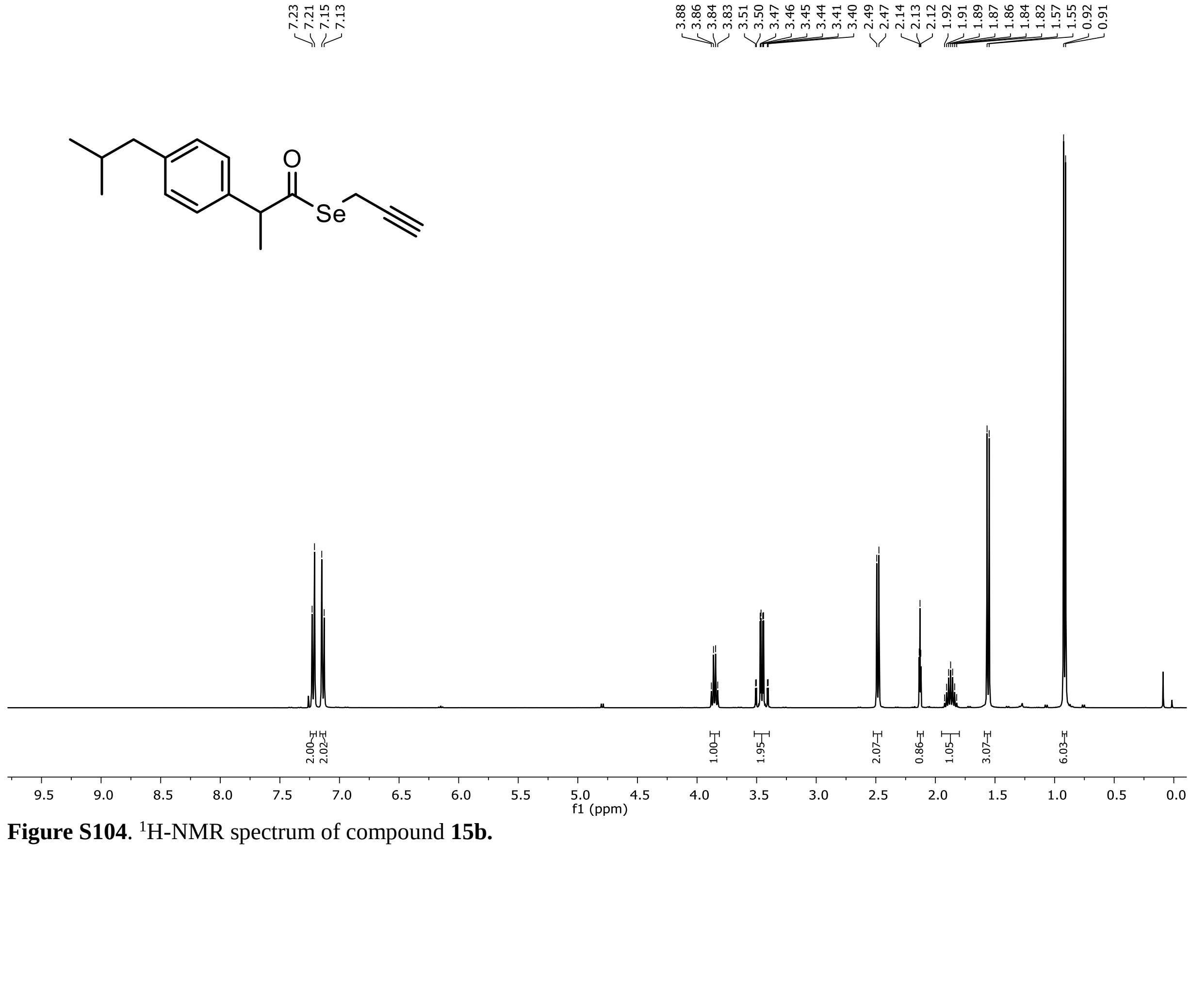

Figure S104. ^{1}H-NMR spectrum of compound **15b.**

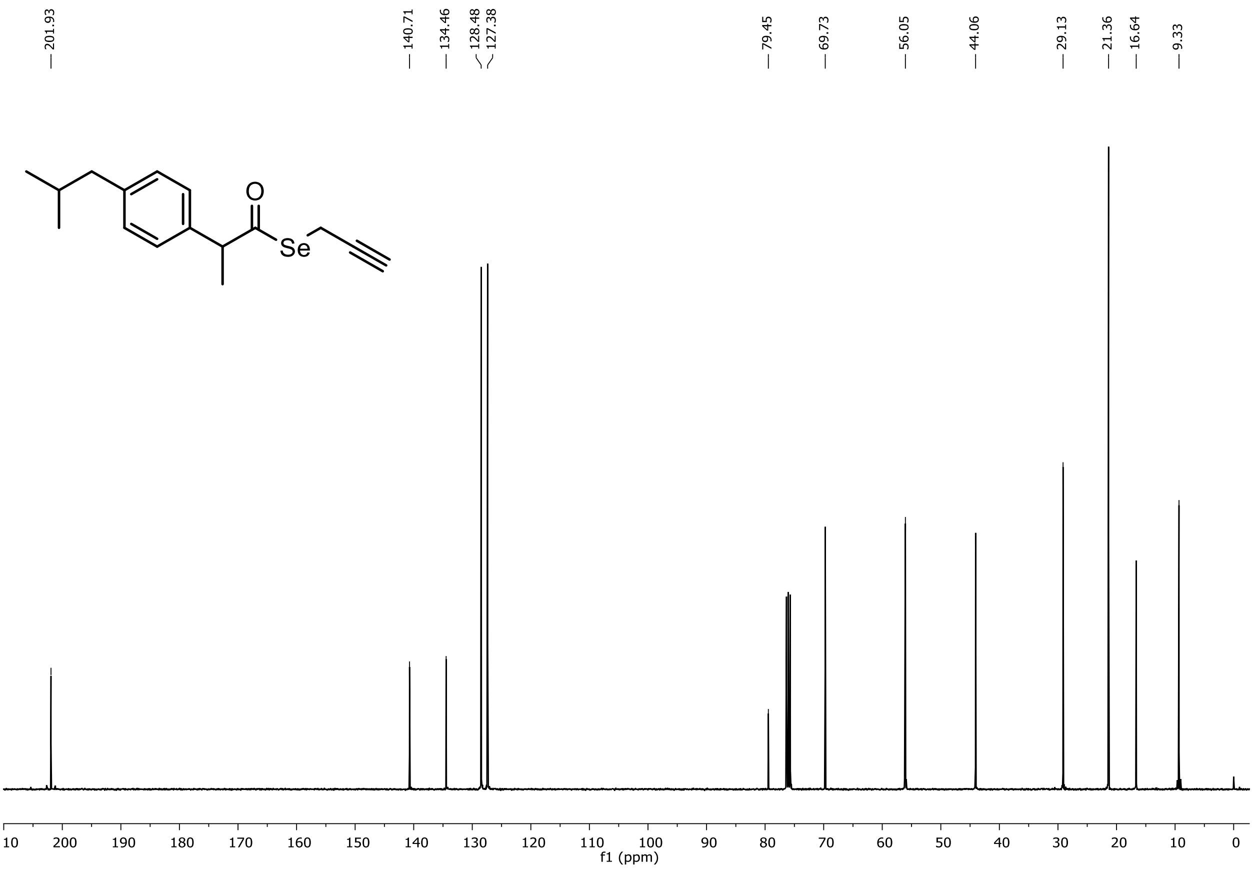

Figure S105. ^{13}C-NMR spectrum of compound **15b.**

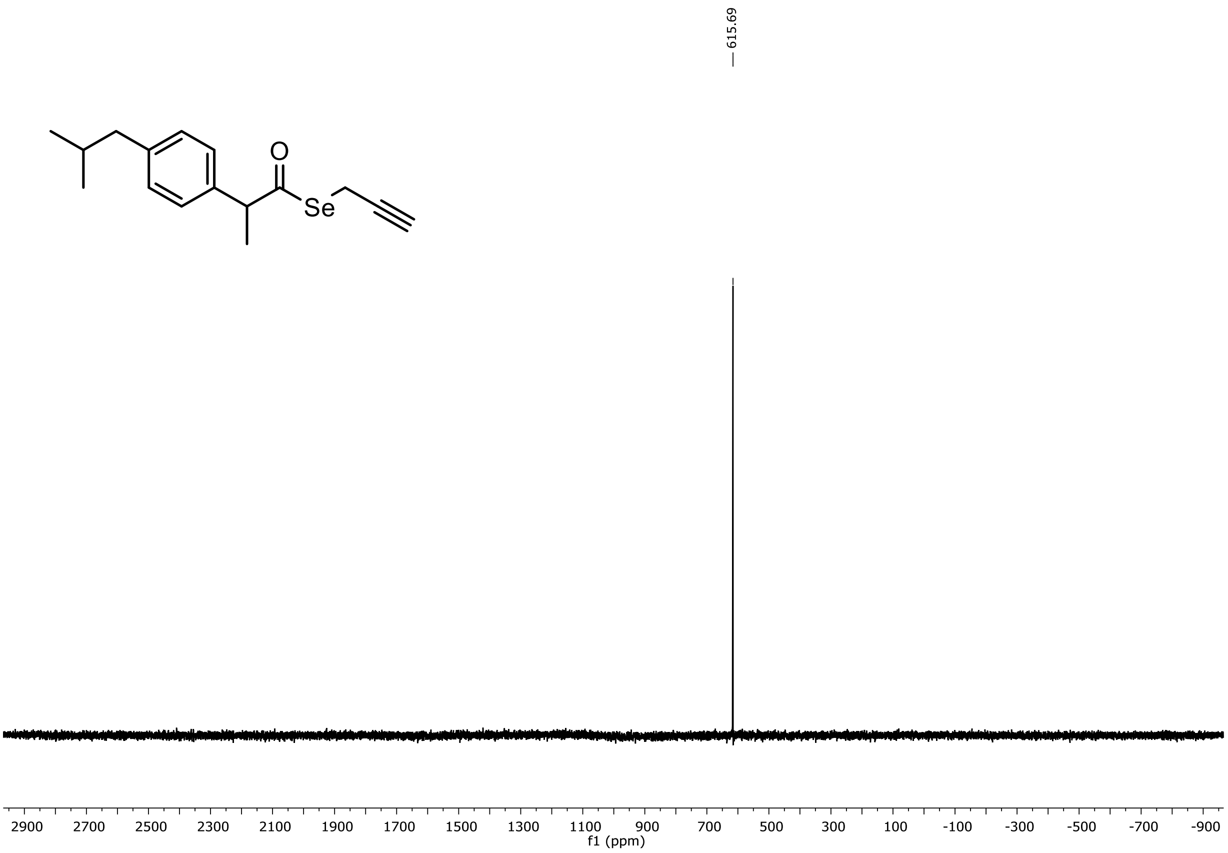

Figure S106. ^{77}Se-NMR spectrum of compound **15b.**

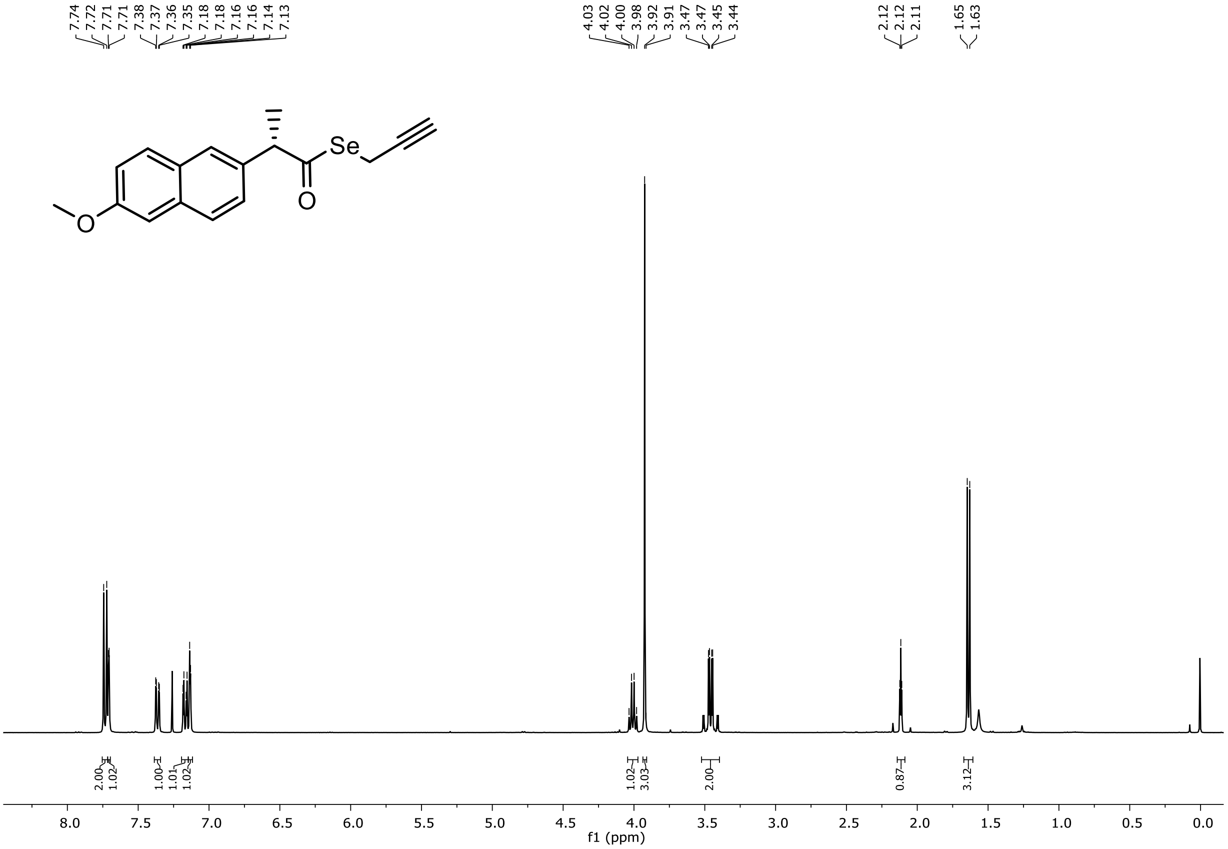

Figure S107. ^{1}H-NMR spectrum of compound **16b.**

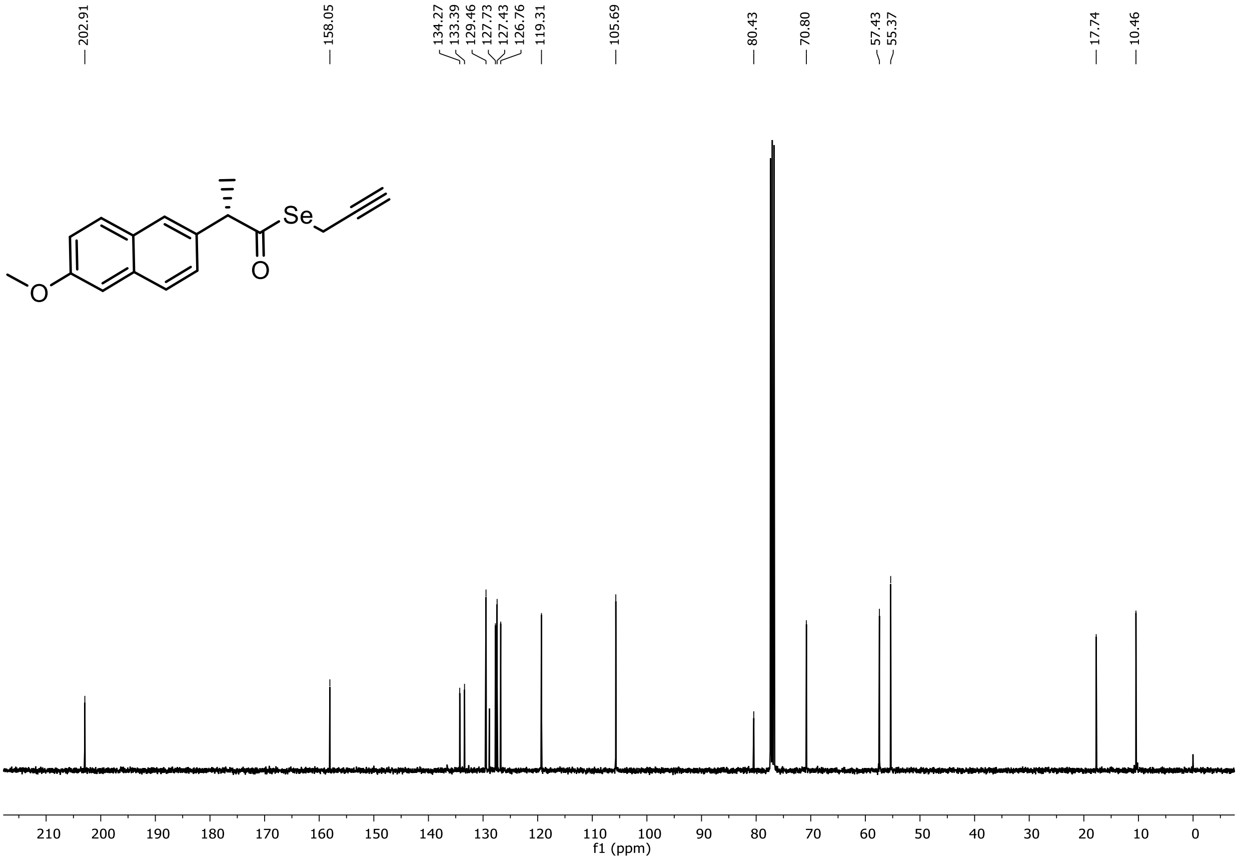

Figure S108. ^{13}C-NMR spectrum of compound **16b.**

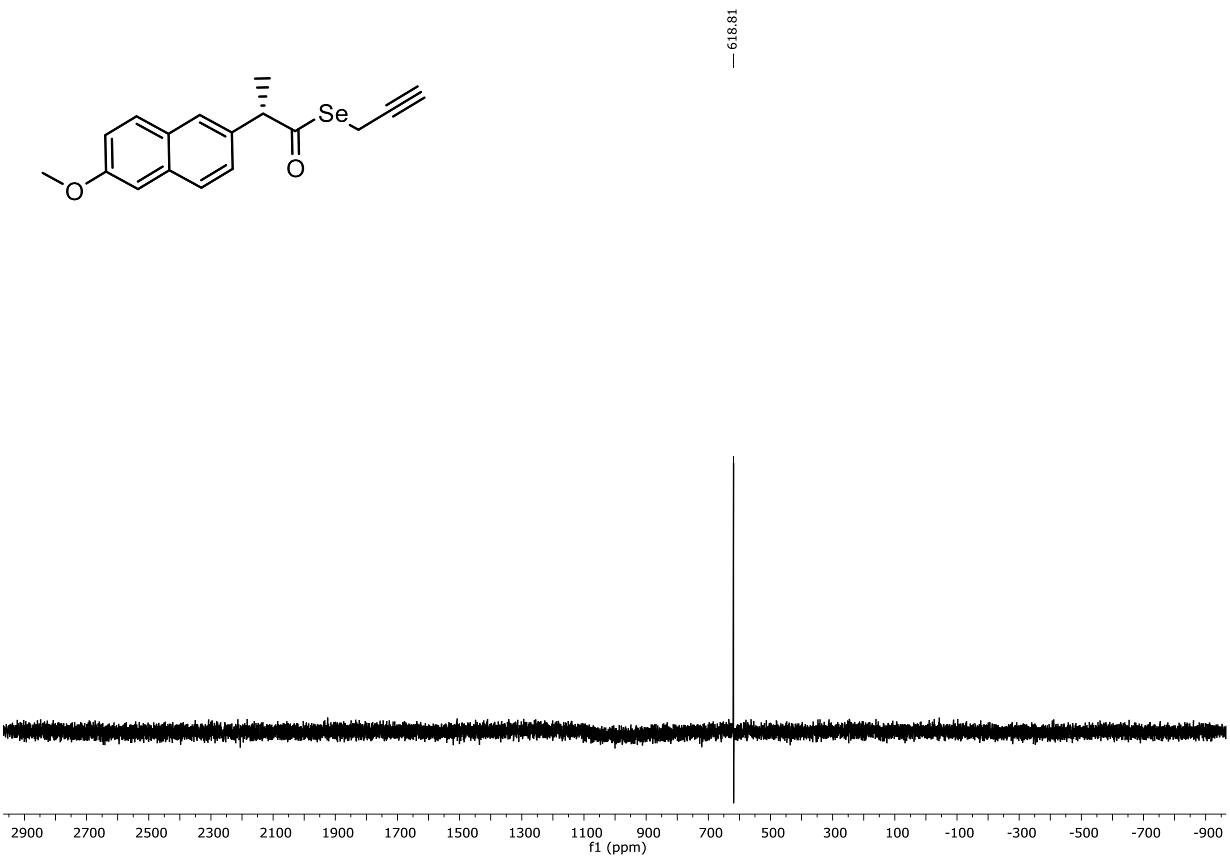

Figure S110. ^{77}Se-NMR spectrum of compound **16b.**

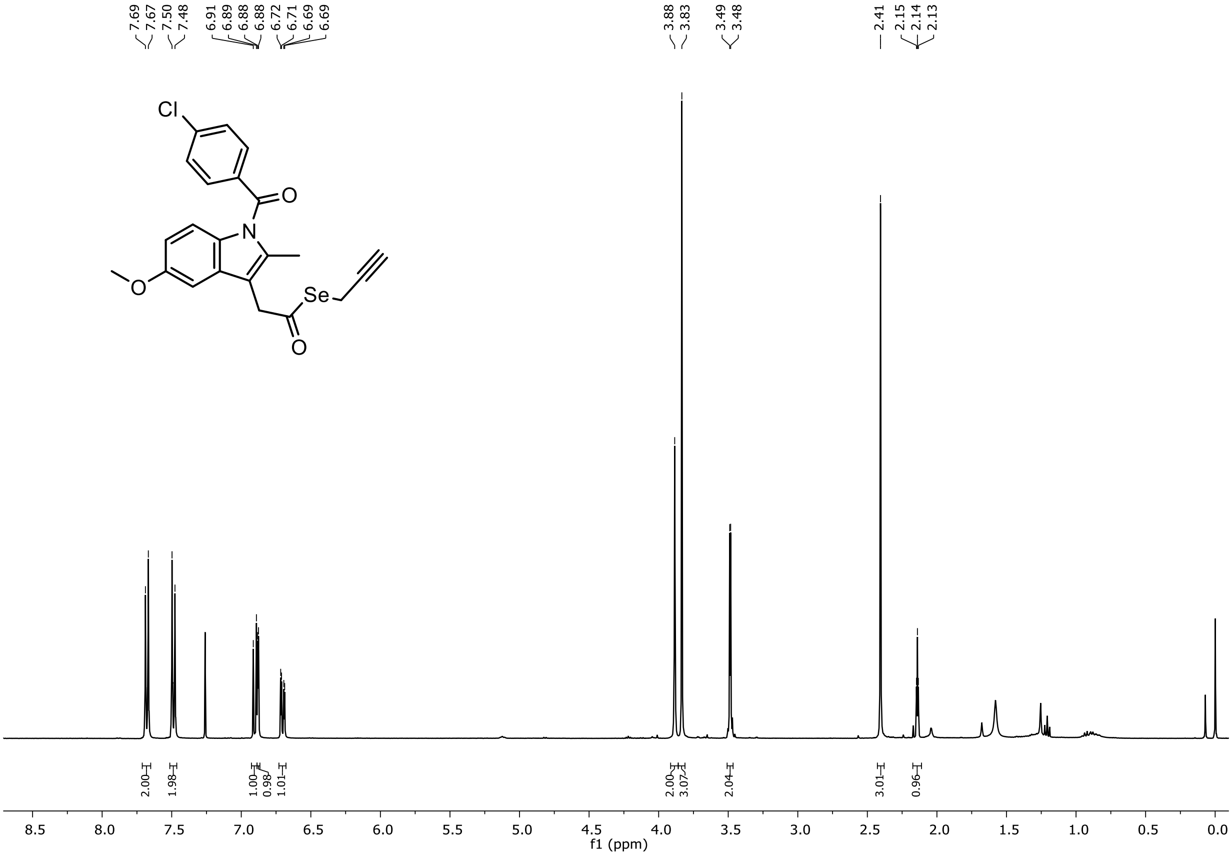

Figure S111. ^{1}H-NMR spectrum of compound **17b.**

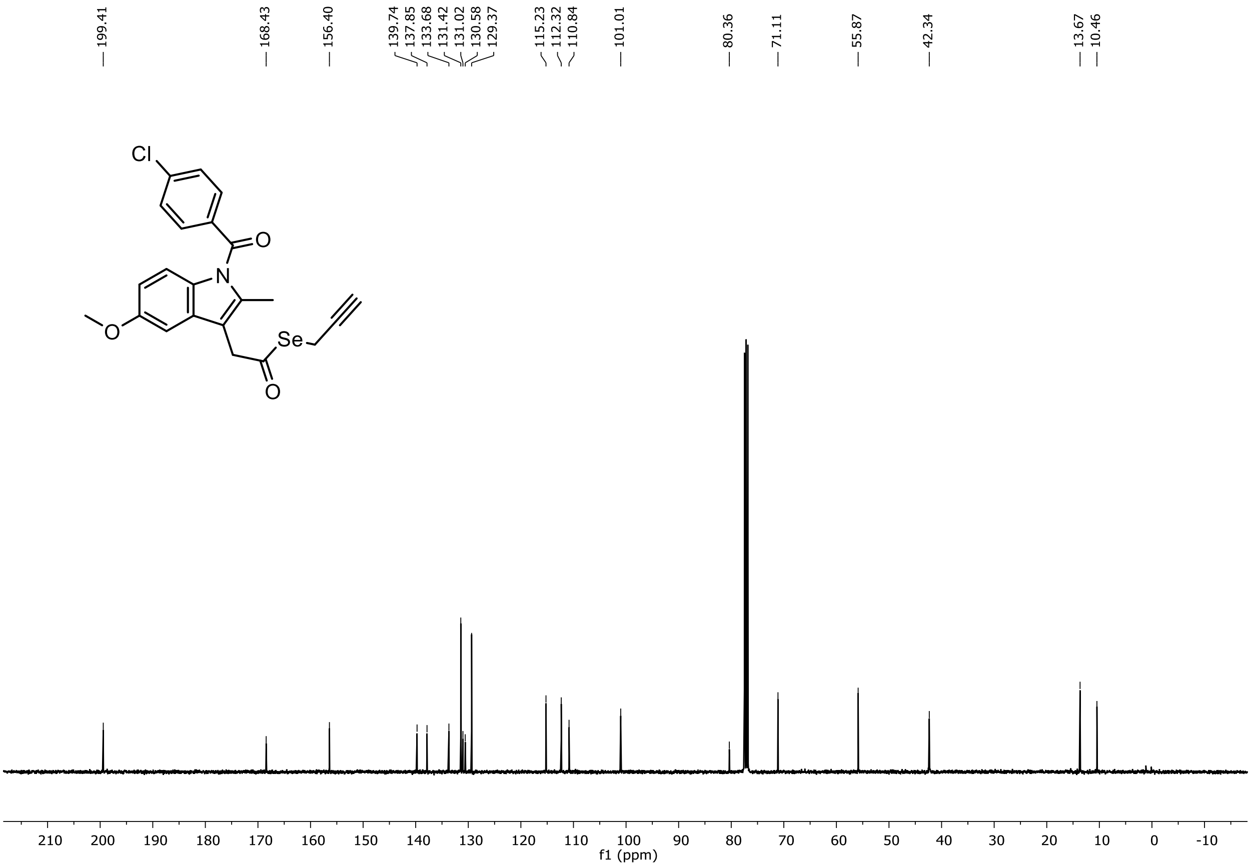

Figure S112. ^{13}C-NMR spectrum of compound **17b.**

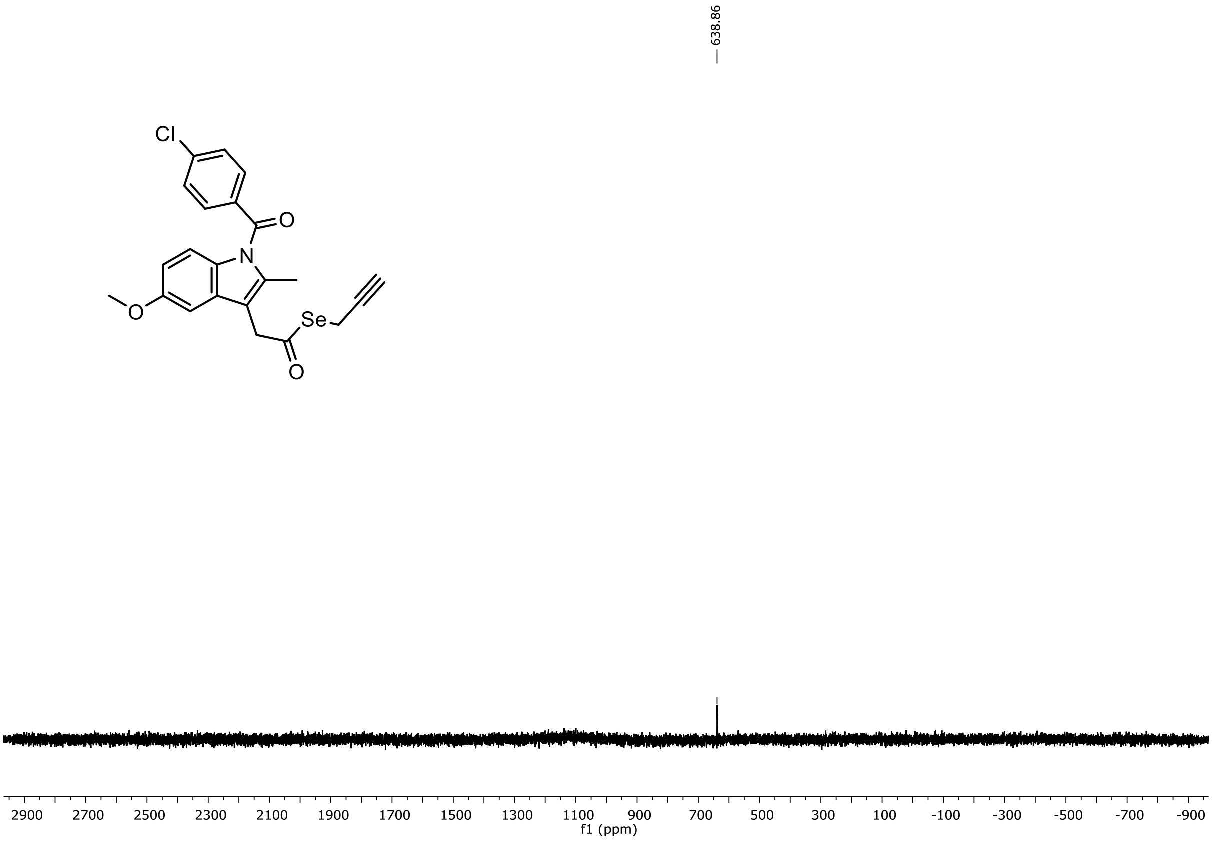

Figure S113. ^{77}Se-NMR spectrum of compound **17b.**

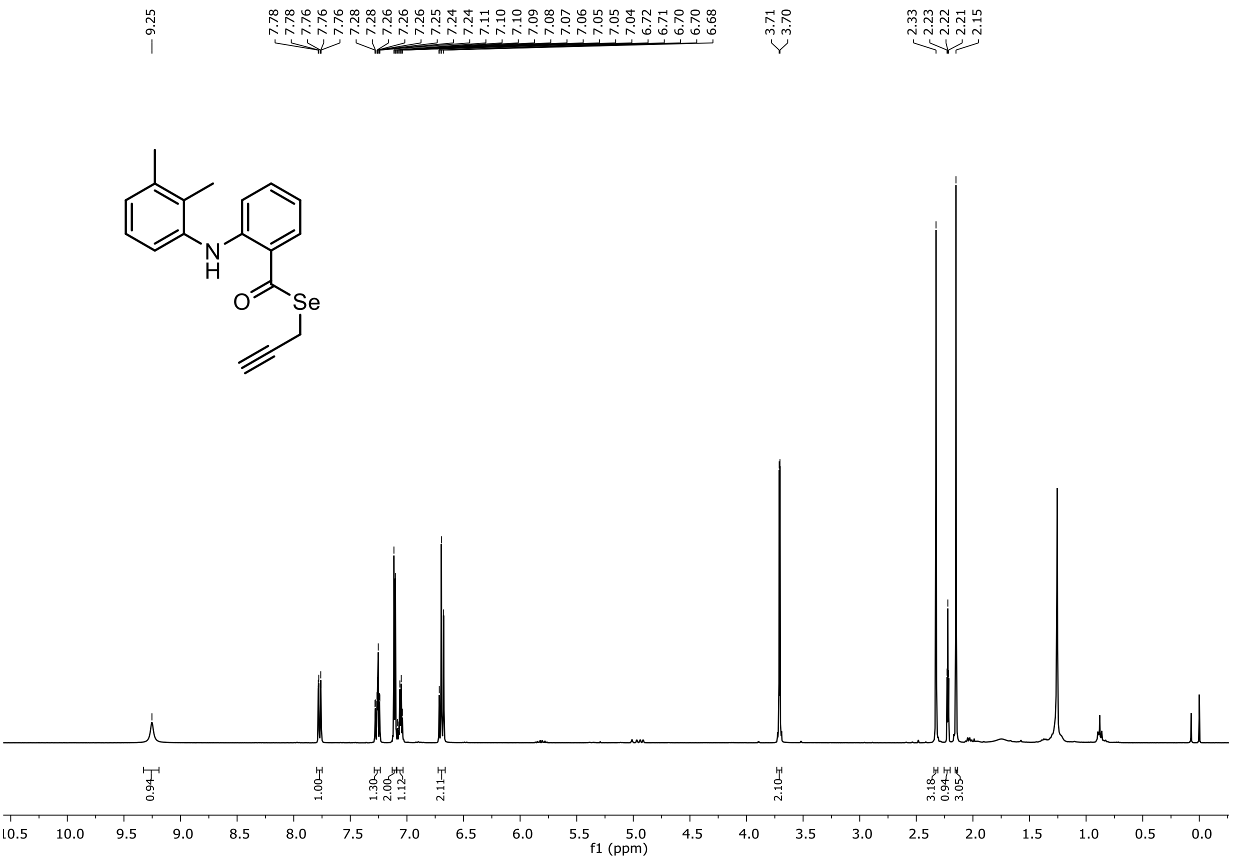

Figure S114. ^{1}H-NMR spectrum of compound **18b.**

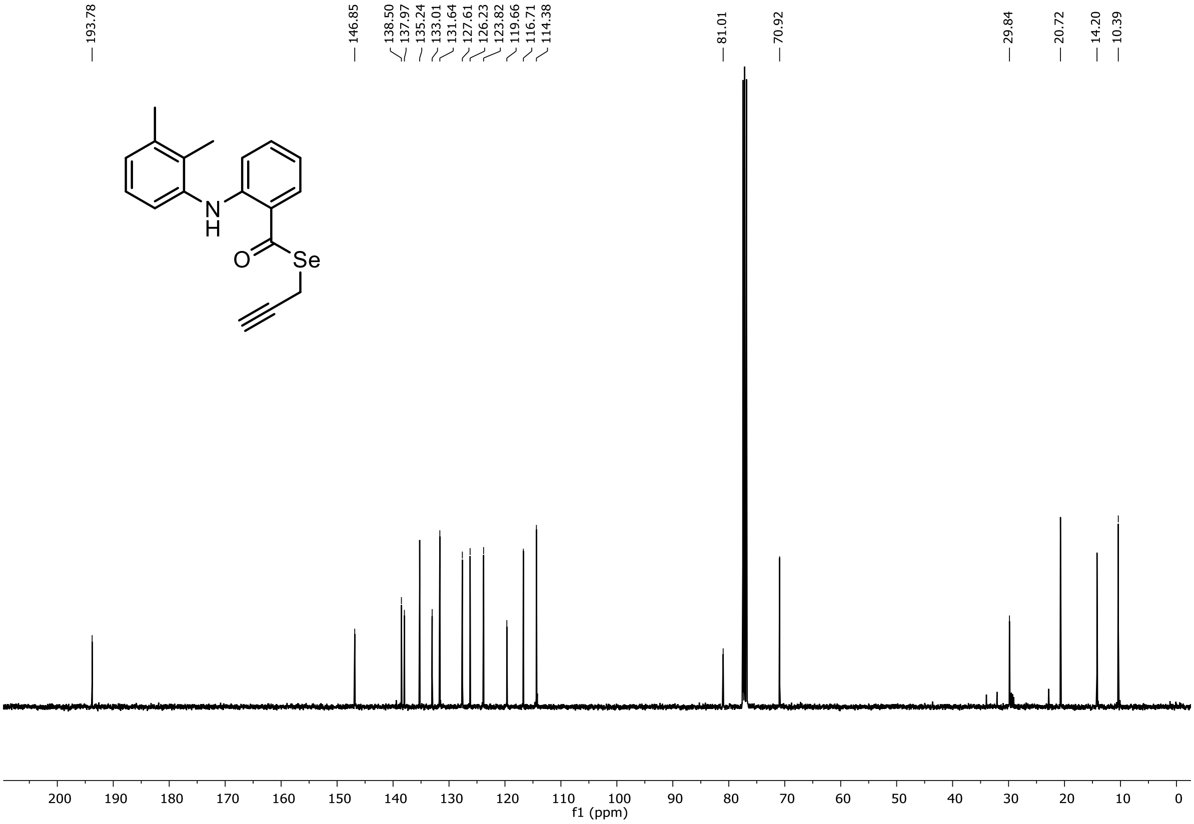

Figure S115. ^{13}C-NMR spectrum of compound **18b.**

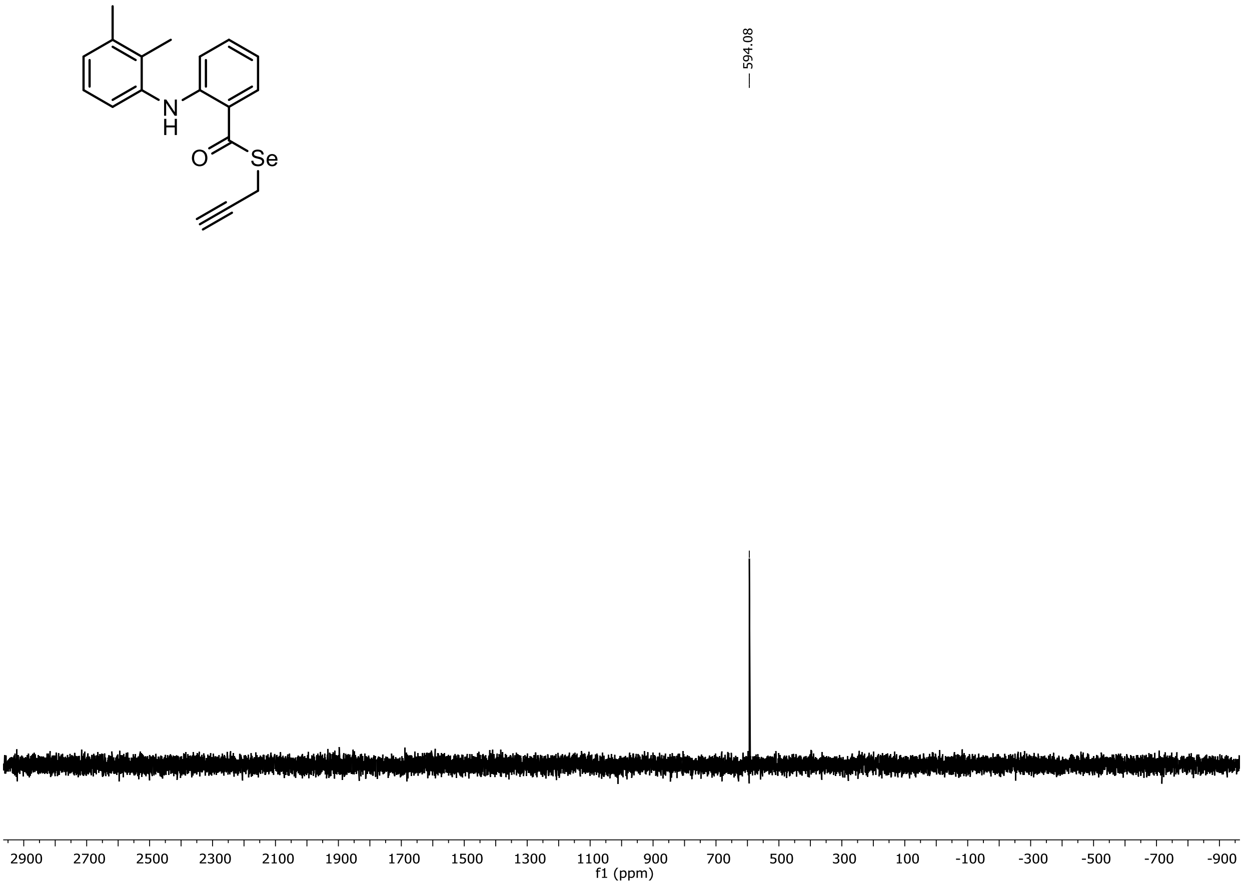

Figure S116. ^{77}Se-NMR spectrum of compound **18b.**

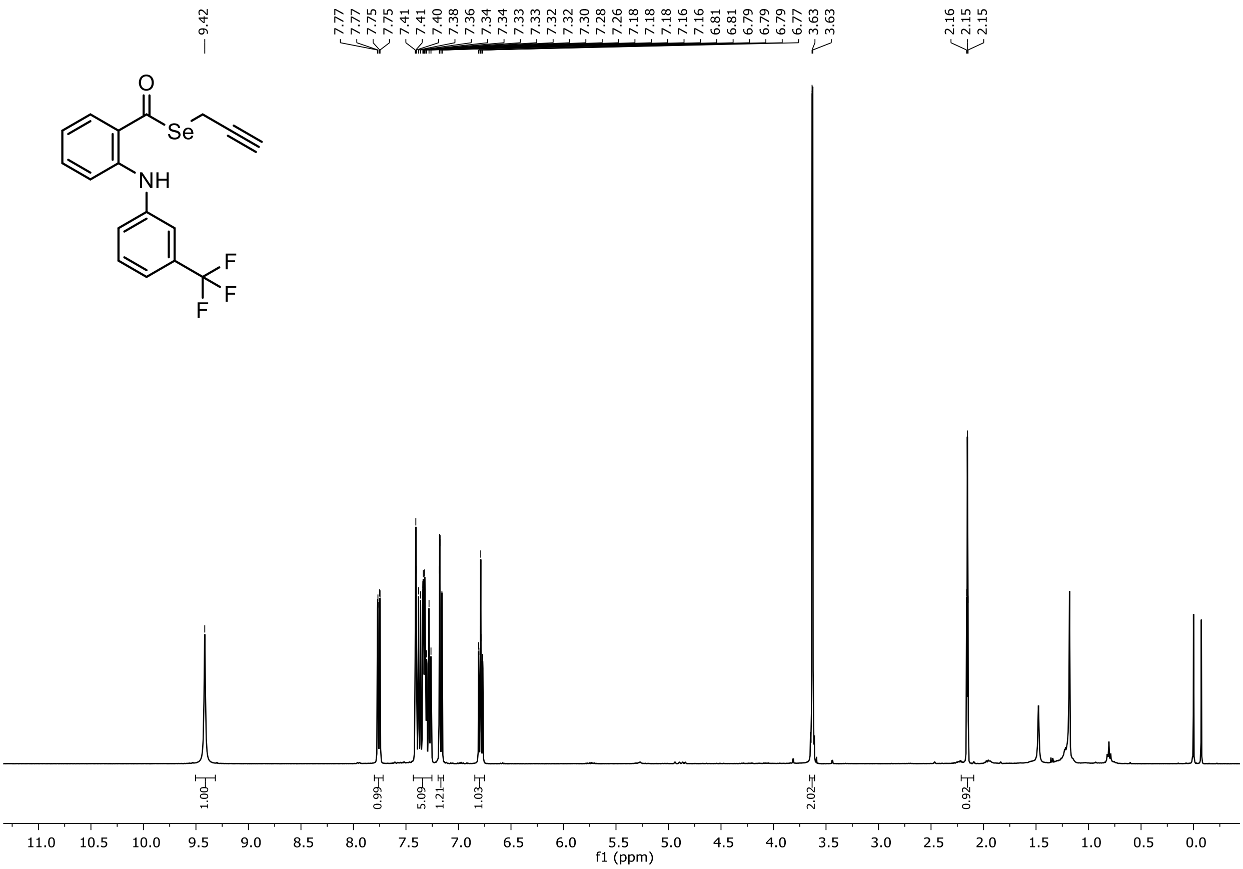

Figure S117. ^{1}H-NMR spectrum of compound **19b.**

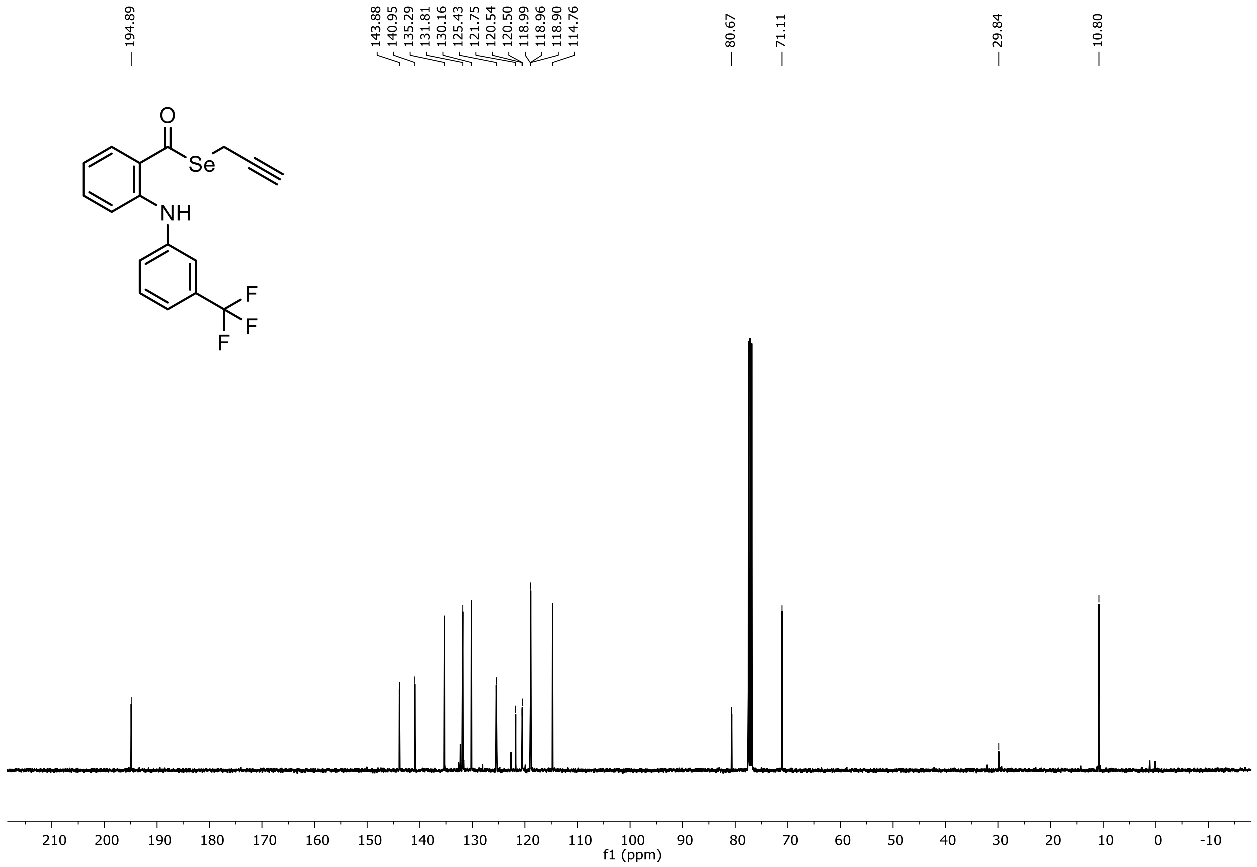

Figure S118. ^{13}C-NMR spectrum of compound **19b.**

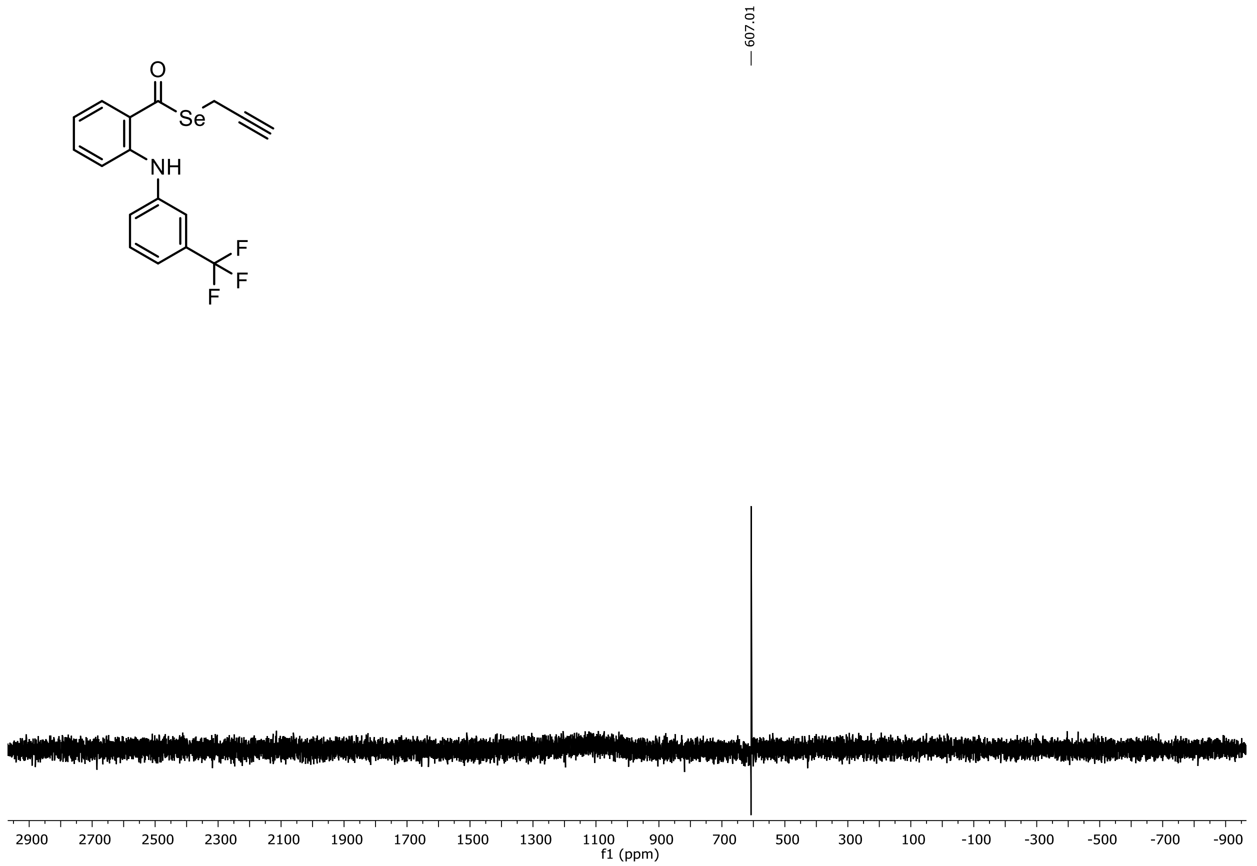

Figure S119. ^{77}Se-NMR spectrum of compound **19b.**
